# The regulatory landscape of nascent transcription in human health and disease

**DOI:** 10.1101/2025.09.24.676871

**Published:** 2025-09-26

**Authors:** Sagar R. Shah, You Chen, Alden K. Leung, Priscila V. Castilho Navarro, Mauricio I. Paramo, Juhi Gupta, Aishwarya Gurumurthy, Rebuma Firdessa Fite, Annika K. Weimer, Qian Du, Ahmed M. Mohyeldin, Dieter Egli, Remi J. Creusot, Russell J.H. Ryan, Michael P. Snyder, Andrew G. Clark, John T. Lis, Haiyuan Yu

**Affiliations:** 1 Department of Molecular Biology and Genetics, Cornell University, Ithaca, NY, USA; 2 Weill Institute for Cell and Molecular Biology, Cornell University, Ithaca, NY, USA; 3 Department of Computational Biology, Cornell University, Ithaca, NY, USA; 4 Department of Pathology, University of Michigan Medical School, Ann Arbor, MI; 5 Columbia Center for Translational Immunology and Department of Medicine, Columbia University Medical Center, New York, NY; 6 Naomi Berrie Diabetes Center, Columbia University Medical Center, New York, NY; 7 Departments of Medicine and Microbiology & Immunology, Norman Fleischer Institute of Diabetes and Metabolism, Albert Einstein College of Medicine, New York, NY; 8 Department of Genetics, Stanford School of Medicine, Stanford, CA; 9 Novo Nordisk Foundation Center for Genomic Mechanisms of Disease, Broad Institute of MIT and Harvard, Cambridge, MA; 10 Department of Pediatrics, Columbia University, New York, NY; 11 Department of Neurological Surgery, University of California, Irvine, CA

## Abstract

Transcriptional regulatory elements (TREs) orchestrate gene expression programs fundamental to cellular identity and transitions between physiological and pathological states. Decoding the regulatory logic of human biology requires resolving where, when, and how these elements are transcriptionally engaged. Here, we profiled the active transcriptional regulatory landscape across all major organ systems and a broad spectrum of developmental and disease states using PRO-cap, a high-resolution method that captures nascent transcription start sites with unprecedented sensitivity and specificity. This atlas of active TREs highlights elements shaped by their cellular contexts and evolutionary constraints, sheds light on the genetic architecture of human traits and diseases, and reveals how patterns of transcription initiation and pausing encode regulatory logic. In cancer, nascent transcription enables the delineation of lineage-specific regulatory states, metastatic adaptations, and the co-option of pre-existing programs. Together, these findings establish nascent transcription as a core dimension of gene regulation, illuminating principles that govern development, physiology, and disease.

## Introduction

Transcriptional regulatory elements (TREs), such as enhancers and promoters, shape gene expression landscapes that orchestrate development, maintain homeostasis, and underpin disease progression. While traditional annotations integrate chromatin accessibility and histone modifications, widespread nascent transcription has recently emerged as a critical marker of active TREs^1–4^. A comprehensive catalog of nascent transcriptomes across physiological and pathological states will advance our understanding of gene regulation in human health and disease.

High-resolution run-on-based methods, such as Precision nuclear Run-On sequencing with 5′-capped RNA enrichment (PRO-cap), offer exceptional sensitivity and specificity for detecting nascent transcription at TREs by mapping active RNA polymerase II at transcription start sites (TSSs) genome-wide^5^. To thoroughly delineate the regulatory landscape, we developed a streamlined and optimized PRO-cap protocol suitable for diverse human biospecimens, ranging from cadaveric tissues to clinical biopsies of both solid and liquid organs. The resulting atlas defines the architecture, evolutionary constraints, and regulatory syntax of active TREs, offering a detailed lexicon of their function and interplay across physiological and pathological contexts.

## Results

### Comprehensive mapping of active transcriptional regulatory elements by PRO-cap across development, physiology, and pathology

**Figure 1 |:**
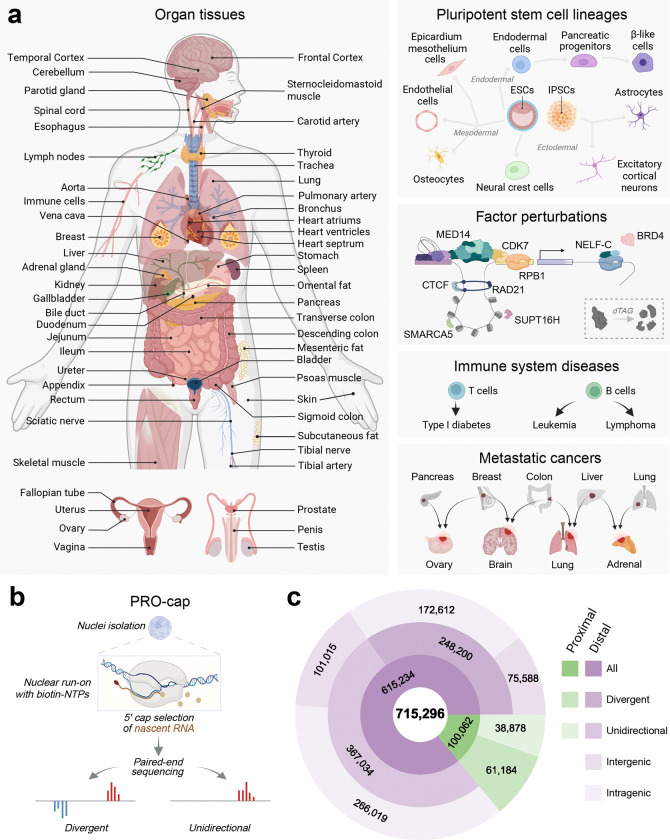
Comprehensive mapping of active transcriptional regulatory elements across human physiology and pathology **(a)** Schematic depicting the diversity of samples profiled, spanning tissues and cell types across all major human organ systems, pluripotent stem cells and their differentiated lineages, and a variety of disease states. **(b)** Schematic of PRO-cap assay to detect divergent and unidirectional TREs. **(c)** Donut plot showing the total number of TREs identified, classified by transcriptional directionality (divergent or unidirectional), distance to annotated gene TSSs (proximal or distal), and genomic context (intergenic or intragenic).

To comprehensively define the active regulatory landscape of the human genome, we performed PRO-cap on a large collection of biosamples (n=215) spanning all major human organ systems, pluripotent stem cells with their differentiated lineages, factor perturbations, and a wide spectrum of disease contexts (Fig. 1a, b; Supplementary Fig. 1; Supplementary Table 1). This unprecedented breadth and resolution of nascent transcriptome profiling enables a panoramic view of regulatory programs across human development, differentiation, and pathology. We identified 715,296 TREs, classified as proximal or distal based on their distance to known gene TSSs, corresponding to putative gene promoters (some may be proximal enhancers) and distal enhancers, respectively (Fig. 1c). Based on transcription initiation patterns, TREs were further categorized as either divergent, defined by a pair of peaks on opposite DNA strands within 300 bp of each other^6^, or unidirectional, with peaks detected on only one strand^7^. Genome-wide mapping showed that both classes are broadly distributed and preferentially enriched in gene-dense regions (Supplementary Fig. 1), suggesting a widespread role for both divergent and unidirectional elements in gene regulation.

**Extended Data Fig. 1 |:**
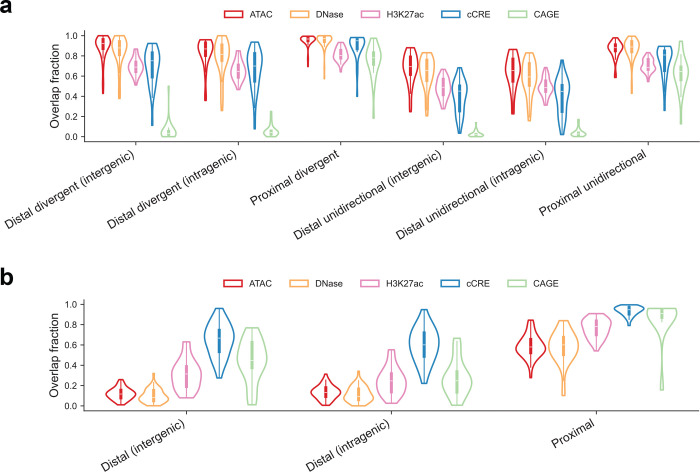
Systematic benchmarking of PRO-cap elements against other annotations **(a)** Violin plots showing the proportion of PRO-cap elements overlapping ATAC-seq, DNase-seq, H3K27ac ChIP-seq, cCREs, and CAGE-seq peaks, stratified by TRE category. Each datapoint represents the percent overlap between two datasets for a given matched tissue type. **(b)** Violin plots showing the proportion of distal and proximal elements annotated by chromatin accessibility, H3K27ac ChIP-seq, cCREs, or CAGE-seq that overlap with PRO-cap elements. Each datapoint represents the percent overlap between two datasets for a given matched tissue type.

We next performed systematic benchmarking of our PRO-cap dataset against tissue-matched epigenomic and transcriptomic datasets. Most PRO-cap elements, both distal and proximal, overlapped with peaks defined by chromatin accessibility (i.e., ATAC-seq and DNase-seq) or histone marks (i.e., H3K27ac ChIP-seq), as well as ENCODE candidate cis-regulatory element (cCRE) annotations based on canonical epigenomic features (Extended Data Fig. 1a, Supplementary Table 2). Notably, while proximal elements showed high concordance between PRO-cap and CAGE-seq, the vast majority of distal elements identified by PRO-cap were missed by CAGE-seq, which primarily detects stable, capped RNAs. This underscores the high sensitivity of PRO-cap in capturing enhancer RNAs, a class of short-lived, nascent transcripts^5^.

Reciprocal analyses confirmed consistent concordance for proximal elements across different annotations (Extended Data Fig. 1b). However, a considerable number of distal elements defined by epigenomic features did not overlap with elements detected by PRO-cap. Previous studies have shown that these non-overlapping regions often lack regulatory activity in functional assays, whereas PRO-cap signals robustly predict active enhancer function^3,4^. A similar discrepancy was observed between PRO-cap and CAGE-seq, which has been reported to detect spurious capping events from exonic regions rather than true transcription initiation sites^8,9^.

This comprehensive atlas of active TREs provides a foundational resource for decoding the architectural and functional diversity of TREs and their regulatory logic. It enables new insights into how transcription is orchestrated across human tissues, developmental states, and disease contexts.

### Motifs contributing to nascent transcription in open chromatin

**Extended Data Fig. 2 |:**
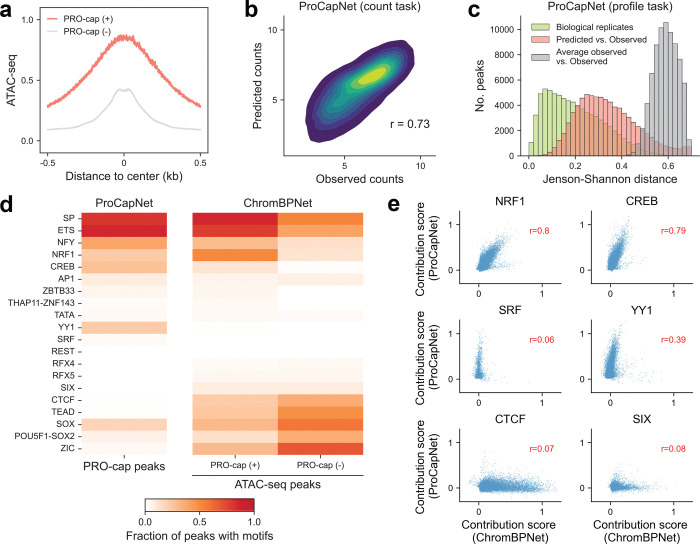
Differential motif contributions to nascent transcription and chromatin accessibility **(a)** The metaplot of ATAC-seq profiles for open chromatin regions, with and without PRO-cap signals, i.e., PRO-cap (+) and PRO-cap (−). Distances are shown as ±0.5 kb from the element center. **(b)** Density plot of log-transformed observed and predicted counts at PRO-cap peaks from held-out test chromosomes across 7-fold cross-validation, based on the model trained on ESCs. **(c)** Distribution of Jensen-Shannon distances (lower values indicate greater similarity) between observed and predicted base-resolution profiles at PRO-cap peaks from held-out test chromosomes across 7-fold cross-validation (red), based on the model trained on ESCs. For comparison, distances between observed profiles from two biological replicates of ESCs are shown in green (upper bound), and distances between observed profiles and profiles averaged over all peaks are shown in grey (baseline). **(d)** *Left panel:* Fraction of PRO-cap peaks with a given motif contributing to transcription (ProCapNet model). *Right panel:* Fraction of ATAC-seq peaks from PRO-cap (+) and PRO-cap (−) groups harboring a given motif contributing to accessibility (ChromBPNet model). **(e)** Scatterplots showing three categories of motifs with varying contributions (count task) to transcription (ProCapNet model) and accessibility (ChromBPNet model) in PRO-cap (+) regions. Each data point represents the summed contribution score for a motif instance identified by Fi-NeMo. The first and last rows highlight example motifs that predominantly contribute to transcription and accessibility, respectively. The middle row shows motifs whose contribution scores are well correlated between the two models.

As shown in our prior comparison across assays, only a subset of open chromatin regions displays nascent transcription (Extended Data Fig. 1a). Furthermore, we found that those with nascent transcription (PRO-cap [+]) exhibit greater accessibility than those without (PRO-cap [−]) (Extended Data Fig. 2a), suggesting that enhancer activation further increases chromatin openness.

To identify the regulatory factors underlying chromatin accessibility and nascent transcription, we first trained ProCapNet^10^, a deep learning model designed to predict base-resolution profiles of transcription initiation, in human embryonic stem cells (hESCs) (Extended Data Fig. 2b, c; Supplementary Table 2). By default, ProCapNet is trained on PRO-cap peaks and accessible background regions lacking transcription initiation, enabling it to learn sequence determinants of transcription initiation independent of chromatin accessibility. This design makes it particularly well suited to our goal of distinguishing factors governing these two processes. We applied TF-MoDISco^11^ to contribution scores across all PRO-cap peaks to decipher TF motif lexicons that regulate nascent transcription. These included motifs of ubiquitous TFs (e.g., SP, ETS, NFY) and well-known cell-type-specific TFs such as POU5F1-SOX2 and SOX in hESCs (Extended Data Fig. 2d).

For comparison, we leveraged the previously published ChromBPNet model trained in hESCs^12^ to examine motifs contributing to accessibility in ATAC-seq peaks with and without nascent transcription (Extended Data Fig. 2d). We identified the motif instances based on the contribution scores from ProCapNet and ChromBPNet models separately and found that TF motifs impact chromatin accessibility and nascent transcription to varying degrees. Some TFs, including SP, ETS, NFY, NRF1, and CREB, modulate both transcription and chromatin accessibility. They are also more abundant in ATAC-seq peaks with transcription compared to those without, which may partially explain the higher accessibility observed in these regions. Notably, YY1 and SRF motifs were uniquely detected by the ProCapNet model, supporting their specific involvement in nascent transcription and potentially accounting for the absence of transcription in certain open chromatin regions. Furthermore, other TFs such as ZIC, POU5F1-SOX2, and CTCF predominantly regulate chromatin accessibility, being broadly abundant across ATAC-seq peaks regardless of transcriptional status.

To further dissect the respective roles of these TF motifs in transcription versus accessibility, we visualized the contribution scores of individual motif instances across these two models. TF motifs with dual roles in both regulatory layers display stronger correlation between models, whereas those with more dominant effects on either transcription or accessibility exhibit weaker concordance (Extended Data Fig. 2e). These findings highlight the complexity of the regulatory landscape, where distinct and shared TF activities coordinate to fine-tune chromatin state and transcriptional output.

### Tissue-specific TRE usage across the human body

Given that nascent transcription requires specific sequence determinants beyond those governing chromatin accessibility, we systematically delineated transcription patterns across diverse tissue types to uncover their regulatory logic and disease relevance.

**Figure 2 |:**
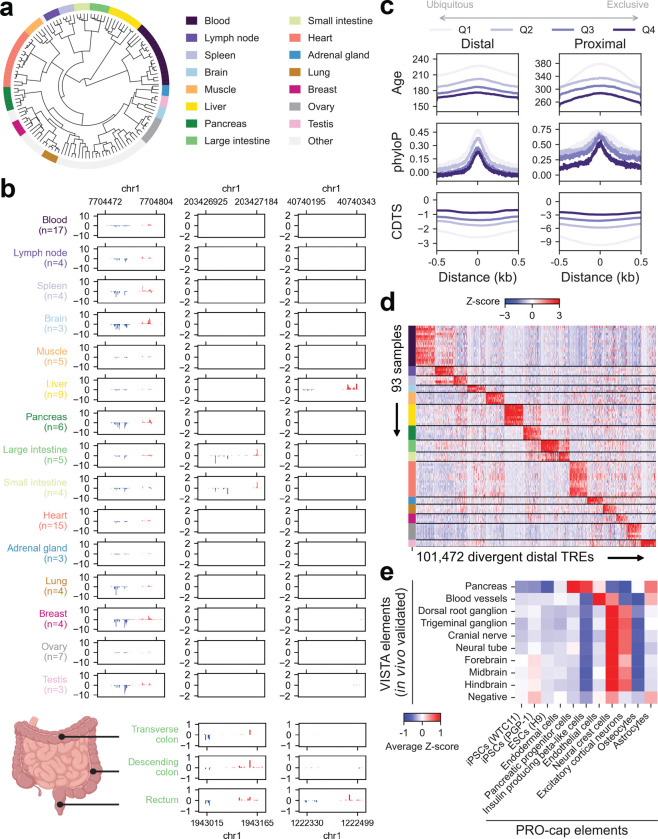
Functional and evolutionary architecture of tissue-specific transcriptional regulation across the human body **(a)** Dendrogram illustrating hierarchical clustering of tissue samples based on normalized PRO-cap expression levels of divergent distal elements. Each leaf corresponds to a sample, while subtrees primarily representing a single tissue type with at least three samples are highlighted. **(b)** *Upper panel:* Representative browsershots of PRO-cap signals from 15 tissue types highlighted in (a) across three genomic loci. Each track represents average RPM-normalized PRO-cap signals across samples of the same tissue type. *Lower panel:* Representative browsershots of PRO-cap signals from three segments of the large intestine (transverse colon, descending colon, and rectum) across two genomic loci. **(c)** Metaplots of sequence age (million years ago), phyloP, and CDTS across distal and proximal divergent elements grouped by specificity score quantiles, with Q1 indicating the most ubiquitously expressed and Q4 the least. Distances are shown as ±0.5 kb from the element center. **(d)** Heatmap depicting PRO-cap signals of tissue-specific divergent distal elements (columns) across samples (rows) highlighted in (a). Colors represent the Z-score of each element across the samples. **(e)** Heatmap showing average Z-scores of PRO-cap signals at divergent elements that overlap with elements tested in the VISTA database (either displaying positive activity in relevant tissue types or negative in all tests).

To characterize the global landscape of TRE usage, we performed hierarchical clustering using normalized PRO-cap signal from divergent distal elements (Fig. 2a, Supplementary Fig. 2). This analysis revealed robust tissue-level organization of TRE usage, with samples from functionally related tissues clustering together. For example, cardiac and skeletal muscle samples grouped into a single striated muscle cluster, while lymphatic organs, including spleen, lymph node, and appendix, formed a coherent cluster. While clustering based on unidirectional elements showed greater intra-cluster heterogeneity, it retained the capacity to distinguish major tissue types, underscoring the regulatory relevance of both classes of elements (Supplementary Fig. 2b, d).

At the individual TRE level, we observed a spectrum of usage patterns across tissues. Some elements were broadly active but often displayed variability in expression level, alternative TSS selection, and transcriptional directionality. Others were highly tissue-restricted, with activity confined to a single tissue or a limited set of related tissues (Fig. 2b, upper panel). Zooming into anatomically adjacent regions, such as segments of the large intestine, revealed both shared and segment-specific TREs, highlighting the fine-grained resolution of this approach (Fig. 2b, lower panel). Taken together, our findings establish nascent transcription as a robust readout of tissue identity that may serve as a powerful framework for dissecting TRE function in human physiology and disease.

**Extended Data Fig. 3 |:**
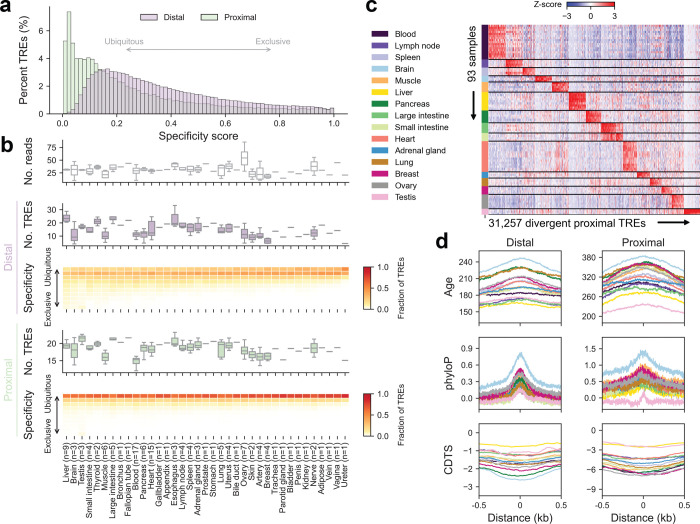
Tissue-specific regulatory element usage and evolutionary conservation patterns **(a)** Distribution of specificity scores for divergent elements. A specificity score of 0 indicates ubiquitous activity across all tissues, while a score of 1 denotes expression exclusive to a single tissue type. **(b)** Summary of TRE specificity in each tissue type. *Top panel:* Number of deduplicated uniquely mapped reads (in millions) per sample. *Second panel:* Number of detected divergent distal elements (in thousands) per sample. *Third panel:* Expression specificity of divergent distal elements, with colors indicating the fraction of elements per tissue (columns) falling into each of 10 specificity bins (rows; e.g., 0–0.1, 0.1–0.2, etc.). Fourth and *fifth panels:* Same as second and third panels, respectively, but for divergent proximal elements. The number of profiled samples is indicated alongside each tissue type. **(c)** Heatmap depicting PRO-cap signals of tissue-specific divergent proximal elements (columns) across samples (rows) highlighted in Fig. 2a. Colors represent the Z-score of each element across the samples. **(d)** Metaplots of sequence age (million years ago), phyloP, and CDTS across tissue-specific divergent elements. Distances are shown as ±0.5 kb from the element center.

To quantitatively assess tissue specificity, we computed a specificity score for each divergent element (see Methods). Overall, distal elements exhibited higher tissue specificity than proximal ones (Extended Data Fig. 3a). Despite their lower specificity, proximal elements can still effectively group major tissue types (Supplementary Fig. 2c, d). Furthermore, while most divergent distal elements were not restricted to a specific tissue type, liver, brain, and testis samples contained a higher proportion of elements with notable specificity (Extended Data Fig. 3b), consistent with previous observations^2^.

We next asked how evolutionary forces shape tissue-specific regulatory activity. Stratifying divergent elements by specificity score into four quantiles, we found that elements with the lowest tissue specificity (Q1) were the most evolutionarily conserved across species, based on phyloP scores^2^, and the least tolerant to variation in the human population, as measured by context-dependent tolerance scores (CDTS)^3^ (Fig. 2c). These findings suggest that broadly active TREs are subject to strong purifying selection. To examine selective constraints in tissue-specific programs, we identified the top 5% of divergent elements with the highest expression specificity in each tissue type (Fig. 2d, Extended Data Fig. 3c). These tissue-restricted TREs exhibited variable selective pressure: brain-specific elements were under the strongest constraint, whereas those in the liver and testis showed signatures of more rapid evolution (Extended Data Fig. 3d). These trends are concordant with findings in mouse^13^, suggesting conserved regulatory logic and turnover dynamics across mammals.

**Extended Data Fig. 4 |:**
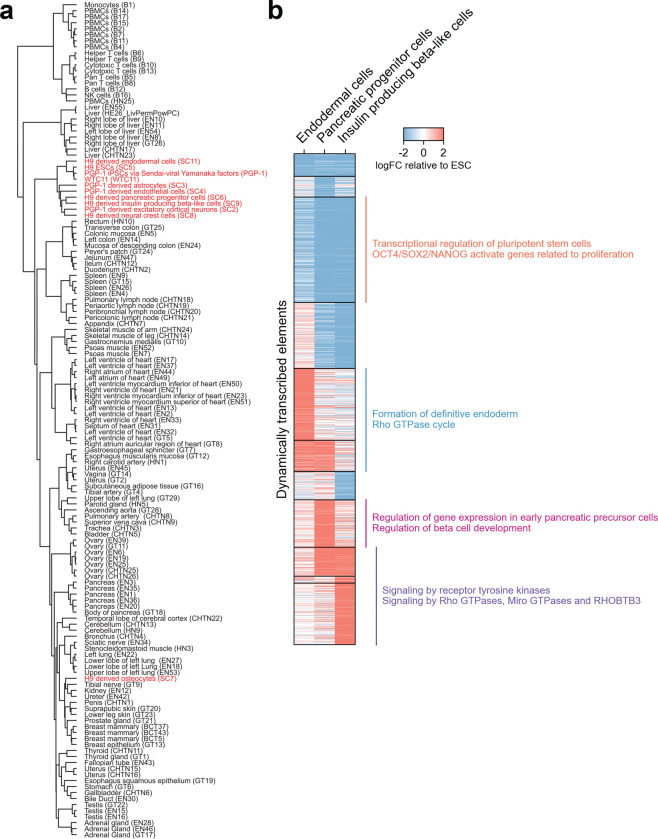
Lineage-specific transcriptional regulatory element usage during pluripotent cell differentiation **(a)** Dendrogram showing hierarchical clustering of iPSCs/ESCs and their differentiated lineages along with normal tissues based on normalized PRO-cap expression levels of divergent distal elements. **(b)** Heatmap of PRO-cap signals (relative to ESC) for divergent distal elements differentially expressed during pancreatic lineage differentiation, grouped by distinct transcriptional trajectories. Functional enrichment of predicted target genes is shown alongside each trajectory.

To understand how tissue-specific regulatory landscapes emerge, we next examined TRE dynamics across the developmental continuum from pluripotency to terminal differentiation. Hierarchical clustering revealed that iPSC- and ESC-derived cells more closely resembled pluripotent stem cells than corresponding adult tissues (Fig. 1a, Extended Data Fig. 4a). To experimentally validate TRE activity in lineage-specific contexts, we compared PRO-cap elements detected in our iPSC and ESC differentiation panel with those tested in transgenic mouse embryos from the VISTA Enhancer Browser^14^. Elements with positive enhancer activity in specific tissues showed significantly higher PRO-cap signal in the matching cell types. For example, enhancers with blood vessel activity in mouse embryos displayed elevated transcription in endothelial cells, while those associated with nervous system structures were highly transcribed in neural crest and excitatory cortical neurons (Fig. 2e).

To capture the dynamic remodeling of TRE usage during lineage progression, we profiled a time course of pancreatic differentiation, from ESCs to definitive endoderm, pancreatic progenitors, and beta-like cells. We identified divergent distal elements exhibiting significant temporal variation and clustered them into distinct transcriptional trajectories (Extended Data Fig. 4b). We linked these dynamic elements to putative target genes and performed functional enrichment analysis of the resulting gene sets. This revealed stage-specific activation of biological pathways consistent with the underlying expression dynamics. Elements associated with pluripotency declined over time, while those linked to endodermal and pancreatic lineage commitment emerged as differentiation progressed. Together, these analyses demonstrate that nascent transcriptomes measured by PRO-cap capture tissue- and cell-type-specific TRE usage and resolve temporal regulatory transitions during differentiation.

### Tissue-specific effects of disease- and trait-associated variants

To investigate the functional relevance of tissue-specific TREs, we assessed the enrichment of genetic variants associated with diseases and complex traits. Most variants identified by genome-wide association studies (GWAS) reside in noncoding regions, potentially altering TRE activity and perturbing their target gene expression in a tissue-specific manner^15,16^. Determining the relevant tissue contexts for these regulatory effects is essential for understanding physiological and disease mechanisms, as well as informing precision medicine.

**Figure 3 |:**
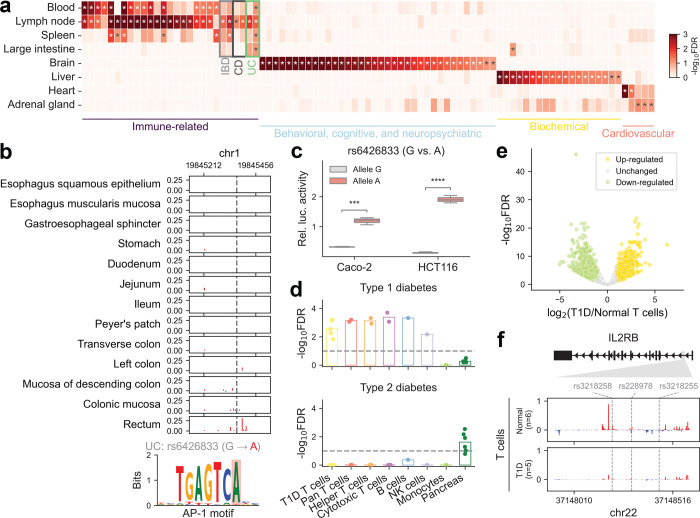
Tissue-specific effects of disease- and trait-associated variants **(a)** Heatmap showing the significance of heritability enrichment for representative human diseases and complex traits across divergent distal tissue-specific TRE annotations. Asterisks (*) denote FDR < 0.1 after Benjamini-Hochberg correction. The full panel is shown in Supplementary Fig. 3. IBD: Inflammatory bowel disease; CD: Crohn’s disease; UC: Ulcerative colitis. **(b)** Genome browser view of PRO-cap signal at a distal element across different gastrointestinal segments, harboring a fine-mapped GWAS variant (rs6426833) that alters an AP-1 motif. **(c)** Luciferase reporter assay showing enhancer activity for the risk allele (A) compared to the reference allele (G) in Caco-2 and HCT116 cells. **(d)** The significance of heritability enrichment for T1D (*upper panel*) and T2D (*lower panel*) across healthy immune cell types and pancreas, as well as T cells isolated from T1D patients. Each point represents an individual sample. The dashed line denotes an FDR threshold of 0.1. **(e)** Volcano plot showing differential PRO-cap signal at TREs of T cells from T1D patients versus non-diseased donors, with each point representing an element. TREs with significantly increased (yellow) or decreased (green) PRO-cap expression in T1D are highlighted. **(f)** Browser shot of average PRO-cap signal tracks at the *IL2RB* intronic enhancer locus in T cells from non-diseased donors and T1D patients. Fine-mapped T1D-associated variants located within this locus are marked by dashed lines.

To evaluate the contribution of tissue-specific TRE annotation to trait heritability, we applied stratified linkage disequilibrium score regression (S-LDSC)^17,18^ to 176 GWAS summary statistics spanning various human diseases and complex traits (Fig. 3a, Supplementary Fig. 3). Patterns of heritability enrichment were highly tissue-specific, recapitulating known biological processes. For example, immune-related phenotypes exhibited significant enrichment in blood and lymphoid tissues, while neuropsychiatric diseases and many traits, including intelligence, educational attainment, body mass index, and smoking status, showed pronounced enrichment in brain tissues. Likewise, liver-specific enrichment was observed for biochemical traits (e.g., levels of bilirubin, low-density lipoprotein, and sex hormone-binding globulin), consistent with the liver’s roles in metabolism, transport, and detoxification.

These tissue-specific heritability enrichment profiles also enabled the distinction of disease subtypes with overlapping etiologies. For instance, inflammatory bowel disease (IBD) includes ulcerative colitis (UC) and Crohn’s disease (CD), each with distinct clinical features, including differences in the location of inflammation. We found that both UC and CD showed enrichment in immune-related tissues; however, only UC displayed significant enrichment in the intestines, in line with its canonical disease manifestation^19,20,21^ (Fig. 3a). This tissue-specific signal was further supported at the variant level: a fine-mapped variant, rs6426833, associated with UC but not CD^22^, resides in a TRE with pronounced expression in the rectum (Fig. 3b), consistent with the clinical observation that UC inflammation typically originates in the rectum, with variable proximal extension^19^. Motif analysis predicted that the alternative allele (A) enhances binding of AP-1, a TF involved in the inflammatory response in UC^23,24^. To test this, we cloned the wild-type and mutant TREs into a luciferase reporter vector and measured enhancer activity in colorectal cancer cell lines Caco-2 and HCT116. The alternate allele consistently drove higher luciferase expression than the reference allele, confirming that this variant enhances regulatory activity in a large intestine-relevant cellular context (Fig. 3c).

**Extended Data Fig. 5 |:**
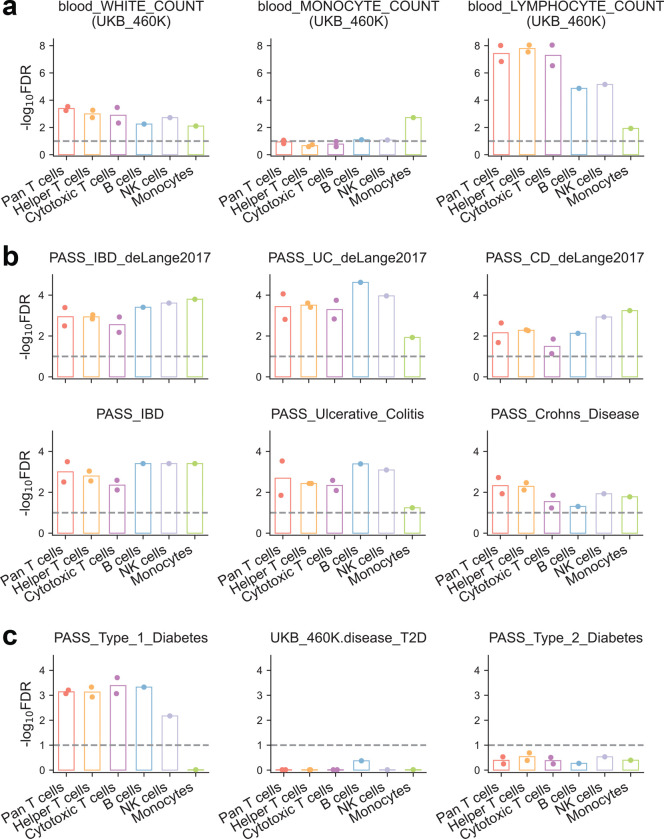
Heritability enrichment of immune-related phenotypes in diverse blood cell types **(a)** The significance of heritability enrichment for blood cell count traits across major immune cell types from healthy individuals. **(b)** Same as (a) but for IBD, UC, and CD. **(c)** Same as (a) but for T1D and T2D. Each point represents an individual sample. The dashed line denotes an FDR threshold of 0.1.

These tissue-level patterns underscore the importance of cellular context in mediating disease risk. In particular, autoimmune disorders are driven by dysregulation within specific immune cell populations. Thus, to refine our understanding of genetic contribution to phenotypic variation beyond whole blood, we leveraged cell-type-resolved TRE annotations across a diverse panel of blood-derived immune cell types. This analysis revealed that trait-associated variants are consistently enriched in the most biologically relevant immune cell populations. Specifically, significant enrichment for white blood cell count phenotype is observed across all leukocyte cell types, whereas enrichment for specific count traits (i.e., monocyte, lymphocyte) is largely restricted to their corresponding cell types (Extended Data Fig. 5a). In addition, analysis of IBD and its subtypes, UC and CD, revealed broad and pronounced heritability enrichment across all major immune cell populations (Extended Data Fig. 5b). This pervasive enrichment pattern reinforces the complex and multifaceted immune dysregulation that characterizes IBD pathogenesis^25^.

**Extended Data Fig. 6 |:**
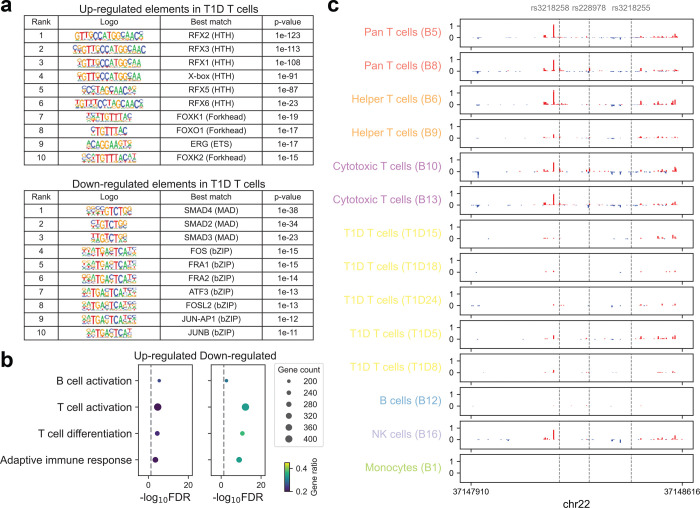
Functional characterization of differentially expressed transcriptional regulatory elements in T1D patient-derived T cells. **(a)** Enrichment of TF binding motifs in differentially expressed TREs between T1D patient- and non-diseased donor-derived T cells. Top 10 significantly enriched motifs in upregulated and downregulated TREs are shown (FDR < 0.05). Sequence logos with their corresponding TF identity match (TF name and DNA-binding domain) and p-value are shown. **(b)** Pathway enrichment analysis of genes linked to differentially expressed TREs. Dot size indicates the number of genes per pathway, and dot color represents the gene overlap ratio. **(c)** Browser shot of PRO-cap signal tracks at the *IL2RB* intronic enhancer locus in T cells from individual T1D patients and in different immune cell types from each non-diseased donor. Same locus as shown in Fig. 3f. Dashed lines indicate fine-mapped T1D-associated variants located within this region.

Furthermore, our analyses of type 1 and type 2 diabetes (T1D and T2D, respectively) highlighted their distinct pathogenic mechanisms. T1D, driven by autoimmune dysfunction, exhibited strong enrichment in T cell-associated TREs, as well as in B and natural killer (NK) cells, but minimal enrichment in monocytes (Fig. 3d). In contrast, T2D, a metabolic disease, showed its strongest enrichment in pancreatic tissue (Fig. 3d, Extended Data Fig. 5c). Building upon this, we generated PRO-cap libraries from T cells isolated from patients with T1D, enabling direct interrogation of disease-relevant regulatory activity. We observed significant heritability enrichment of T1D, but not T2D, among TREs active in patient-derived T cells (Fig. 3d). We next identified a set of differentially expressed TREs between T cells from T1D patients and non-diseased donors (Fig. 3e). Motif enrichment analysis of these TREs revealed significant enrichment for binding sites of TFs central to T cell function and autoimmune pathogenesis, including members of RFX, FOX, SMAD, and AP-1 families (Extended Data Fig. 6a, Supplementary Table 3). Pathway enrichment analysis demonstrated that differentially expressed TREs in T1D T cells were significantly associated with core T cell processes and immune response pathways (Extended Data Fig. 6b).

We next examined whether these differentially expressed TREs harbor variants associated with T1D. Notably, an intronic enhancer locus within the *IL2RB* (interleukin-2 receptor beta) gene, harboring several fine-mapped variants from the same credible set (CS)^26^, exhibited robust transcriptional activity in healthy donor T cells but showed significantly reduced activity in T1D patient-derived T cells (Fig. 3f, Extended Data Fig. 6c). Given the role of *IL2RB* in T cell homeostasis and immune regulation, reduced enhancer activity may contribute to altered IL-2 signaling in T1D. This dysregulation could have implications for disease progression and responsiveness to IL-2-based therapies.

**Extended Data Fig. 7 |:**
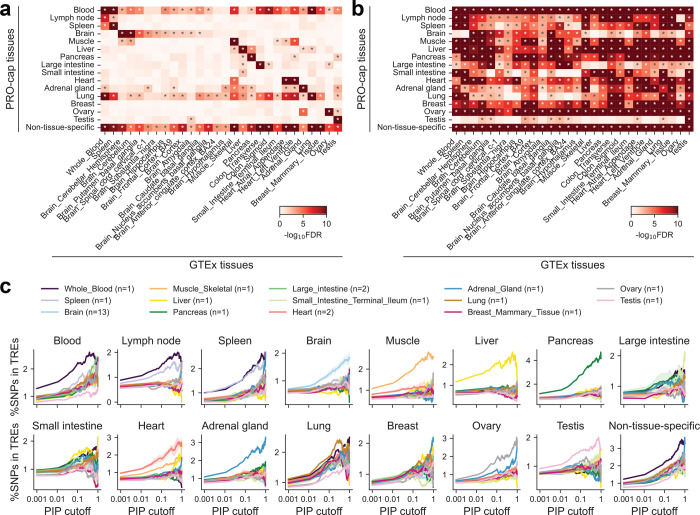
eQTL enrichment across transcriptional regulatory element annotations **(a)** Heatmap showing the significance of eQTL enrichment across divergent distal tissue-specific and non-tissue-specific TRE annotations. Asterisks (*) denote FDR < 0.05 after Benjamini-Hochberg correction. **(b)** Same as (a), but for divergent proximal elements. **(c)** Fraction of fine-mapped eQTL variants (above a given PIP threshold) overlapping tissue-specific and non-tissue-specific divergent distal elements. Lines show the average across related GTEx tissues; subplot titles indicate TRE categories. Traces are shown for thresholds with ≥5 variants. Shaded areas denote standard error.

Following the demonstration of heritability enrichment in context-specific TREs, we evaluated their role in mediating genetic effects on gene expression across tissue types. To this end, we analyzed cis-expression quantitative trait loci (cis-eQTLs) from GTEx^27^ using TORUS^28^ and observed significant enrichment in distal elements from the corresponding tissue types (Extended Data Fig. 7a). In contrast, proximal elements showed broader enrichment across tissues (Extended Data Fig. 7b). As an orthogonal approach, we assessed whether fine-mapped variants stratified by different posterior inclusion probability (PIP) thresholds preferentially overlap tissue-specific distal TREs. Indeed, variants with higher PIP values exhibited increased localization to tissue-matched TREs (Extended Data Fig. 7c). Interestingly, non-tissue-specific elements were pervasively enriched for eQTLs across tissues but showed little enrichment for specific GWAS phenotypes (Supplementary Fig. 3, Extended Data Fig. 7).

Collectively, these results indicate that tissue-resolved PRO-cap annotations enable systematic interpretation of disease- and trait-associated variants across both organismal (GWAS) and molecular (QTL) phenotypes.

### Tissue-specific modeling of nascent transcription

Building on these insights, we next aimed to elucidate the sequence features underlying transcription initiation and to quantitatively model tissue-specific effects of regulatory variants. To this end, we trained ProCapNet models to predict base-resolution PRO-cap profiles across multiple tissue types (Supplementary Fig. 4, Supplementary Table 2). Principal component analysis (PCA) of each model’s embeddings showed that the first two components explained 70–80% of the variance across all PRO-cap peaks and clearly separated TRE categories, such as distal versus proximal and unidirectional versus divergent elements (Supplementary Fig. 5). The ability of the models to distinguish TRE classes in the latent space reflects their effective learning of biologically meaningful sequence features.

**Figure 4 |:**
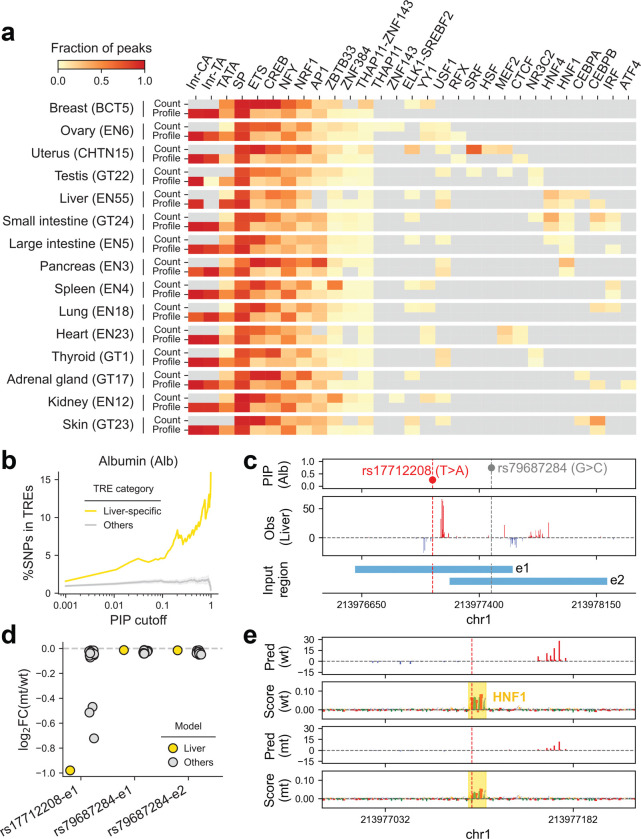
Tissue-specific modeling of nascent transcription **(a)** Fraction of PRO-cap peaks containing TF motifs identified by ProCapNet as contributing to transcriptional strength (count task) and/or transcription start site positioning (profile task) across multiple tissue types. **(b)** Fraction of fine-mapped GWAS variants for albumin measurement (above a given PIP threshold) overlapping divergent distal elements from liver-specific and other TRE categories. **(c)** The first track shows PIP values for two GWAS variants in a credible set associated with albumin measurement. The second track presents the PRO-cap signal observed in the liver sample (EN55). The final track displays the 1-kb input regions centered on each PRO-cap element, with different variants used for prediction. **(d)** Predicted impact of variants rs17712208 and rs79687284 on transcription levels of elements e_1_ and e_2_, measured by log_2_ fold change between alternative and reference alleles in liver and other tissue models. **(e)** Predicted transcription profiles and contribution scores for element e1 with either the reference or alternative allele of rs17712208 (red dashed line) using the liver-trained model.

At the motif level, Initiators (Inr-CA, Inr-TA) and the TATA box more frequently influenced TSS positioning (profile task), whereas ETS, CREB, and NRF1 instances predominantly affected transcription levels (count task) (Fig. 4a), consistent with previous observations^10^. When comparing across models, some TF motifs (e.g., SP, ETS, CREB) were ubiquitously present, while others (e.g., SRF, MEF2, HNF1) were more restricted to certain tissue types, reflecting their potential roles in shaping enhancer logic within tissue-specific transcriptional programs.

Given that GWAS variants exhibit tissue-specific effects (Fig. 3a, Fig. 4b) and our models capture the corresponding sequence syntax, we applied them to assess context-dependent regulatory variant impact. Notably, tissue-specific modeling helped refine causal inference within GWAS CSs. For example, statistical fine-mapping of the GWAS trait, albumin measurements, identified a 95% CS containing two variants (Fig. 4c). The variant rs17712208, with a lower PIP of 0.249, is in a liver-specific TRE. *In silico* mutagenesis using the liver-trained model revealed that the alternative allele disrupts the HNF1 motif, reducing transcription at TRE e1 (Fig. 4d, e). This effect was largest in the liver and absent in models from most other tissues. By contrast, the variant rs79687284, with a higher PIP of 0.750, has little effect on transcription at either TRE, e1 or e2, across all models.

This integrated analytical framework demonstrates that combining nascent transcriptome data with statistical fine-mapping and context-specific deep learning enables robust functional interpretation of noncoding variants.

### Transcription initiation and pausing patterns reveal biological roles and regulatory mechanisms

**Figure 5 |:**
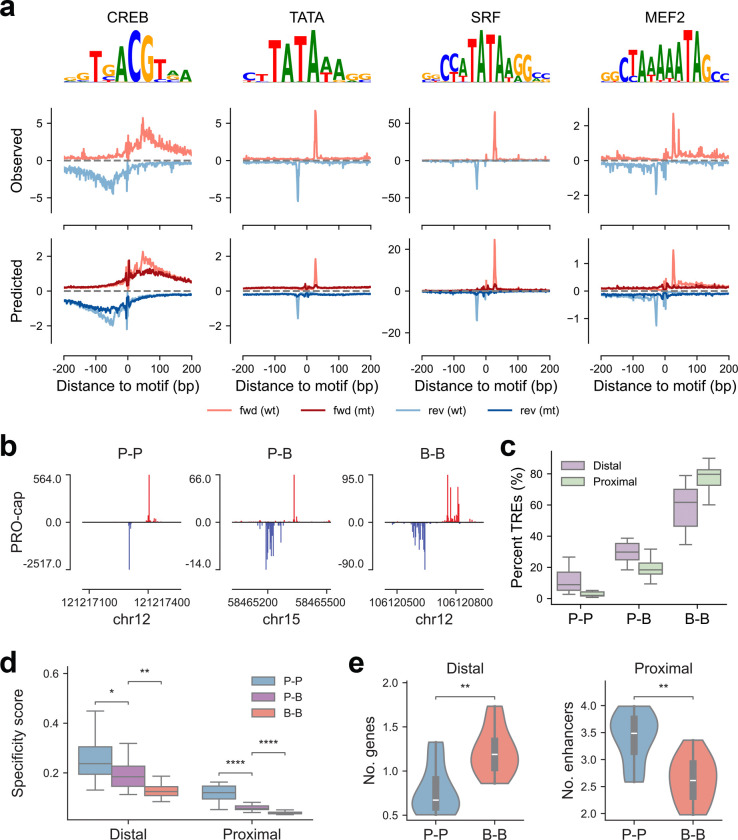
Functional implications of transcriptional peak shape in gene regulation **(a)** Representative motifs showing broad (CREB) and focused (TATA-box, SRF, and MEF2) effects. *Left panel*: contribution weight matrix from the count task of TF-MoDISco output. *Middle panel*: average observed PRO-cap profiles centered at motif instances, accounting for motif orientation. *Right panel*: average predicted profiles scaled by total counts before and after *in silico* motif deletion, centered at motif instances with motif orientation considered. One illustrative ProCapNet model is shown per motif: CREB (EN55), TATA (BCT5), SRF (GT22), and MEF2 (EN23). **(b)** Representative PRO-cap signal tracks from liver for divergent distal elements with P-P, P-B, and B-B peak shapes. **(c)** Boxplot showing percent distribution of TRE types (P-P, P-B, B-B) across tissues. Each point represents one tissue type. **(d)** Boxplot showing tissue specificity scores for divergent distal and proximal elements across the three peak shape categories. Each point represents the median score for a given tissue type. **(e)** *Left panel:* Violin plots showing the number of predicted target genes for distal elements with different peak shapes. Each datapoint represents the mean value for a given tissue type. *Right panel:* Violin plots showing the number of enhancers linked to genes with proximal elements of different peak shapes. Each point reflects the mean value for a given tissue type.

While most motifs showed broad effect curves (e.g., CREB), a few exhibited strong positional effects. Notably, SRF and MEF2 display focused peaks similar to the TATA-box, likely due to their shared TA-enriched core sequences (Fig. 5a). These motif-level differences may contribute to the overall peak shape of TREs, leading us to ask whether variation in transcription initiation patterns carries biological significance in terms of regulatory function and evolutionary constraint. Therefore, we characterized the initiation site architecture by calculating the shape index (SI) for divergent TREs using their 5’ PRO-cap signal distribution across both strands^29,30^. Based on SI values, elements were classified as Peaked (P) or Broad (B), resulting in three categories per divergent element: P-P, P-B, or B-B (Fig. 5b). This peak shape categorization revealed strong associations with gene regulatory function and expression breadth.

Across tissues, most TREs exhibited broad initiation patterns (B-B), with P-P elements being relatively rare overall but more frequent in distal than proximal regions (Fig. 5c). In addition, proximal B-B elements were broadly active across tissues, whereas P-P elements exhibited high tissue specificity (Fig. 5d, Supplementary Fig. 6), consistent with prior work showing that broad promoters are enriched at housekeeping genes^29,30^. Notably, this trend extended to distal elements, where B-B regions also displayed more ubiquitous activity across tissues. Furthermore, B-B elements (both proximal and distal) showed stronger evolutionary constraints (Supplementary Fig. 7), recapitulating the inverse relationship between tissue specificity and evolutionary conservation observed in Fig. 2d. To explore regulatory connectivity, we analyzed enhancer-promoter associations using the E2G^31^ model in various tissues (Supplementary Table 2). Distal B-B elements were linked to a greater number of putative target genes, whereas genes with proximal B-B elements tended to be regulated by fewer enhancers (Fig. 5e, Supplementary Fig. 8). These findings indicate that transcription initiation profiles offer an additional layer of biological insights beyond what can be resolved by total read counts at individual elements alone.

Having characterized initiation shape, we next asked whether transcriptional elongation dynamics vary systematically across tissues. Thus, we leveraged the base-pair resolution of PRO-cap to measure pause distances, the distance between the TSS (5’) and the downstream point of RNAPII pausing (3’) at single-molecule level, across tissues. Pause distances varied across tissues, revealing three general classes: early (short), intermediate, and late (long) pausing (Supplementary Fig. 9). This suggests that pause distance could be a stable, tissue-intrinsic regulatory feature.

**Extended Data Fig. 8 |:**
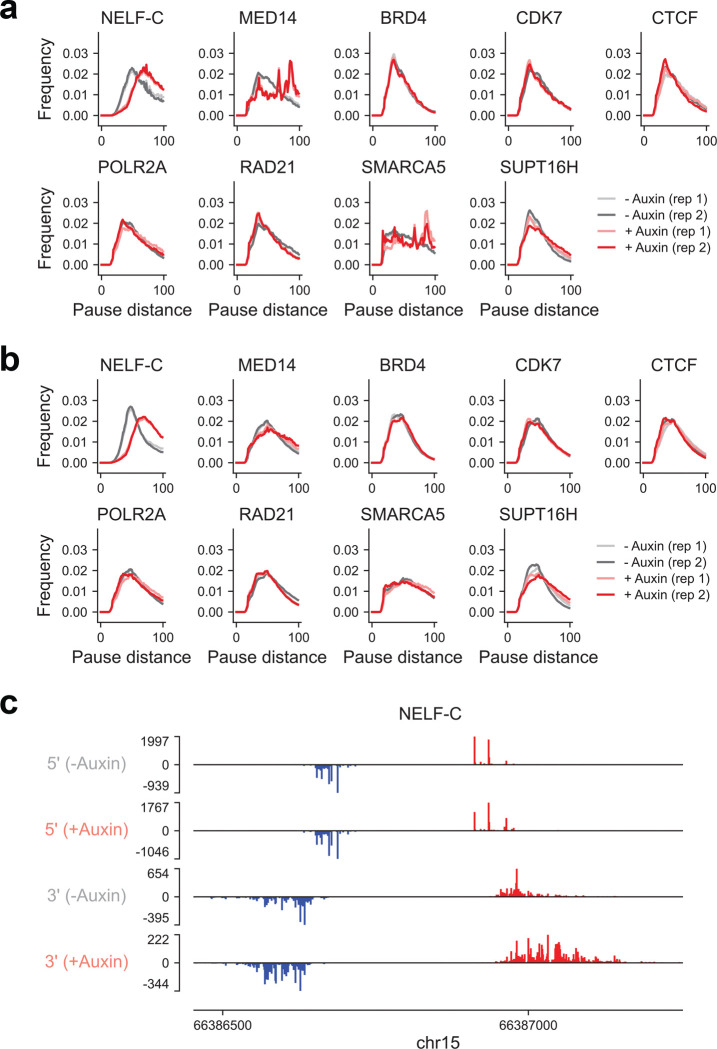
Genome-wide distribution of pause distances in transcriptional regulatory elements following depletion of different factors **(a)** Genome-wide distribution of pause distances for distal elements before and after acute depletion of the indicated factors using auxin-inducible degron systems. **(b)** Same as (a), but for proximal elements. **(c)** Representative 5’ and 3’ PRO-cap signal tracks (merged across replicates) of an element before and after NELF-C degradation in DLD-1 cells.

To identify regulators of genome-wide Pol II pause site positioning, we conducted a targeted screen and integrated publicly available datasets to assess effects of candidate factors using degron-tagged alleles. While acute depletion of most factors did not substantially alter pause distances, perturbation of NELF-C resulted in marked shifts (Extended Data Fig. 8). Loss of NELF-C led to a global increase in pause distances, consistent with its role in stabilizing promoter-proximal pausing^32^. Further studies are warranted to determine how these regulatory influences vary across cell states and tissues, and whether they contribute to the observed diversity in pause profiles across the human body.

### Subtype-specific transcriptional landscape of leukemia and lymphoma

**Figure 6 |:**
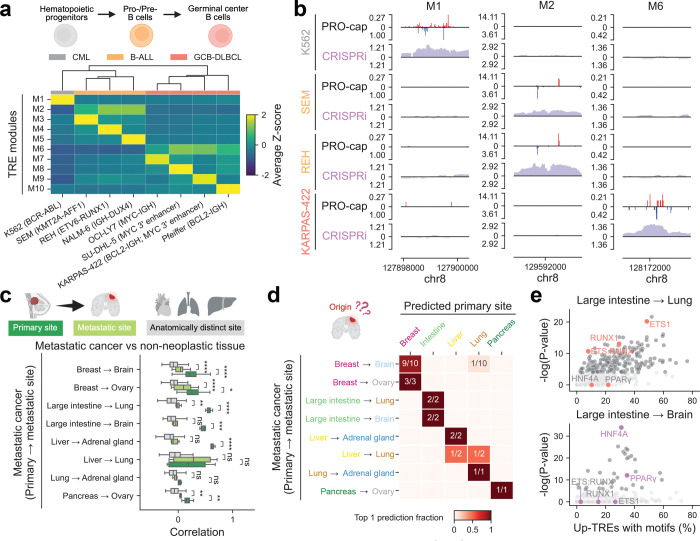
Transcriptional regulatory element signatures delineate tumor identity and organ-specific metastatic trajectories **(a)** Hierarchically clustered heatmap of CML, B-ALL, and GCB-DLBCL subtypes based on the expression patterns of divergent distal elements. The heatmap displays average Z-scores of elements within each TRE module across samples. Key pathogenic mechanisms (e.g., fusion proteins and overactivation of the MYC 3′ enhancer) are labeled alongside the corresponding cell line names. An illustration of hematopoetic developmental stages is included, with colors corresponding to the putative cellular origin of CML (equivalent to progenitors), B-ALL, and GCB-DLBCL. **(b)** Genome browser tracks of PRO-cap signal and CRISPRi scores for three representative 2.5-kb loci from TRE modules M1, M2, and M6 across four cell lines. **(c)** *Top:* Schematic overview of anatomical sites profiled, including metastatic tumor biopsies and non-neoplastic samples from their corresponding primary and metastatic sites, as well as other unrelated tissues. *Bottom:* Pairwise comparison of divergent distal TRE expression profiles between metastatic tumors and non-neoplastic tissues from the corresponding primary, metastatic, and unrelated sites. **(d)** Top-1 prediction of the primary site for metastatic tumors using a linear support vector machine classifier. **(e)** TF motif enrichment in differentially expressed TREs between lung and brain metastases from colorectal cancer.

Given the prominent regulatory specificity observed in immune cells, we next asked whether similar principles extend to hematologic malignancies. In particular, leukemias and lymphomas offer a powerful model to investigate subtype-specific enhancer usage, as they arise from distinct developmental stages of hematopoietic lineages and are characterized by diverse oncogenic drivers. Therefore, we employed PRO-cap to map the nascent transcriptomes in a panel of hematologic malignancy models comprising one chronic myelogenous leukemia (CML) cell line (K562), three B-cell acute lymphoblastic leukemia (B-ALL) cell lines (SEM, REH, and NALM-6) and four germinal center B-cell-like diffuse large B-cell lymphoma (GCB-DLBCL) lines (OCI-LY7, SU-DHL-5, KARPAS-422, and Pfeiffer), each harboring distinct cytogenetic features, including oncogenic fusion proteins^33–38^ and aberrant activation of the *MYC* 3′ enhancer^39^. Hierarchical clustering of divergent distal TREs separated hematologic malignancies in line with their origins at distinct stages of blood cell maturation (Fig. 6a).

**Extended Data Fig. 9 |:**
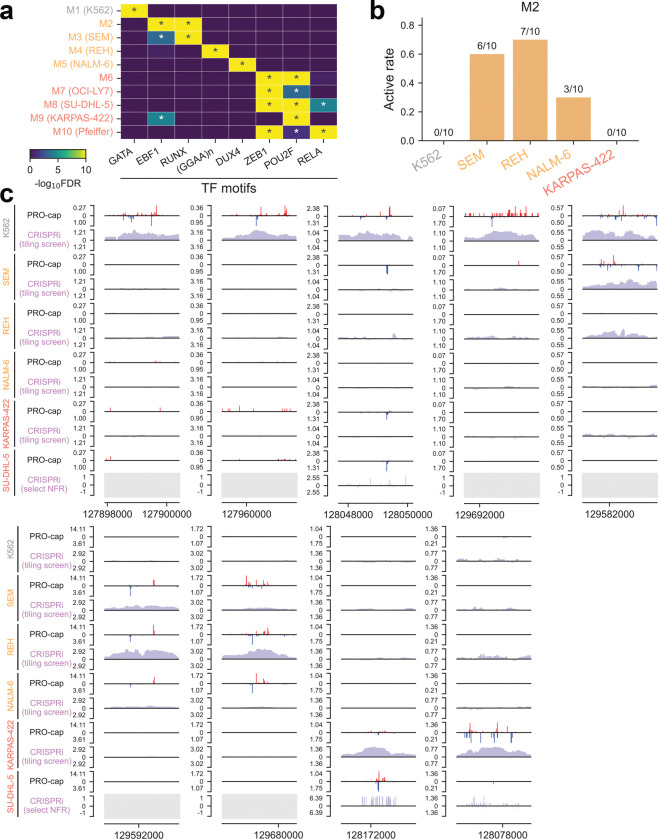
Subtype-specific transcriptional landscape of leukemia and lymphoma **(a)** Heatmap showing enrichment patterns of top *de novo* TF motifs in each subtype-specific TRE module from Fig. 6a. **(b)** Active rate of divergent distal elements in TRE module M2 across different cell lines based on CRISPRi tiling screen at the *MYC* locus. **(c)** Genome browser tracks of PRO-cap signal and CRISPRi scores for distal elements in the *MYC* locus across different cell lines, with each example covering a 2.5-kb region on chromosome 8. Grey boxes cover regions that were not targeted by the sgRNA library used for SU-DHL-5, which was different from the tiling sgRNA library used for other cell lines. NFR: nucleosome-free region.

To further resolve subtype-specific regulatory programs, we performed k-means clustering and identified ten TRE modules with coordinated activity patterns. While modules M2 and M6 were shared across all B-ALL and GCB-DLBCL lines, respectively, the majority were unique to individual subtypes (Fig. 6a). *De novo* motif enrichment analysis revealed TF motifs specific to each module (Extended Data Fig. 9a, Supplementary Table 3), reflecting distinct mechanisms of regulatory dysfunction. For instance, M4, specific to REH cells carrying the ETV6-RUNX1 fusion, was enriched for GGAA repeats, consistent with recent findings that loss of ETV6-mediated repression at GGAA microsatellite enhancers leads to aberrant gene activation^40^. Similarly, modules M8 and M9, specific to SU-DHL-5 and KARPAS-422, respectively, were enriched for POU2F motifs. Both cell lines exhibit dependence on a recently identified *MYC* 3′ enhancer regulated by a transcriptional triad (POU2F2, MEF2B, and POU2AF1) and show elevated POU2F2 expression relative to other GCB-DLBCL lines^39^.

To evaluate the functional importance of these modules in a subtype-specific manner, we conducted CRISPR interference (CRISPRi) screens at the *MYC* locus across six representative cell lines (one CML, three B-ALL, and two GCB-DLBCL). Silencing of module-specific TREs (e.g., M2) resulted in a marked reduction of *MYC* expression in the corresponding lines (e.g., SEM, REH, and NALM-6), with minimal impact on other subtypes (Fig. 6b, Extended Data Fig. 9b,c), confirming their selective regulatory function in distinct malignant contexts.

Together, these results demonstrate the value of TRE profiling for understanding lineage and oncogenic dependencies in hematologic cancers. To test the generalizability of this strategy, we next explored its application in the context of metastatic solid tumors.

### Tissue-specific TRE profiles inform primary site prediction in metastatic cancers

The clinical management of metastatic cancers can be complicated by the difficulty in identifying their tissue of origin - an issue that is especially pronounced in cancers of unknown primary (CUP), which comprise 3–5% of diagnoses and frequently lack effective therapeutic strategies due to their elusive origins^41^. Despite advances in genomic profiling and imaging technologies, reliable molecular strategies for pinpointing the origin of CUP remain scarce. We hypothesize that primary tumors retain features of their tissue of origin and that metastatic cancers preserve the regulatory signature of the primary tumor. Although primary tumors are not included in our dataset, the broad coverage of normal tissues may enable inference of metastatic origins.

To test this, we leveraged our nascent transcriptome dataset, which includes a diverse panel of metastatic tumors (n=23) and normal tissues from multiple organ systems (Fig. 6c, Supplementary Table 1). We compared the PRO-cap profiles of divergent elements in metastatic cancer samples to those of normal tissues from the corresponding primary, metastatic, and other tissue types. Pairwise comparison revealed that most metastatic tumors more closely resembled their tissue of origin, supporting the hypothesis that metastatic cancers preserve the molecular signatures of their primary sites (Fig. 6c, Supplementary Fig. 10a). Additionally, correlations with metastatic sites were higher than with other tissues, suggesting an adaptation to the metastatic microenvironment.

To evaluate the predictive utility of these patterns, we trained a linear support vector classifier using divergent distal elements from normal samples spanning 15 tissue types. Remarkably, although trained exclusively on non-neoplastic tissues, the model achieved an overall accuracy of 87.0% for the top-1 prediction and 95.7% for the top-3 predictions in identifying the primary origin of metastatic tumors (Fig. 6d, Supplementary Fig. 10b). Likewise, divergent proximal elements yielded comparable prediction accuracy (Supplementary Fig. 10b). These findings demonstrate that nascent transcriptome profiling may offer a promising molecular strategy for resolving diagnostically ambiguous cases and guiding more precise therapeutic interventions.

### Site-specific metastatic adaptation via enhancer reprogramming

**Extended Data Fig. 10 |:**
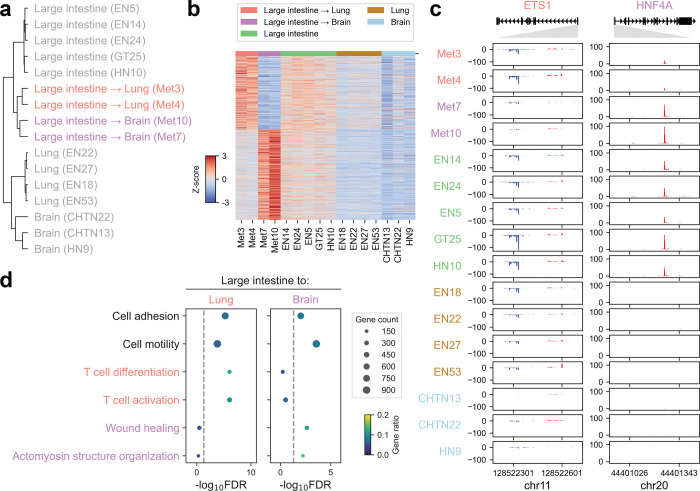
Site-specific regulatory rewiring in lung and brain metastases of colorectal cancer **(a)** Hierarchical clustering of divergent distal TREs in metastatic biopsies from patients with colorectal cancer and related non-neoplastic tissues. **(b)** Heatmap of differentially expressed divergent distal TREs between lung and brain metastases. Z-scores of PRO-cap signals were calculated across metastatic samples and related non-neoplastic tissues. **(c)** Genome browser tracks of PRO-cap signals at the promoter regions of *ETS1* and *HNF4A* in lung and brain metastases from colorectal cancer alongside related non-neoplastic tissues. **(d)** Pathway enrichment analysis of genes linked to differentially expressed TREs in lung or brain metastases from colorectal cancer, respectively. Gene ratio represents the fraction of genes in each pathway that overlap with the input gene set.

The preferential transcriptional similarity between metastatic tumors and their corresponding secondary sites, relative to unrelated tissues, suggests a high degree of plasticity (Fig. 6c). This prompted us to examine whether specific enhancer programs underlie metastatic dissemination and organ-specific adaptation. We first performed hierarchical clustering of divergent distal TREs across metastatic biopsies from 4 patients with colorectal cancer (CRC) and related non-neoplastic tissues (i.e., large intestine, lung, and brain) (Extended Data Fig. 10a). This analysis uncovered two major patterns: (i) both brain and lung metastatic lesions retained stronger regulatory similarity to their tissue of origin (i.e., large intestine) than to distant sites, which aligns with our prior observation (Fig. 6c); and (ii) within the metastatic cohort, tumors segregated by their destination site.

To interrogate the regulatory mechanisms underlying organ-specific dissemination and adaptation, we identified differentially expressed TREs between lung and brain metastases (Extended Data Fig. 10b). Motif enrichment analysis of enhancers upregulated in lung metastases revealed a marked overrepresentation of ETS1, RUNX1, and their composite motifs (Fig. 6e, Supplementary Table 3), consistent with their established roles in metastatic progression. Specifically, stromal ETS1 expression in primary CRC is associated with increased risk of dissemination to the lungs in patients^42^. Similarly, previous studies have demonstrated that RUNX1 promotes lung colonization of metastatic CRC in murine models, with elevated expression correlating with poor clinical outcomes^43^. Thus, our results extend these prior observations by suggesting that the coordinated activity of these TFs may shape the enhancer landscape in lung metastases of CRC. In contrast, enhancers upregulated in brain metastases showed significant enrichment for HNF4A and PPARγ motifs (Fig. 6e, Supplementary Table 3). Although PPARγ signaling and HNF4A expression have been linked to brain metastatic progression in melanoma^44,45^, their specific roles in CRC brain dissemination has yet to be elucidated. This selective enrichment of HNF4A and PPARγ motifs in brain metastasis-specific enhancers thus uncovers a previously unrecognized regulatory axis and nominates these factors as compelling candidates for mediating colonization of the brain microenvironment.

To assess the potential driver roles of ETS1 and HNF4A, we first examined their differential expression in metastatic contexts. Both TFs exhibited elevated PRO-cap signals at their promoters, consistent with transcriptional activation (Extended Data Fig. 10c). These factors are also expressed in non-neoplastic large intestine tissues (Extended Data Fig. 10c), supporting the notion that metastases may co-opt pre-existing regulatory networks during dissemination and adaptation. To further contextualize the regulatory programs associated with metastatic homing, we performed pathway enrichment analysis of genes linked to enhancers either upregulated in lung or brain metastases. This analysis revealed shared pathways of cell adhesion, motility, and vascular development, highlighting core mechanisms of invasive spread and niche formation in CRC metastasis (Extended Data Fig. 10d). We also uncovered distinct, site-specific programs: lung metastases were enriched for pathways related to immune modulation, whereas brain metastases showed enrichment for pathways central to microenvironmental adaptation and blood-brain barrier (BBB) remodeling. Thus, these findings position ETS1 and HNF4A as key mediators of enhancer reprogramming, highlighting how metastatic tumors hijack lineage-primed regulatory circuits while reshaping their enhancer landscapes to colonize distinct organs. Together, these results underscore the importance of context-specific regulatory factors in driving the tissue tropism of metastatic cancer.

## Discussion

Nascent transcription initiation precisely marks active gene promoters and enhancers^1–4^. Using PRO-cap across diverse developmental, physiological, and pathological contexts, we mapped initiation at base-pair resolution and identified hundreds of thousands of active TREs. This comprehensive atlas reveals the breadth of regulatory elements that shape cellular and tissue identity in health and disease.

These maps provide the foundation to uncover how nascent transcription operates as a fundamental layer of gene regulation, complementing and extending the information encoded within the epigenome. In this study, we examined its interplay with opening chromatin to regulatory machinery. By systematically comparing chromatin accessibility with or without nascent transcription in pluripotent stem cells, we identified both shared and distinct motifs underlying these two layers. These results underscore the importance of integrating multiple regulatory layers for a comprehensive understanding of gene control.

Beyond mapping regulatory element activity, our PRO-cap-based approach captures transcription initiation and pause site architecture at base-pair resolution, revealing features beyond the reach of other methods and enabling mechanistic dissection of early transcriptional events. This high-resolution perspective deepens our understanding of the distinct steps governing gene regulation and how they vary across regulatory element classes and cell states.

Furthermore, integration with functional genomics enables the prioritization of likely causal noncoding variants and the characterization of their tissue-specific effects, linking regulatory activity to potential phenotypic outcomes. In cancer, TRE maps reveal lineage-specific transcriptional programs, trace the origins of metastases, and uncover dynamic circuits that drive tumor dissemination and adaptation. These findings provide a mechanistic framework for understanding how regulatory elements contribute to disease progression.

Together with other large-scale efforts, our data provide complementary insights into the architecture of the regulatory genome and help lay the groundwork for a unified model of transcriptional regulation across diverse biological contexts. Future integration of TRE maps with gene expression measurements, such as RNA-seq and PRO-seq, will further refine our understanding of gene regulatory output.

Collectively, this detailed catalog of active TREs provides an essential resource for dissecting gene regulation and advancing applications from basic molecular biology to clinical genomics.

## Methods

### Sample collection and PRO-cap library preparation:

Snap frozen human tissue samples from cadaveric or surgical biopsies were obtained from a range of sources, including ENCODE, and GTEx, as part of the ENCODE consortium’s coordination Phase 4 efforts, and the National Cancer Institute’s Cooperative Human Tissue Network (CHTN). Solid tissues were pulverized on dry ice using a mortar and pestle. Peripheral blood mononuclear cells (PBMCs) were isolated using Lymphoprep, and specific immune cell populations were purified from LeukoPaks via fluorescence-activated cell sorting (FACS) using positive or negative selection based on surface marker expression. Cells were either cultured in-house or obtained as snap-frozen dry pellets. PRO-cap libraries were prepared as previously described, with modifications to streamline the protocol, reducing the experimental time from 3–4 days to approximately 14 hours, while enabling the use of limited tissue input and low cell numbers from diverse sample types, without compromising the assay’s sensitivity or specificity. Briefly, permeabilized cells or pulverized tissues underwent nuclear run-on reactions to capture nascent RNA. Total RNA was then isolated and subjected to two rounds of custom adaptor ligation and reverse transcription. Between adaptor ligations, 5′ cap selection was performed through a series of enzymatic reactions to enrich for capped nascent transcripts. RNA was washed, followed by phenol:chloroform extraction and ethanol precipitation at each step, all under RNase-free conditions. Following PCR amplification and library clean-up, sequencing was performed on an Illumina NovaSeq platform. Likewise, we generated PRO-cap libraries from two replicates, each of 10 million HCT116 cells genetically modified using CRISPR targeting H. sapiens BRD4, CDK7, CTCF, MED14, POLR2A, RAD21, SMARCA5, SUPT16H and O. sativa LOC4335696 (OsTIR1 auxin receptor for the auxin-inducible degron system), before and after 6 hours of treatment with 1 μM 5-Phenyl-1H-indole-3-acetic acid. Details of all biosample and library information can be found in Supplementary Table 1.

### Data preprocessing:

This dataset was managed and analyzed using the Resource Management System (https://github.com/aldenleung/rmsp/). Raw reads were preprocessed with fastp^46^ (v0.23.4) for adapter trimming and unique molecular index (UMI) processing, retaining only reads ≥18 bp for downstream analyses. Processed reads were aligned to the human reference genome hg38 (GCA_000001405.15) and ribosomal DNA (U13369.1) using STAR^47^ (v2.7.11a). Uniquely mapped reads were filtered with samtools^48^ (v1.18) and deduplicated using umi_tools (v1.1.5). FASTQ files of PRO-cap data from the NELF-C degron lines (NELFC_U, NELFC_T) were obtained from GSE144786 and reprocessed with the same pipeline, except that PCR deduplication was omitted due to the lack of UMI information. The resulting PCR-free, uniquely mapped reads were converted to bigwig format with biodatatools (v0.0.7) and used for peak calling with PINTS^5^ (v1.1.10). For replicates showing good correlation, bigwig files were merged before peak calling. Identified elements were classified as proximal (within ±500 bp) or distal (outside ±500 bp) categories based on their distances to the TSSs of all genes annotated by GENCODE^49^ (v37). Sequencing details are provided in Supplementary Table 1.

The gene body ratio was calculated as a quality control metric to assess 5′ enrichment in capped RNA sequencing experiments. Analyses were restricted to highly expressed genes, defined as the top 10% ranked by PRO-cap signal within the proximal region (1 kb upstream to 100 bp downstream of the gene TSS). For each gene, the ratio was computed as the normalized signal in the gene body (500 bp downstream of the TSS to 500 bp upstream of the transcription termination site) divided by the sum of the normalized signals in both the gene body and TSS (0–500 bp downstream of the gene TSS) regions, with normalization to the total length of each region. This measure reflects the extent to which capped reads are concentrated at TSS regions in high-quality data. One sample with a gene body ratio ≥ 0.025 was excluded from downstream analyses.

### Quantification of transcription initiation.

We counted reads whose 5’ ends aligned within the boundaries of individual elements in each sample. The resulting count matrix was normalized using the median of ratios method in DESeq2^50^ to account for differences in library size and RNA compositional bias. We then applied variance stabilizing transformation to mitigate the mean-variance dependency, as recommended by DESeq2, for downstream analyses including PCA and clustering.

### Calculate specificity scores.

The specificity scores were calculated following an approach similar to that previously described^2,51^. All samples from Fig. 2a were included, and $q_{e,t}$ was defined as the average normalized expression of an element e across samples of the same tissue type t. We first converted $q_{e,t}$ to probabilities $p_{t|e}=q_{e,t}/\sum_{t}q_{e,t}$. The entropy of a given element was then computed as $H_{e}=-\sum_{t}p_{t|e}{log}_{2}\left(p_{t|e}\right)$. To constrain the specificity score within the range of 0 to 1 (where 0 indicates ubiquitous expression and 1 represents exclusively specific expression), we defined $S_{e}=1-H_{e}/{log}_{2}N$, with N denoting the number of tissue types.

### Get tissue-specific and non-tissue-specific elements.

We computed a *t*-statistic for the specific expression of each divergent distal or proximal element in tissue types with at least three samples, as highlighted in Fig. 2a, following the procedure described in Finucane *et al*^52^. Briefly, a design matrix X was constructed with an indicator column denoting sample membership in the given tissue type (1 for yes, −1 for no) and an intercept term. The outcome Y was the normalized expression level of a given element across samples. We fit the model via ordinary least squares and calculated the *t*-statistic for the membership variable. The top 5% of elements from the union set, ranked by their *t*-statistic, were defined as the specifically expressed set for each tissue type. Non-tissue-specific elements were defined by the following criteria: (1) distal elements with a specificity score < 0.1 and proximal elements with a specificity score < 0.02 were included, resulting in a number of elements similar to that of the tissue-specific set for each tissue type; (2) elements belonging to any tissue-specific set were excluded.

### Time series clustering of dynamically transcribed TREs during pancreatic lineage differentiation.

We took the union set of PRO-cap elements across four timepoints (ESC, ESC-derived endodermal cells, pancreatic progenitor cells, and insulin-producing beta-like cells) to generate a count matrix. DESeq2 was used to identify elements with differential PRO-cap signals between any pair of timepoints, with an FDR threshold of 0.01. Dirichlet process-Gaussian process (DP-GP) time-series clustering^53^ was applied to categorize the dynamically transcribed elements into distinct trajectories over time. We followed a similar two-stage strategy described in Kim *et al*^54^, except that replicate reproducibility was not required due to the absence of biological replicates in our dataset. First, we subsampled the expression data (n=5,000 for computational efficiency, as the algorithm was originally designed for thousands of genes) and applied the DP-GP algorithm with default parameters to generate the initial set of time series clusters. The cluster set was further filtered by excluding (1) clusters with fewer than 2% of dynamically transcribed elements and (2) non-dynamic trajectories whose multivariate Gaussian process did not reject the null hypothesis of no change over time. In the second stage, each dynamically transcribed element was assigned to a cluster if it fell within the 95% multivariate confidence interval of the trajectory. If multiple clusters matched, the element was assigned to the one with the smallest Euclidean distance from the mean trajectory. Elements without a matching cluster were discarded. To validate the biological relevance of these clusters, we assessed whether their target genes were enriched for expected pathways. The final set of elements was linked to the nearest target genes expressed (>1 TPM) at any point during the time course. Pathway enrichment analysis^55^ for each cluster was performed using the Canonical Pathways gene sets derived from the Reactome pathway database^56^ (c2.cp.reactome.v2023.2) in the Human Molecular Signatures Database (MSigDB)^57^.

### Partition heritability using S-LDSC.

A total of 176 GWAS summary statistics were obtained from the curated collection of the Alkes Price lab (https://console.cloud.google.com/storage/browser/broad-alkesgroup-public-requester-pays/LDSCORE/all_sumstats/). To capture both tissue-specific and non-tissue-specific effects, we used the elements defined in the “Get tissue-specific and non-tissue-specific elements” section. For analyses of immune-related phenotypes across blood cell types, all divergent distal elements identified in each blood sample were included. We then further extended these elements by ±1kb from the peak center to capture putatively functional variants in the flanking regions. To compute annotation-specific LD scores with a 1 cM window, we used the 1000 Genome Phase 3 data of European ancestry as the reference panel. To identify critical tissue types for a given phenotype, we ran S-LDSC with the “--h2-cts” flag to partition the heritability using our TRE annotations, conditional on the baseline model v1.2 as recommended by the LDSC developers (“LDSCORE/readme_baseline_versions”). The reference panel and baseline model were downloaded from LDSCORE/GRCh38/.

### Luciferase assay for evaluating GWAS variant effects on enhancer activity.

In the section “Tissue-specific effects of disease- and trait-associated variants”, we assessed the effect of the UC-associated variant rs6426833 on enhancer activity using a dual-luciferase reporter assay. Elements (chr1:19844867–19845867) containing either the reference or alternative alleles were cloned following previously described protocols^3^. Primers were designed using our in-house web tool^58^, incorporating attB1′ (forward) and attB2′ (reverse) 5′ overhangs (Supplementary Table 4). K562 genomic DNA (E493; Promega Corp.) served as the template for PCR amplification using Phusion High-Fidelity (M0530; New England Biolabs) and PrimeSTAR GXL (R050A; Takara Bio Inc.) DNA polymerases. Amplicons were inserted into pDONR223 via Gateway BP cloning, sequence verified, propagated in spectinomycin-supplemented lysogeny broth (LB), and purified with E.Z.N.A. Plasmid DNA Mini Kit II (D6904; Omega Bio-tek, Inc.). Verified elements were transferred to pDEST-hSTARR-luc-pMYC via Gateway LR cloning, propagated in ampicillin-supplemented LB, and extracted using E.Z.N.A. Endo-Free Plasmid DNA Midi Kit (D6915; Omega Bio-tek, Inc.).

HCT116 (CCL-247; ATCC) cells were cultured in McCoy’s 5A Medium (30–2007; ATCC) supplemented with 10% FBS (30–2020; ATCC) at 37°C with 5% CO_2_. Caco-2 (HTB-37; ATCC) cells were cultured in EMEM (30–2003; ATCC) supplemented with 20% FBS (30–2020; ATCC) under the same conditions. Vectors were transfected into HCT116 and Caco-2 cells using Lipofectamine 3000 (L3000001; Invitrogen), with 0.5 × 10^6^ cells receiving 1 μg of pDEST-hSTARR-luc-pMYC and 10 ng of pGL4.75 (E6931; Promega Corp.). After 24h of incubation, cells were dissociated with 0.25% Trypsin-EDTA (25200056; Gibco) for the dual-luciferase reporter assay. Luminescence was measured using the Dual-Glo Luciferase Assay System (E2920; Promega Corp.) on an Infinite M1000 microplate reader (30034301; Tecan Group Ltd.) following the manufacturer’s instructions. Cells transfected with only pDEST-hSTARR-luc-pMYC or only pGL4.75 were used as background controls for firefly and Renilla luciferase activities, respectively.

### eQTL enrichment using TORUS.

eQTL summary statistics were obtained from 28 GTEx v8 tissue types matching those in our PRO-cap dataset (https://console.cloud.google.com/storage/browser/gtex-resources/GTEx_Analysis_v8_QTLs/GTEx_Analysis_v8_EUR_eQTL_all_associations). We applied TORUS to estimate functional enrichment for each annotation described in the “Partition heritability using S-LDSC” section. The tool outputs 95% confidence intervals for the log enrichment parameters, from which p-values are derived under the assumption of asymptotic normality^59^.

### Fine-mapped GWAS and eQTL variants.

Both fine-mapped eQTL variants for GTEx tissues and GWAS variants for UK Biobank traits were obtained from https://www.finucanelab.org/data/. In this dataset, fine-mapping was performed using FINEMAP^60^ and SUSIE^61^. Variants included in the primary release were selected as follows: for GWAS, those within the 95% CS or with PIP > 0.001; for eQTL, those within the 95% CS or with PIP > 0.0001. We analyzed data from both fine-mapping approaches and observed consistent trends. For clarity and conciseness, only the FINEMAP results are presented in the manuscript.

### Motifs contributing to transcription versus accessibility.

The ChromBPNet model trained on ESC ATAC-seq data (GSE267154) and related processed outputs, including contribution scores and TF-MoDISco results, were downloaded from Synapse (syn59449898). The ProCapNet model was trained on ESC PRO-cap data with default parameters. Contribution scores were calculated using DeepSHAP^62^ following the procedure described by Cochran *et al*^10^. TF-MoDISco was applied to identify recurring sequence patterns, and TOMTOM^63^ was used to annotate the top-matching TF motifs from the JASPAR database^64^. For both models, we retained motif patterns supported by at least 200 seqlets from TF-MoDISco and filtered out motifs that were either simple GC or AT repeats or could not be reliably matched to known motifs.

To compare motif content across categories (motifs contributing to accessibility in ATAC-seq peaks with and without overlapping PRO-cap peaks, and motifs contributing to transcription in PRO-cap peaks), we employed the Fi-NeMo tool (https://github.com/austintwang/finemo_gpu; MIT License) to identify motif instances in ATAC-seq and PRO-cap peaks based on contribution scores for the count task from the respective models, with default parameters. Fi-NeMo was run separately using the unique TF-MoDISco contribution weight matrices (CWMs) originally identified for each pattern from both the ChromBPNet and ProCapNet models. For downstream analyses, motif instances attributed to a given motif were those Fi-NeMo instances mapped to the original TF-MoDISco patterns identified by the corresponding model. For motifs lacking an original TF-MoDISco pattern in one model, the Fi-NeMo instances mapped to the CWMs from the other model were used. This approach ensured that motif instances were assigned based on the exact motif patterns learned by each model whenever available, while allowing for matches to motifs not detected by TF-MoDISco in one model but present in the other.

To directly evaluate contributions to accessibility and transcription for individual motif instances, we recalculated contribution scores for the count task of the ChromBPNet model on PRO-cap peaks using the same input regions as ProCapNet. Motif hits were then called from these recalculated scores using Fi-NeMo. For motif instances located within PRO-cap peaks overlapping ATAC-seq peaks, we summed the contribution scores from both ChromBPNet and ProCapNet models and computed the Pearson correlation across instances for each motif type.

### Predicting variant effects on nascent transcription using ProCapNet models.

To evaluate the context-specific regulatory effects of fine-mapped variants, we first trained ProCapNet models on PRO-cap data from different tissue types, using default parameters except with “in_window” set to 1,000 and “out_window” set to 500. We identified motif patterns for both count and profile tasks of each model using TF-MoDISco and called hits with Fi-NeMo, following the same procedure described in the “Motifs contributing to transcription versus accessibility” section. To score variant effects across diverse contexts (Fig. 4), we applied these models to a 1-kb input sequence centered at candidate elements containing either the reference or alternate allele. Variant effects were quantified as the log_2_ fold change in total counts between the alternate and reference alleles.

### Principal component analysis on embeddings of ProCapNet models.

Model embeddings were defined as described in Cochran *et al*^10^, i.e., the output of the global average pooling layer within the count task head. We generated embeddings for all PRO-cap peaks from each sample using the corresponding model and performed PCA on the resulting embeddings. Different TRE categories (distal vs. proximal, unidirectional vs. divergent, and P-P vs. B-B) were then projected into the PCA space.

### Peak shape classification.

We first combined reads across samples of the same tissue type. Only divergent elements that met the following criteria were included: (1) at least 50 reads on each strand; (2) called by PINTS in at least one sample. To calculate the shape index (SI) for a given strand, we applied the formula described in Hoskins *et al*.^30^, $SI=2+\sum_{i}^{L}p_{i}{log}_{2}p_{i}$, where p is the probability of observing a read at base position i within the element, and L is the set of base positions that have at least one read. Peaks on a given strand with an SI > −1.5 were classified as “peaked” (P); all others were classified as “broad” (B).

### Promoter-enhancer (P-E) connections.

We obtained P-E connections (E2G models) for each tissue type from the ENCODE portal. Distal PRO-cap elements were mapped to tested elements in the E2G “element gene links” files (1-bp overlap; if multiple elements overlap, the one with the largest overlap is selected; elements with zero overlap are discarded). Proximal PRO-cap elements were mapped to tested elements in the E2G files with the “isSelfPromoter” column set to True and assigned the corresponding gene symbol. The number of target genes for a given distal element and the number of enhancers regulating a given gene are calculated based on the “thresholded element gene links” files.

### Motif enrichment analysis.

For the leukemia and lymphoma panel, we ran HOMER^65^ for *de novo* motif discovery in each TRE module, using the union set of divergent distal elements from this panel as the background. The top motifs identified in each module were compiled into a custom set of known motifs to calculate enrichment in all modules. For the T1D and metastatic panels, we performed motif enrichment using HOMER with the default motif database.

### CRISPRi screening of TRE modules at the *MYC* locus.

B-ALL cell lines REH, SEM, and NALM-6 were co-transduced with virus generated from lentivectors TRE-KRAB(ZNF10)-dCas9-IRESGFP (RRID: Addgene_85556) and EF1a_TetOn3G (Clontech), and were serially flow sorted to derive populations that were GFP-negative in the absence of doxycycline and GFP-positive after induction with 500 ng/ml doxycycline. For SEM, a polyclonal population of inducible KRAB-dCas9+ cells was used, while for REH and NALM-6, single cell clones were derived by limiting dilution and clones with uniform transgene inducibility were validated and used for screening. Gene knockdown efficiency was validated by qRT-PCR after transduction with sgRNAs targeting the ENO1 promoter or non-genome-targeting control in the presence and absence of doxycycline. Inducible KRAB-dCas9+ cell populations were validated for cell line identity by STR profiling prior to the CRISPRi screen. CRISPRi screens to determine fitness effects of *MYC* locus TRE modules were performed in B-ALL cell lines via the same sgRNA library and protocol previously described for K562^66^ and KARPAS-422^39^. CRISPRi screening results from SU-DHL-5 was previously published^39^, and used a different sgRNA library targeting nucleosome-free regions of *MYC* locus elements with significant acetylation in mature B-cell lymphoma cell lines.

### Pairwise comparison of metastatic cancer samples and normal tissue samples.

We included 93 normal tissues samples from 15 tissue types (highlighted in Fig. 2a), and 23 metastatic cancer samples. PCA was performed using normalized PRO-cap signals at divergent distal and proximal elements identified in these samples. Principal components explaining 90% of the variance were extracted to calculate Pearson correlations between metastatic cancer samples and normal tissue samples from the corresponding primary site, metastatic site, and other sites.

### Predicting the primary site of metastatic tumor samples.

The training dataset consists of the 93 normal samples from 15 tissue types (Fig. 2a). Tissue-specific elements, obtained as described in the “Get tissue-specific and non-tissue-specific elements” section, were combined into a union set of features, excluding those found in multiple tissues. A linear support vector classifier was trained on this feature set with default hyperparameters and evaluated on metastatic samples with known primary sites.

### Schematics.

All schematics in Figs. 1, 2, and 6 were created using BioRender, with the appropriate publication licenses.

## Extended Data

## Supplementary Material

Supplement 1

Supplement 2

Supplement 3

Supplement 4

Supplement 5

## Data Availability

The PRO-cap datasets generated in this study will be deposited in the ENCODE portal and publicly available upon publication.

## Code availability

All code used in this study has been deposited at https://github.com/haiyuan-yu-lab/TRE_landscape.
