# Supplementary material for: The nascent transcriptome delineates the regulatory landscape in human health and disease
Source: bioRxiv. 2026 Jun 9:2025.09.24.676871. Originally published 2025 Sep 26. Preprint. [Version 2] doi: 10.1101/2025.09.24.676871 (PMC12485982; doi:10.1101/2025.09.24.676871)
Supplement: Supplement 5 [file NIHPP2025.09.24.676871v2-supplement-5.pdf]

Suppl. Fig. 1

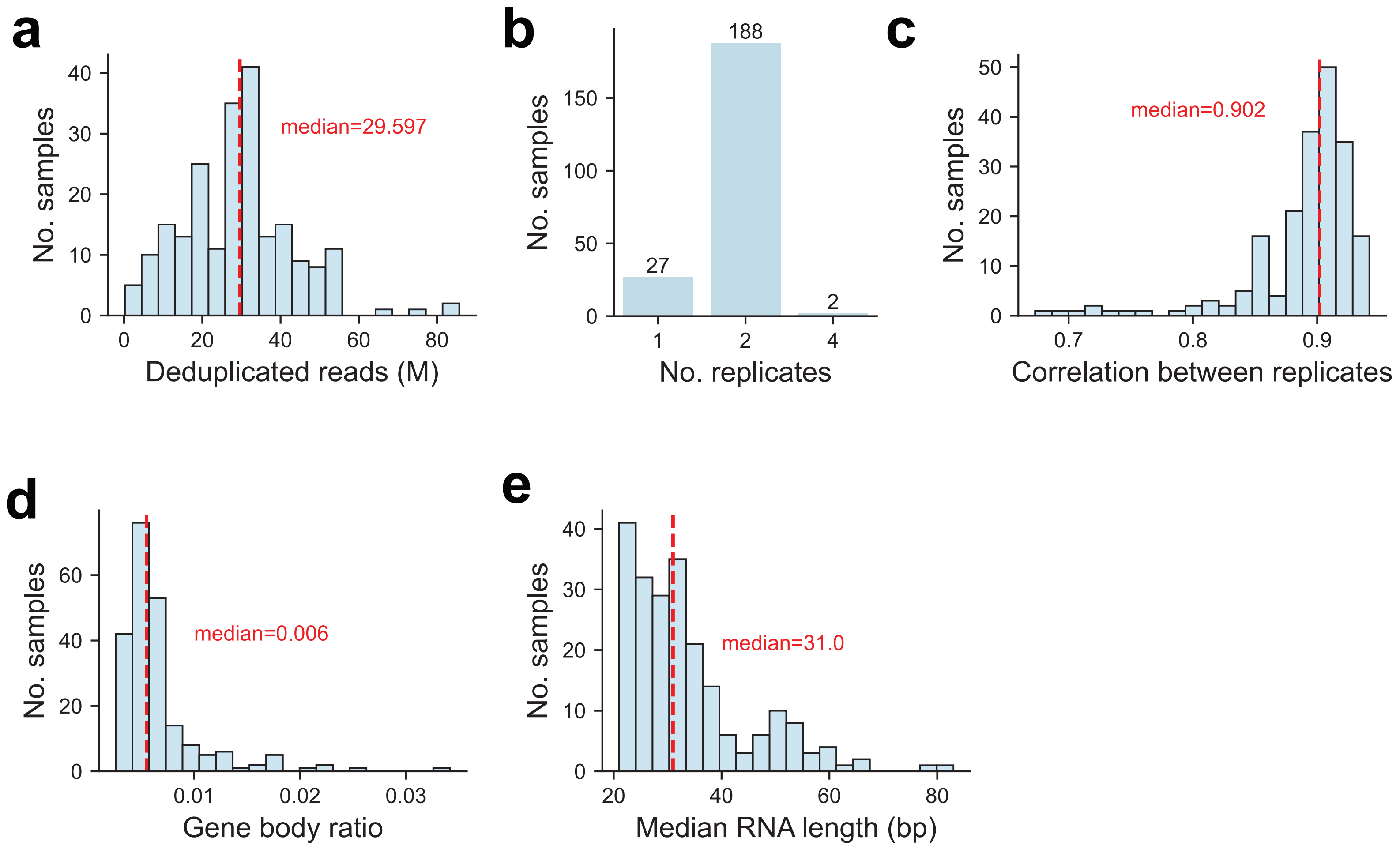
a
No. samples
median=29.597
Deduplicated reads (M)
b
No. samples
188
27
2
No. replicates
c
No. samples
median=0.902
Correlation between replicates
d
No. samples
median=0.006
Gene body ratio
e
No. samples
median=31.0
Median RNA length (bp)

bioRxiv preprint doi: https://doi.org/10.1101/2025.09.24.676871; this version posted June 9, 2026. The copyright holder for this preprint (which was not certified by peer review) is the author/funder, who has granted bioRxiv a license to display the preprint in perpetuity. It is made available under a CC-BY 4.0 International license.

Supplementary Figure 1 | Overview of PRO-cap data quality

(a) Histogram of deduplicated uniquely mapped reads (in millions) across samples. These represent high-quality reads after adapter trimming, quality filtering, alignment to the genome, and removal of PCR duplicates.

(b) Barplot showing the number of replicates of PRO-cap libraries generated per sample.

(c) Histogram of Pearson correlations between replicates (samples with ≥2 replicates), computed across genome-wide 5' PRO-cap signals.

(d) Histogram of gene body ratios across samples. Ratios represent length-normalized PRO-cap signal within the gene body relative to the sum of length-normalized TSS and gene body signals, for highly expressed genes.

(e) Histogram of median nascent RNA lengths (bp) captured by PRO-cap across samples. Lengths are derived from paired-end PRO-cap reads, reflecting the distance from the 5′ capped end to the 3′ pause site.

Suppl. Fig. 2

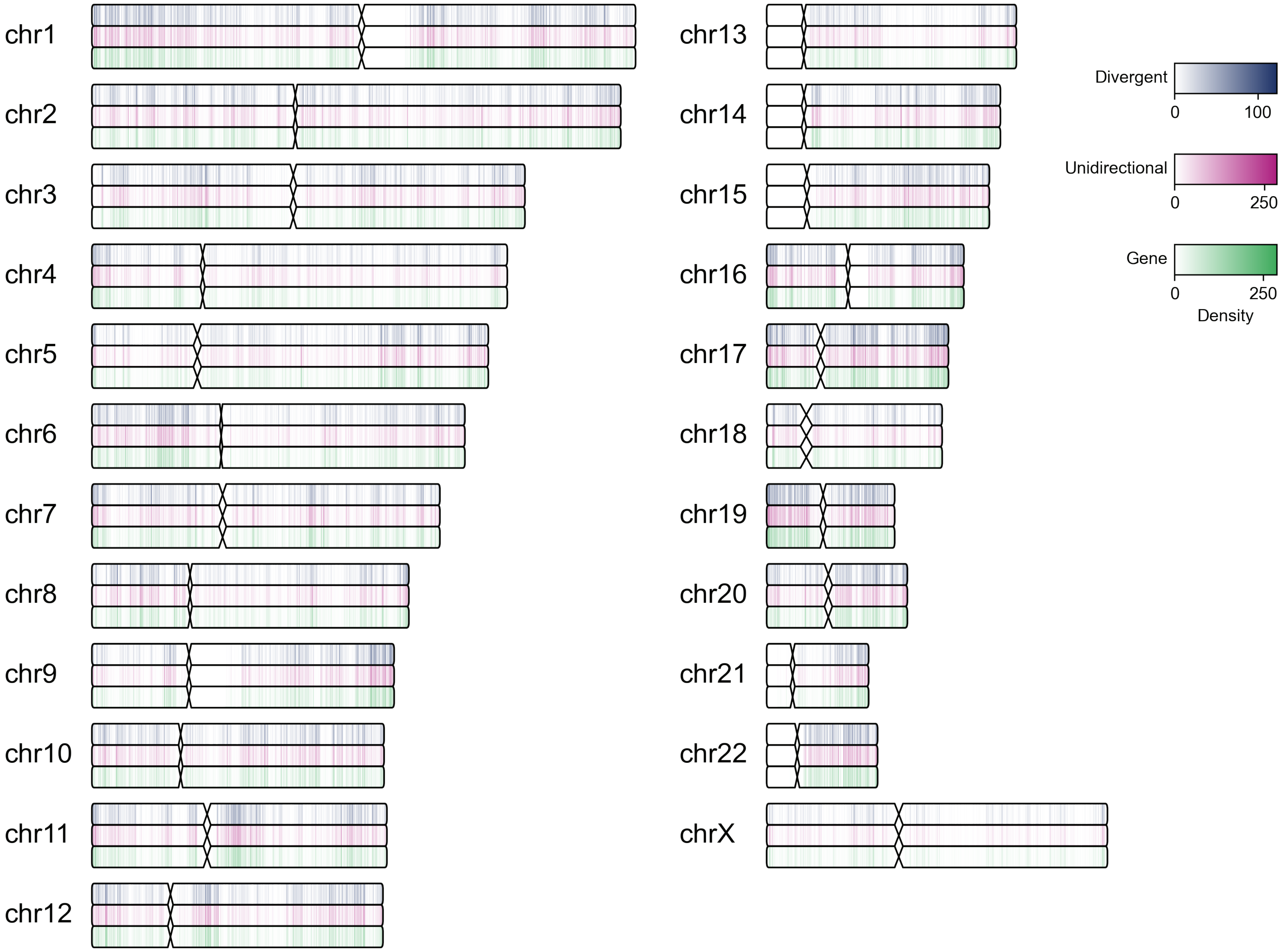
chr1
chr2
chr3
chr4
chr5
chr6
chr7
chr8
chr9
chr10
chr11
chr12
chr13
chr14
chr15
chr16
chr17
chr18
chr19
chr20
chr21
chr22
chrX
Divergent
0
100
Unidirectional
0
250
Gene
0
250
Density

bioRxiv preprint doi: https://doi.org/10.1101/2025.09.24.676871; this version posted June 9, 2026. The copyright holder for this preprint (which was not certified by peer review) is the author/funder, who has granted bioRxiv a license to display the preprint in perpetuity. It is made available under a CC-BY 4.0 International license.

Supplementary Figure 2 | Genomic distribution of PRO-cap-detected TREs

Genomic distribution of TREs and genes across the genome. For each chromosome, the top track shows divergent TREs (purple), the middle track shows unidirectional TREs (pink), and the bottom track shows genes (green). Densities represent log_{10}-transformed counts in 100 Mb bins and are displayed across the chromosomes.

Suppl. Fig. 3

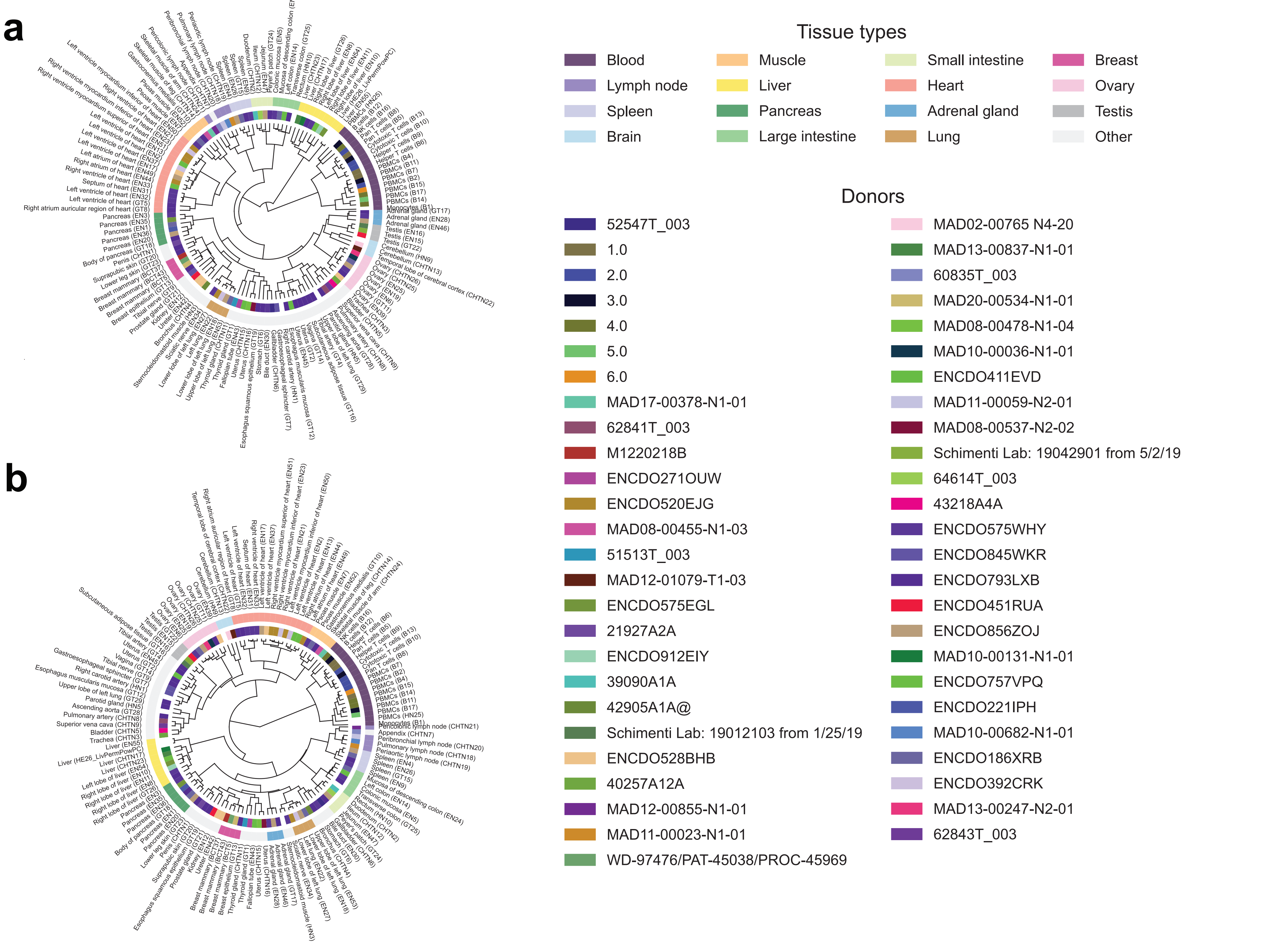
a
b
Tissue types
Blood
Muscle
Small intestine
Breast
Lymph node
Liver
Heart
Ovary
Spleen
Pancreas
Adrenal gland
Testis
Brain
Large intestine
Lung
Other
Donors
52547T_003
1.0
2.0
3.0
4.0
5.0
6.0
MAD17-00378-N1-01
62841T_003
M1220218B
ENCDO271OUW
ENCDO520EJG
MAD08-00455-N1-03
51513T_003
MAD12-01079-T1-03
ENCDO575EGL
21927A2A
ENCDO912EIY
39090A1A
42905A1A@
Schimenti Lab: 19012103 from 1/25/19
ENCDO528BHB
40257A12A
MAD12-00855-N1-01
MAD11-00023-N1-01
WD-97476/PAT-45038/PROC-45969
MAD02-00765 N4-20
MAD13-00837-N1-01
60835T_003
MAD20-00534-N1-01
MAD08-00478-N1-04
MAD10-00036-N1-01
ENCDO411EVD
MAD11-00059-N2-01
MAD08-00537-N2-02
Schimenti Lab: 19042901 from 5/2/19
64614T_003
43218A4A
ENCDO575WHY
ENCDO845WKR
ENCDO793LXB
ENCDO451RUA
ENCDO856ZOJ
MAD10-00131-N1-01
ENCDO757VPQ
ENCDO221IPH
MAD10-00682-N1-01
ENCDO186XRB
ENCDO392CRK
MAD13-00247-N2-01
62843T_003

bioRxiv preprint doi: https://doi.org/10.1101/2025.09.24.676871; this version posted June 9, 2026. The copyright holder for this preprint (which was not certified by peer review) is the author/funder, who has granted bioRxiv a license to display the preprint in perpetuity. It is made available under a CC-BY 4.0 International license.

Supplementary Figure 3 | Hierarchical clustering of human tissue samples by divergent TREs

(a) Dendrogram showing hierarchical clustering of tissue samples based on normalized PRO-cap expression at divergent distal TREs. Sample names are labeled; subtrees primarily representing a single tissue type (≥3 samples) are highlighted. The outer circle indicates tissue type, and the inner circle indicates donor ID.

(b) Same as (a), but for divergent proximal TREs.

bioRxiv preprint doi: https://doi.org/10.1101/2025.09.24.676871; this version posted June 9, 2026. The copyright holder for this preprint (which was not certified by peer review) is the author/funder, who has granted bioRxiv a license to display the preprint in perpetuity. It is made available under a CC-BY 4.0 International license.

Suppl. Fig. 4

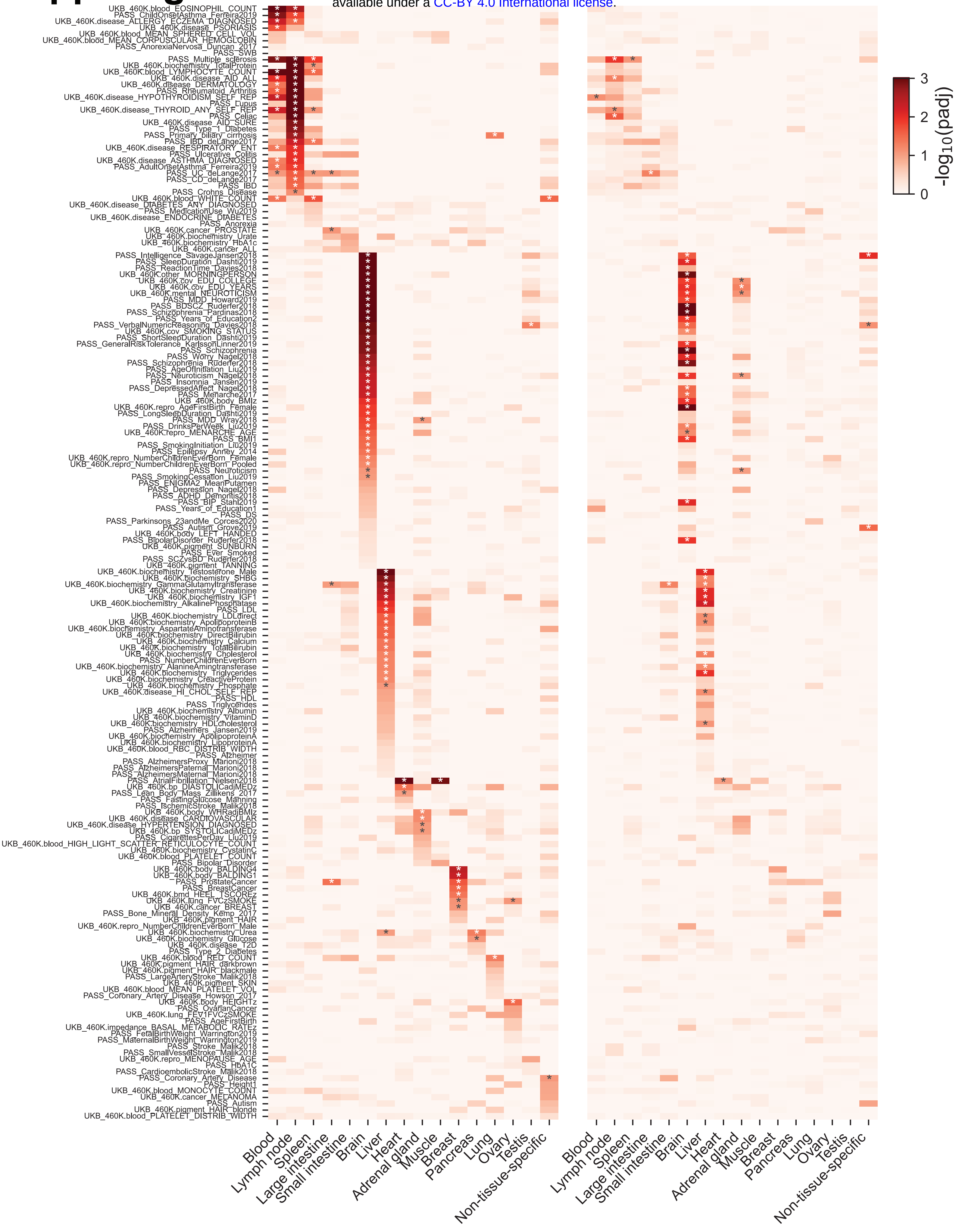

bioRxiv preprint doi: https://doi.org/10.1101/2025.09.24.676871; this version posted June 9, 2026. The copyright holder for this preprint (which was not certified by peer review) is the author/funder, who has granted bioRxiv a license to display the preprint in perpetuity. It is made available under a CC-BY 4.0 International license.

Supplementary Figure 4 | Tissue-specific effects of disease- and trait-associated variants

Heatmap showing the significance of enrichment in human diseases and complex traits from 176 studies across divergent distal (*left panel*; 7,943 tissue-specific elements per tissue type and 7,659 non-tissue-specific elements) and proximal (*right panel*; 2,540 tissue-specific elements per tissue type and 2,707 non-tissue-specific elements) TRE annotations. Asterisks (*) denote BH-adjusted p-values < 0.1. Only tissue types with at least 3 biological replicates were included.

bioRxiv preprint doi: https://doi.org/10.1101/2025.09.24.676871; this version posted June 9, 2026. The copyright holder for this preprint (which was not certified by peer review) is the author/funder, who has granted bioRxiv a license to display the preprint in perpetuity. It is made available under a CC-BY 4.0 International license.

Suppl. Fig. 5

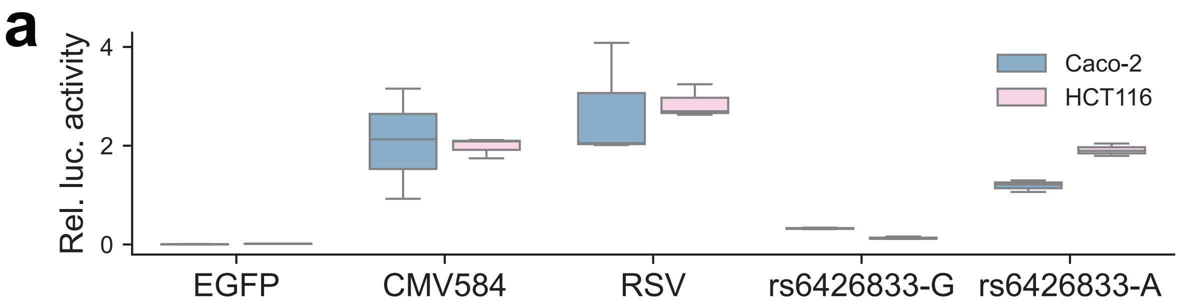

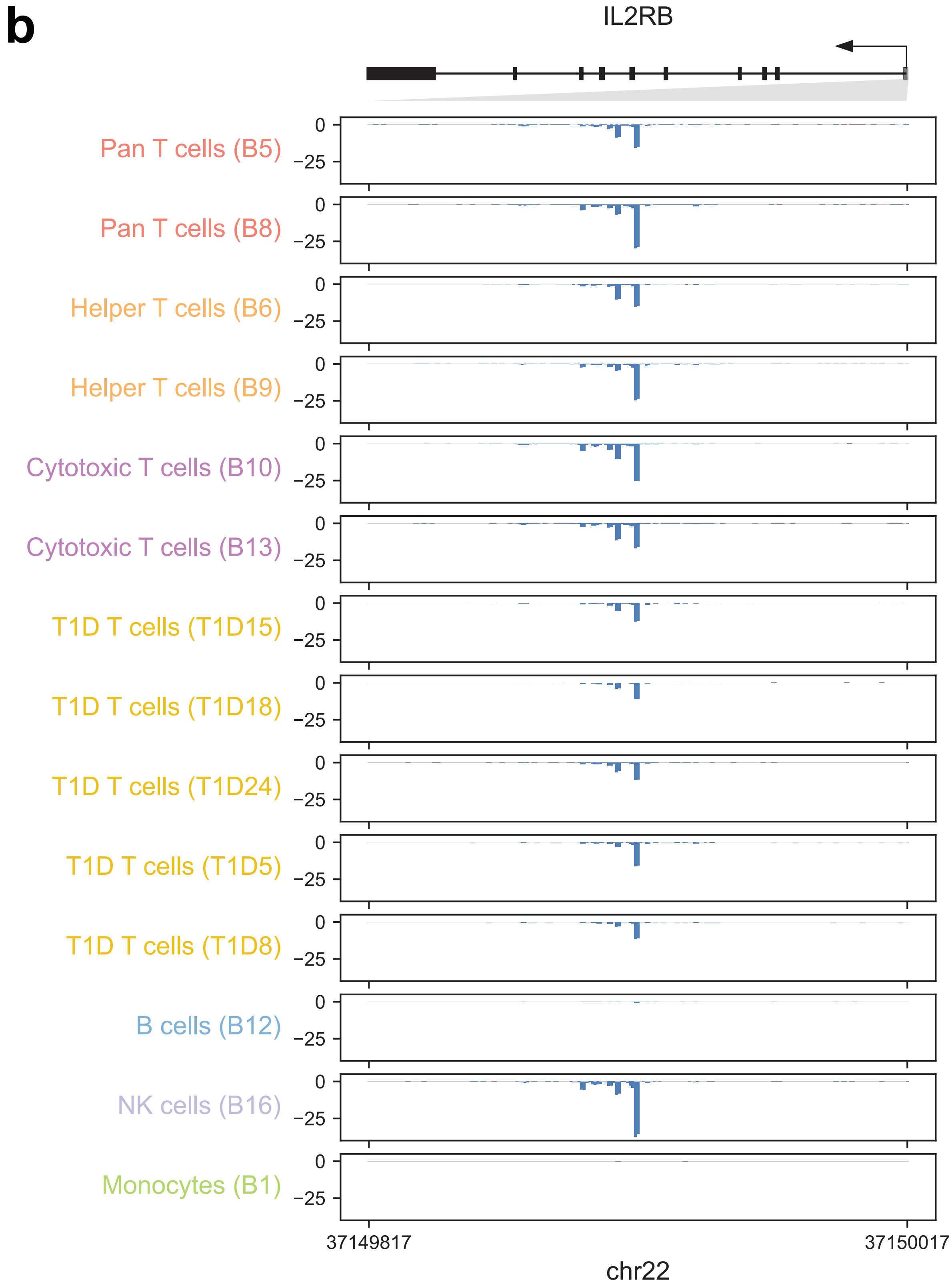

bioRxiv preprint doi: https://doi.org/10.1101/2025.09.24.676871; this version posted June 9, 2026. The copyright holder for this preprint (which was not certified by peer review) is the author/funder, who has granted bioRxiv a license to display the preprint in perpetuity. It is made available under a CC-BY 4.0 International license.

Supplementary Figure 5 | Disease-associated variants in TREs modulate regulatory activity

(a) Luciferase reporter assay showing enhancer activity of a negative control (EGFP), positive controls (CMV584, RSV), and two alleles (G vs. A) of rs6426833 in Caco-2 and HCT116 cells. Three biological replicates were included for each group.

(b) Browser shot of PRO-cap signal tracks (RPM-normalized) at the *IL2RB* promoter locus in T cells from individual T1D patients and in different immune cell types from each non-diseased donor. The *IL2RB* gene is depicted with an arrow indicating transcription orientation and black rectangles marking exons. The grey triangle highlights the region shown in the zoomed-in panels.

Suppl. Fig. 6

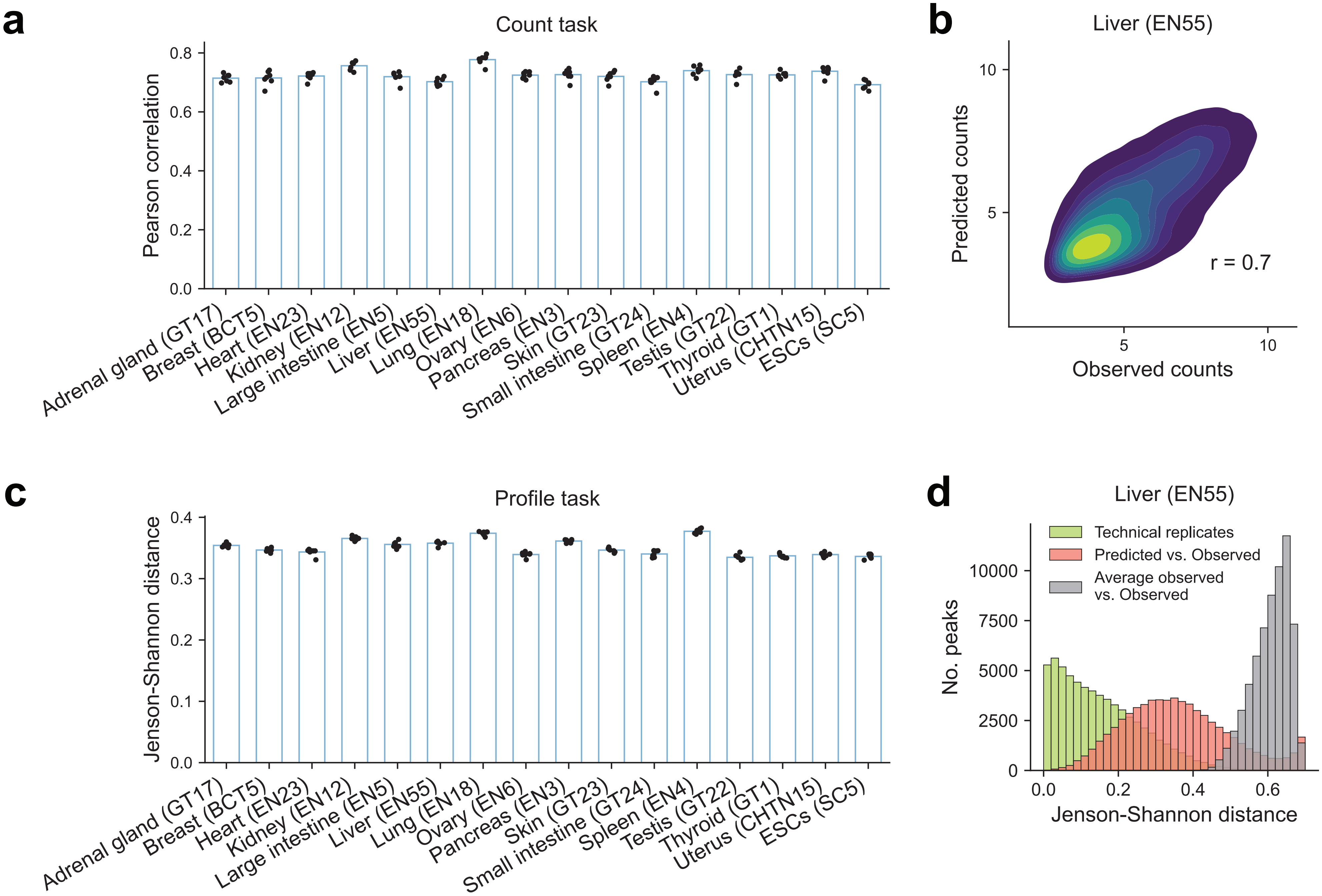
a
Count task
Pearson correlation
0.0
0.2
0.4
0.6
0.8
Adrenal gland (GT17)
Breast (BCT5)
Heart (EN23)
Kidney (EN12)
Large intestine (EN5)
Liver (EN55)
Lung (EN18)
Ovary (EN6)
Pancreas (EN3)
Skin (GT23)
Small intestine (GT24)
Spleen (EN4)
Testis (GT22)
Thyroid (GT1)
Uterus (CHTN15)
ESCs (SC5)
b
Liver (EN55)
Predicted counts
Observed counts
r = 0.7
5
10
c
Profile task
Jenson-Shannon distance
0.0
0.1
0.2
0.3
0.4
Adrenal gland (GT17)
Breast (BCT5)
Heart (EN23)
Kidney (EN12)
Large intestine (EN5)
Liver (EN55)
Lung (EN18)
Ovary (EN6)
Pancreas (EN3)
Skin (GT23)
Small intestine (GT24)
Spleen (EN4)
Testis (GT22)
Thyroid (GT1)
Uterus (CHTN15)
ESCs (SC5)
d
Liver (EN55)
Technical replicates
Predicted vs. Observed
Average observed vs. Observed
No. peaks
0
2500
5000
7500
10000
Jenson-Shannon distance
0.0
0.2
0.4
0.6

bioRxiv preprint doi: https://doi.org/10.1101/2025.09.24.676871; this version posted June 9, 2026. The copyright holder for this preprint (which was not certified by peer review) is the author/funder, who has granted bioRxiv a license to display the preprint in perpetuity. It is made available under a CC-BY 4.0 International license.

Supplementary Figure 6 | ProCapNet model performance across tissues

(a) Barplots showing the Pearson correlations between log-transformed observed and predicted total counts on held-out test chromosomes across 7-fold cross-validation for each model. Each datapoint represents one fold.

(b) Density plot of log-transformed observed and predicted counts at PRO-cap peaks from held-out test chromosomes across 7-fold cross-validation, based on the model trained on the liver sample (EN55).

(c) Same as (a), but showing the mean Jensen-Shannon distance between observed and predicted profiles for each model.

(d) Distribution of Jensen-Shannon distances between observed and predicted base-resolution profiles at PRO-cap peaks from held-out test chromosomes across 7-fold cross-validation (red), based on the model trained on the liver sample (EN55). For comparison, distances between observed profiles from two technical replicates of EN55 are shown in green (upper bound), and distances between observed profiles and profiles averaged over all peaks are shown in grey (baseline).

Suppl. Fig. 7

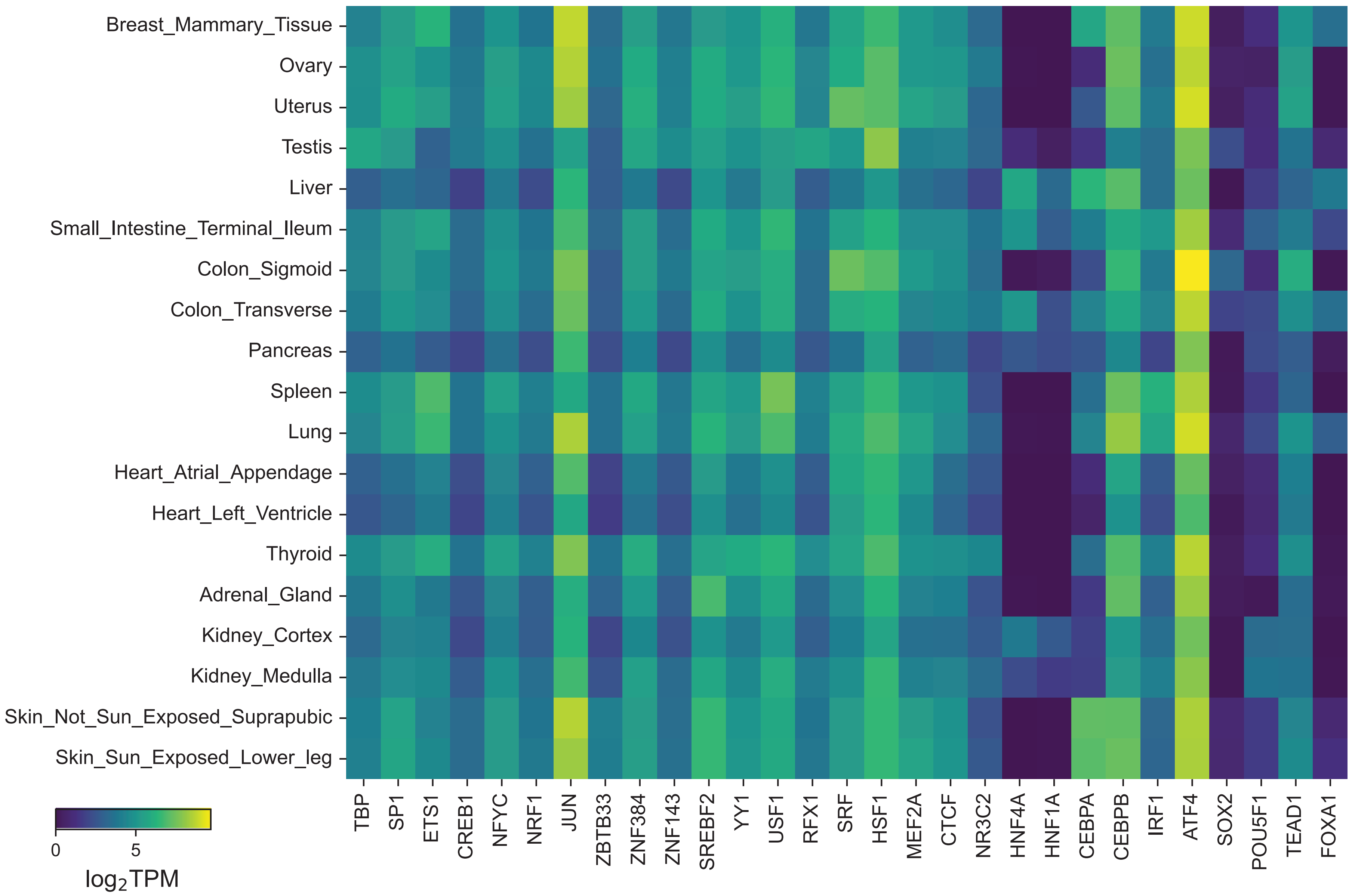
Breast_Mammary_Tissue
Ovary
Uterus
Testis
Liver
Small_Intestine_Terminal_Ileum
Colon_Sigmoid
Colon_Transverse
Pancreas
Spleen
Lung
Heart_Atrial_Appendage
Heart_Left_Ventricle
Thyroid
Adrenal_Gland
Kidney_Cortex
Kidney_Medulla
Skin_Not_Sun_Exposed_Suprapubic
Skin_Sun_Exposed_Lower_leg
TBP
SP1
ETS1
CREB1
NFYC
NRF1
JUN
ZBTB33
ZNF384
ZNF143
SREBF2
YY1
USF1
RFX1
SRF
HSF1
MEF2A
CTCF
NR3C2
HNF4A
HNF1A
CEBPA
CEBPB
IRF1
ATF4
SOX2
POU5F1
TEAD1
FOXA1
0
5
log_2TPM

bioRxiv preprint doi: https://doi.org/10.1101/2025.09.24.676871; this version posted June 9, 2026. The copyright holder for this preprint (which was not certified by peer review) is the author/funder, who has granted bioRxiv a license to display the preprint in perpetuity. It is made available under a CC-BY 4.0 International license.

Supplementary Figure 7 | Expression patterns of TFs corresponding to motifs contributing to transcription initiation at TREs across tissue types

Expression levels (median gene-level $\log_2$TPM per tissue from GTEx RNA-seq data) of representative TFs corresponding to motifs identified by ProCapNet as contributing to transcriptional strength (count task) and/or TSS positioning (profile task) across matched GTEx tissues.

bioRxiv preprint doi: https://doi.org/10.1101/2025.09.24.676871; this version posted June 9, 2026. The copyright holder for this preprint (which was not certified by peer review) is the author/funder, who has granted bioRxiv a license to display the preprint in perpetuity. It is made available under a CC-BY 4.0 International license.

Suppl. Fig. 8

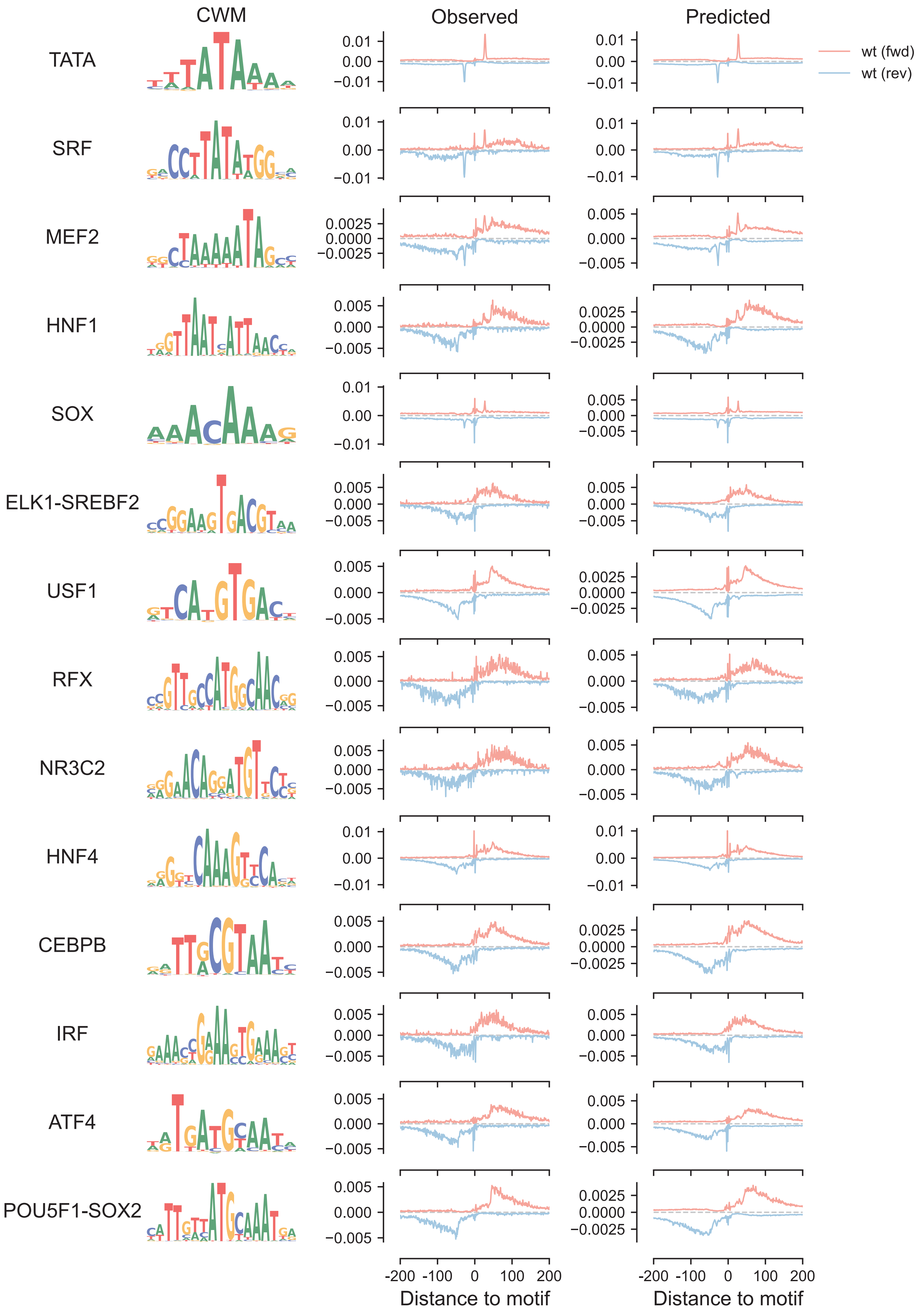

bioRxiv preprint doi: https://doi.org/10.1101/2025.09.24.676871; this version posted June 9, 2026. The copyright holder for this preprint (which was not certified by peer review) is the author/funder, who has granted bioRxiv a license to display the preprint in perpetuity. It is made available under a CC-BY 4.0 International license.

Supplementary Figure 8 | Distinct transcription initiation patterns associated with TF motifs at TREs

Representative TF motifs showing distinct effect curves on transcription profiles. *Left panel*: TF motif name and corresponding contribution weight matrix from the profile task of TF-MoDISco output. *Middle panel*: average observed PRO-cap profiles are centered at motif instances and aligned by motif orientation, with reverse-strand motifs flipped. *Right panel*: same as middle panel, but for predicted profiles. One representative ProCapNet model is shown for a given motif.

bioRxiv preprint doi: https://doi.org/10.1101/2025.09.24.676871; this version posted June 9, 2026. The copyright holder for this preprint (which was not certified by peer review) is the author/funder, who has granted bioRxiv a license to display the preprint in perpetuity. It is made available under a CC-BY 4.0 International license.

Suppl. Fig. 9

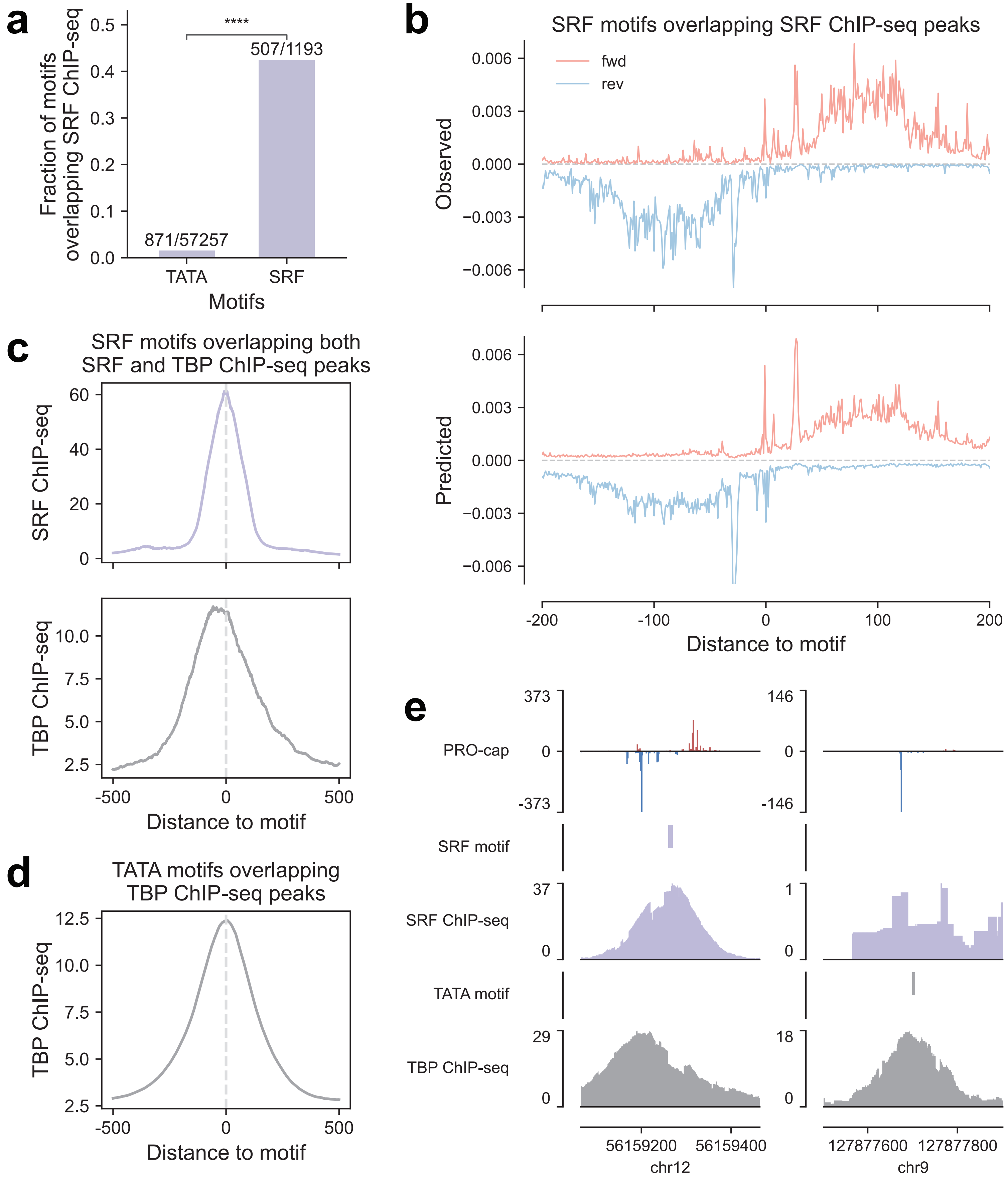

bioRxiv preprint doi: https://doi.org/10.1101/2025.09.24.676871; this version posted June 9, 2026. The copyright holder for this preprint (which was not certified by peer review) is the author/funder, who has granted bioRxiv a license to display the preprint in perpetuity. It is made available under a CC-BY 4.0 International license.

Supplementary Figure 9 | SRF motif enrichment and PRO-cap initiation profiles at ChIP-supported binding sites.

(a) Fraction of TATA and SRF motifs overlapping SRF ChIP-seq peaks (ENCFF648QJE) in ESCs.

(b) *Top*: observed PRO-cap profiles centered on SRF motif instances overlapping SRF ChIP-seq peaks, accounting for motif orientation. *Bottom*: average predicted profiles centered on the same SRF motif instances with motif orientation considered.

(c) Metaplots of SRF (ENCFF920RYL) and TBP (ENCFF206PWF) ChIP-seq signal overlapping SRF motifs. Distances are shown as ±0.5 kb from the motif center.

(d) Metaplot of TBP ChIP-seq signal overlapping TATA motifs. Distances are shown as ±0.5 kb from the motif center.

(e) PRO-cap signal and ChIP-seq tracks for SRF and TBP at a representative locus. The SRF and TATA motif instances are indicated.

Suppl. Fig. 10

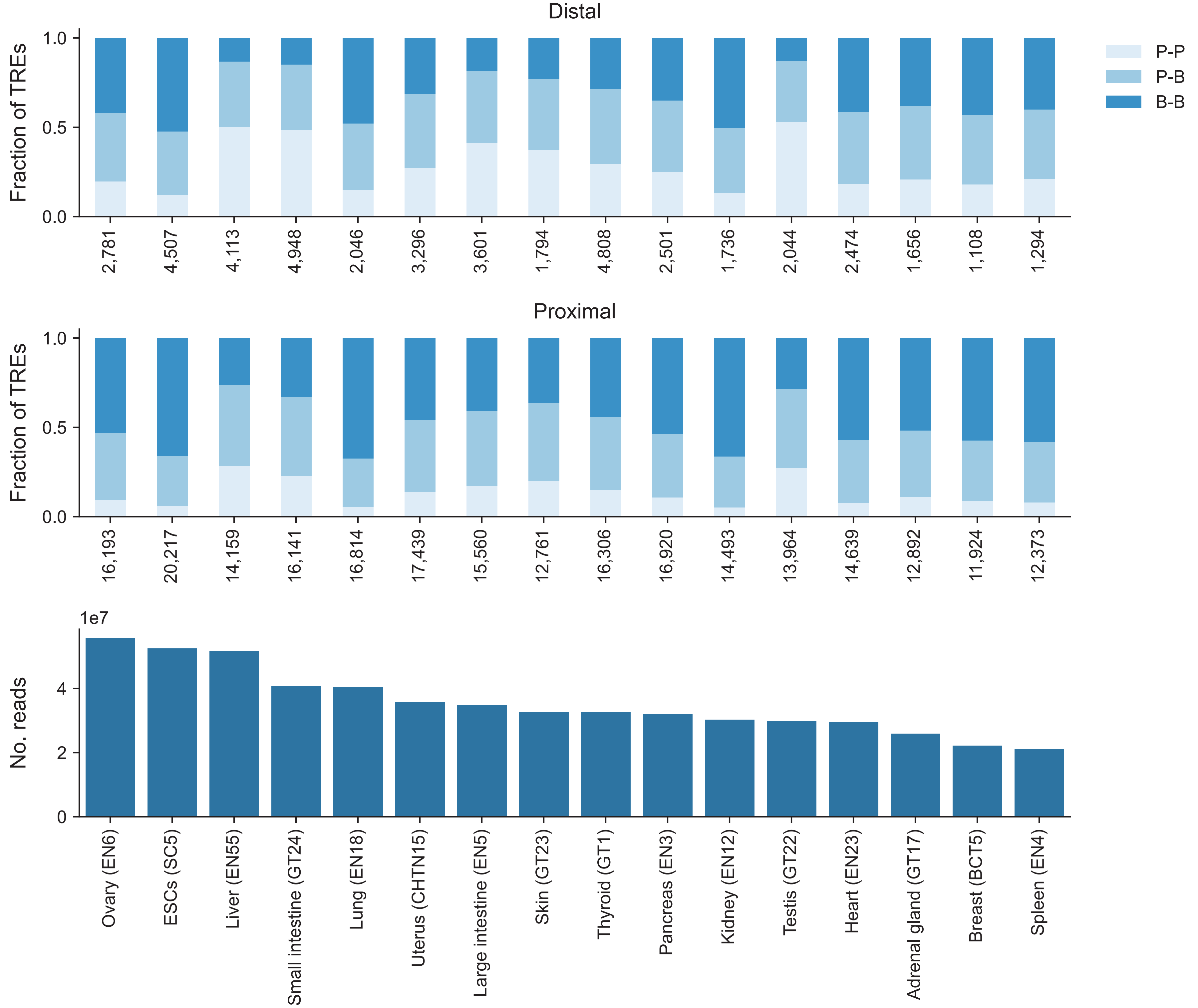
Distal
Fraction of TREs
1.0
0.5
0.0
P-P
P-B
B-B
2,781
4,507
4,113
4,948
2,046
3,296
3,601
1,794
4,808
2,501
1,736
2,044
2,474
1,656
1,108
1,294
Proximal
Fraction of TREs
1.0
0.5
0.0
16,193
20,217
14,159
16,141
16,814
17,439
15,560
12,761
16,306
16,920
14,493
13,964
14,639
12,892
11,924
12,373
1e7
No. reads
4
2
0
Ovary (EN6)
ESCs (SC5)
Liver (EN55)
Small intestine (GT24)
Lung (EN18)
Uterus (CHTN15)
Large intestine (EN5)
Skin (GT23)
Thyroid (GT1)
Pancreas (EN3)
Kidney (EN12)
Testis (GT22)
Heart (EN23)
Adrenal gland (GT17)
Breast (BCT5)
Spleen (EN4)

bioRxiv preprint doi: https://doi.org/10.1101/2025.09.24.676871; this version posted June 9, 2026. The copyright holder for this preprint (which was not certified by peer review) is the author/funder, who has granted bioRxiv a license to display the preprint in perpetuity. It is made available under a CC-BY 4.0 International license.

Supplementary Figure 10 | Distribution of TRE peak shape classes across tissue and cell types

Fractions of TREs classified as P-P, P-B, and B-B peak shapes across tissues and cell types (t*op:* distal elements; m*iddle:* proximal elements). *Bottom:* sequencing depth (number of uniquely mapped, PCR deduplicated reads) for each corresponding sample shown above.

Suppl. Fig. 11

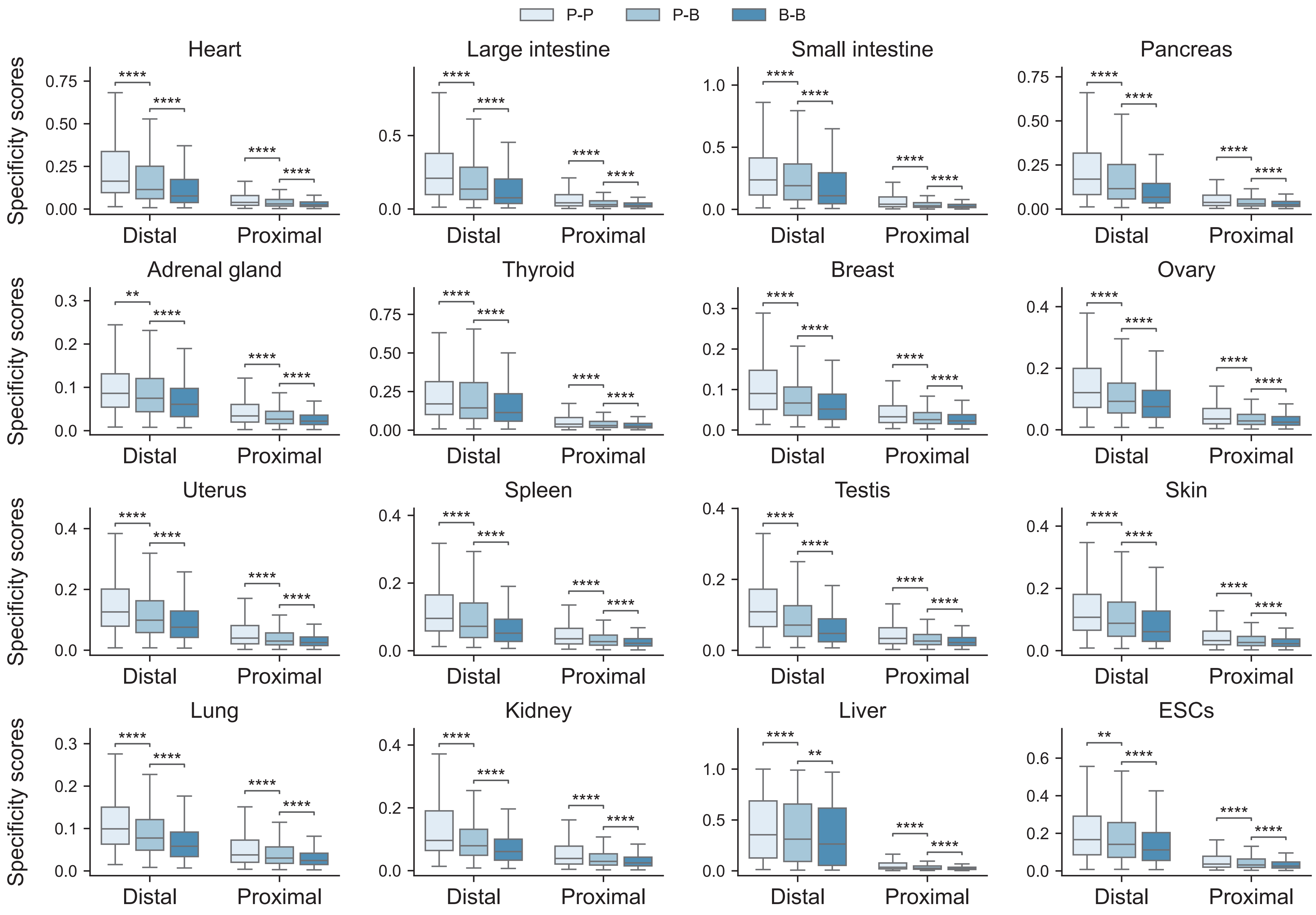
P-P
P-B
B-B
Heart
Large intestine
Small intestine
Pancreas
Adrenal gland
Thyroid
Breast
Ovary
Uterus
Spleen
Testis
Skin
Lung
Kidney
Liver
ESCs
Specificity scores
Distal
Proximal

bioRxiv preprint doi: https://doi.org/10.1101/2025.09.24.676871; this version posted June 9, 2026. The copyright holder for this preprint (which was not certified by peer review) is the author/funder, who has granted bioRxiv a license to display the preprint in perpetuity. It is made available under a CC-BY 4.0 International license.

Supplementary Figure 11 | Specificity scores of TREs with different peak shapes across tissue and cell types

Boxplots showing tissue specificity scores for TREs with P-P, P-B, and B-B peak shapes, stratified by divergent distal and proximal elements across tissues and cells. Each point represents the specificity score of an individual TRE in the indicated biosample. **, 0.001 < padj ≤ 0.01; ****, padj ≤ 0.0001.

bioRxiv preprint doi: https://doi.org/10.1101/2025.09.24.676871; this version posted June 9, 2026. The copyright holder for this preprint (which was not certified by peer review) is the author/funder, who has granted bioRxiv a license to display the preprint in perpetuity. It is made available under a CC-BY 4.0 International license.

Suppl. Fig. 12

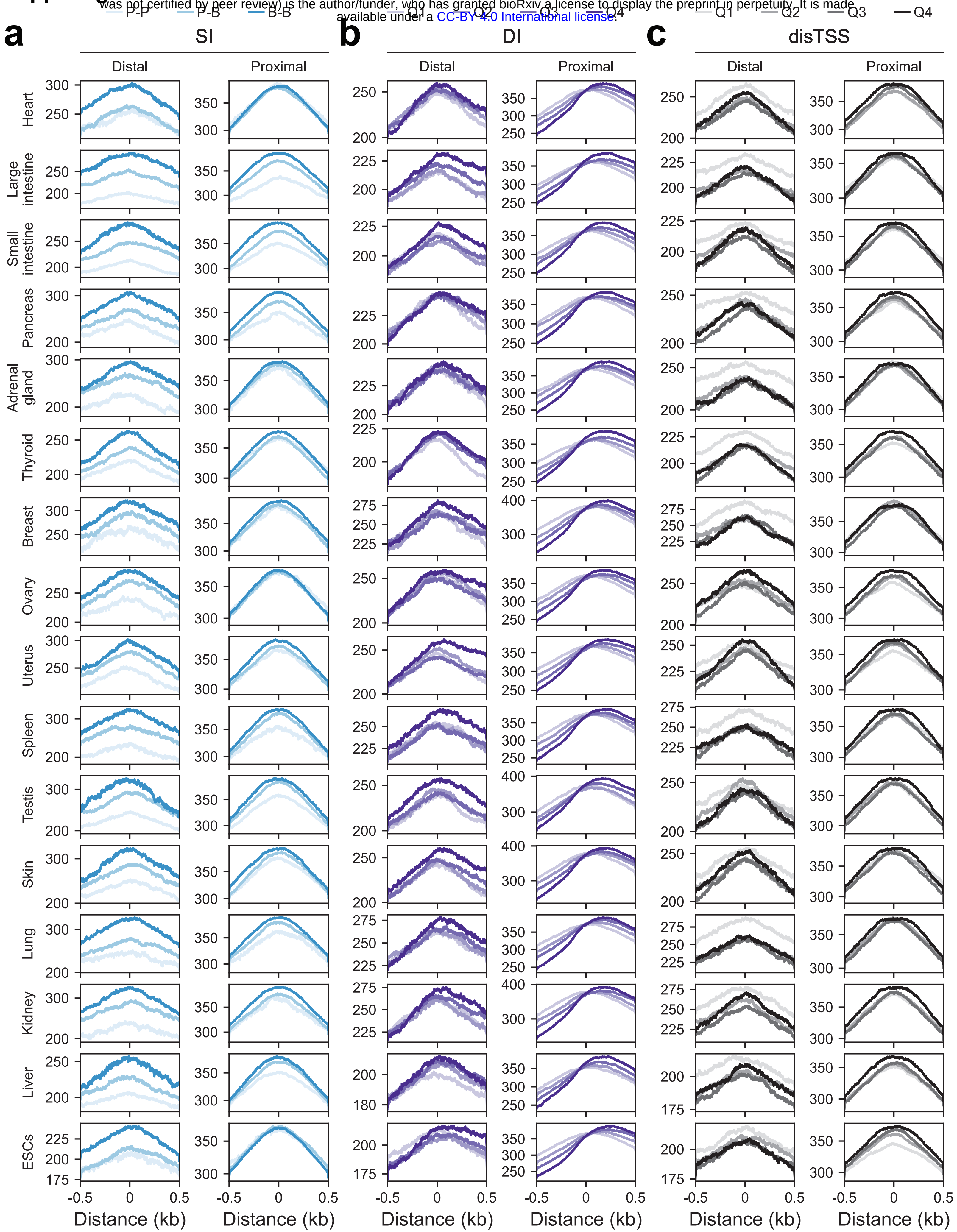

bioRxiv preprint doi: https://doi.org/10.1101/2025.09.24.676871; this version posted June 9, 2026. The copyright holder for this preprint (which was not certified by peer review) is the author/funder, who has granted bioRxiv a license to display the preprint in perpetuity. It is made available under a CC-BY 4.0 International license.

Supplementary Figure 12 | Sequence age of TREs with distinct initiation features across SI, DI, and disTSS categories

Metaplots of sequence age (million years ago) across divergent distal and proximal elements, grouped by SI (**a**), DI (**b**), and disTSS (**c**) categories. Distances are shown as ±0.5 kb from the peak center.

Suppl. Fig. 13

bioRxiv preprint doi: https://doi.org/10.1101/2025.09.24.676871; this version posted June 9, 2026. The copyright holder for this preprint (which was not certified by peer review) is the author/funder, who has granted bioRxiv a license to display the preprint in perpetuity. It is made available under a CC-BY 4.0 International license.

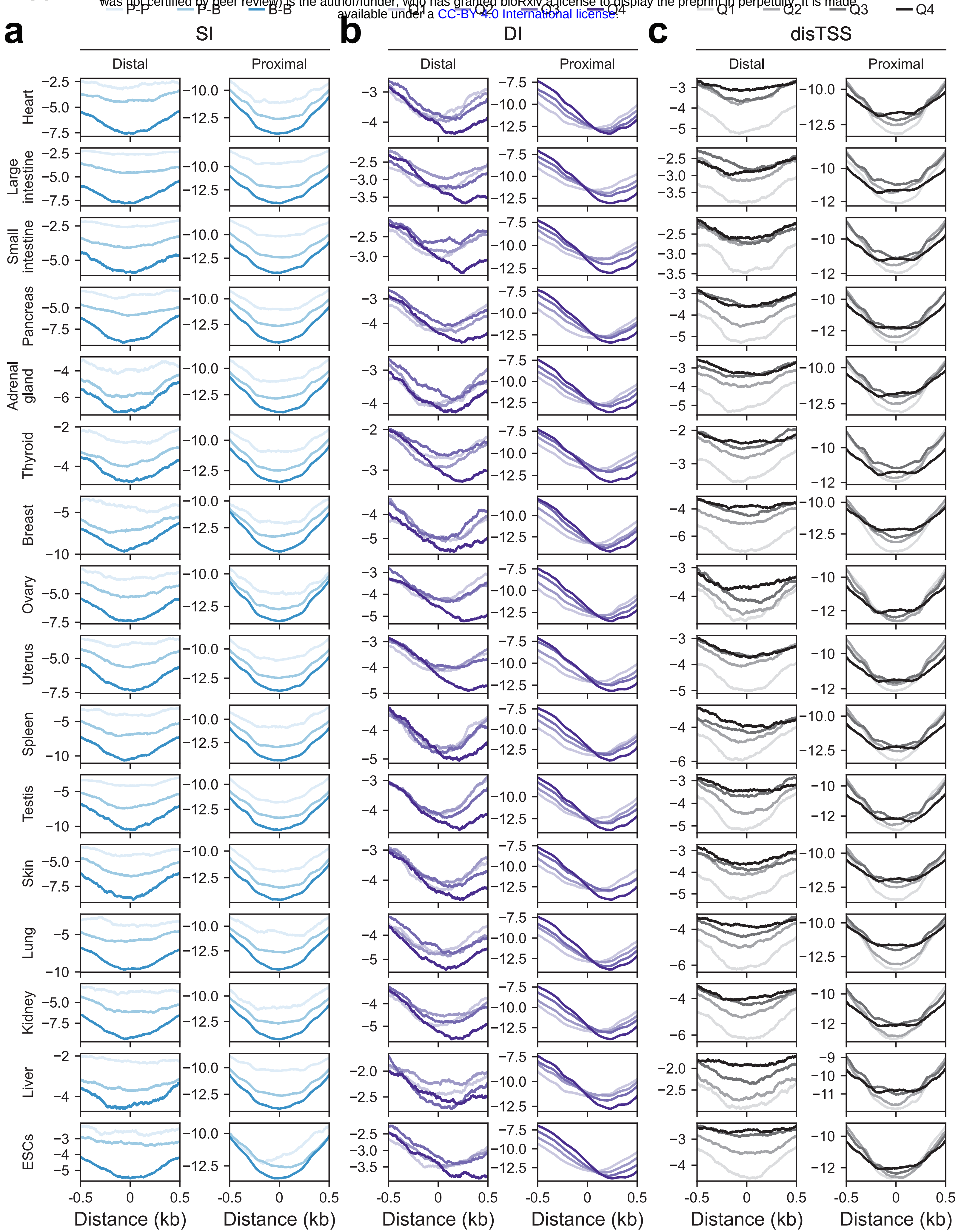

bioRxiv preprint doi: https://doi.org/10.1101/2025.09.24.676871; this version posted June 9, 2026. The copyright holder for this preprint (which was not certified by peer review) is the author/funder, who has granted bioRxiv a license to display the preprint in perpetuity. It is made available under a CC-BY 4.0 International license.

Supplementary Figure 13 | CDTS-based evolutionary constraint of TREs with distinct initiation features across tissue and cell types Metaplots of CDTS across divergent distal and proximal elements, grouped by SI (**a**), DI (**b**), and disTSS (**c**) categories. Distances are shown as ±0.5 kb from the peak center.

bioRxiv preprint doi: https://doi.org/10.1101/2025.09.24.676871; this version posted June 9, 2026. The copyright holder for this preprint (which was not certified by peer review) is the author/funder, who has granted bioRxiv a license to display the preprint in perpetuity. It is made available under a CC-BY 4.0 International license.

Suppl. Fig. 14

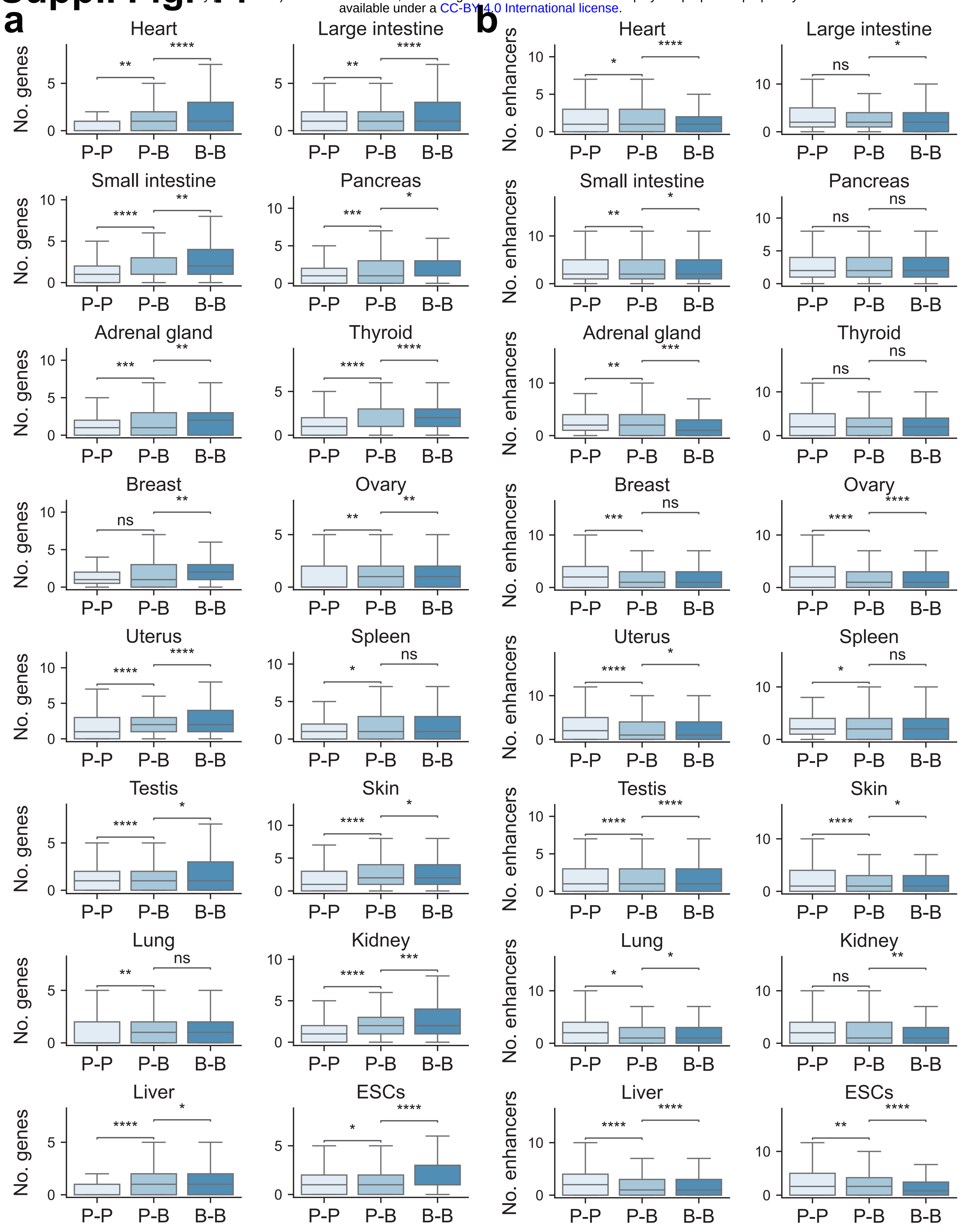

bioRxiv preprint doi: https://doi.org/10.1101/2025.09.24.676871; this version posted June 9, 2026. The copyright holder for this preprint (which was not certified by peer review) is the author/funder, who has granted bioRxiv a license to display the preprint in perpetuity. It is made available under a CC-BY 4.0 International license.

Supplementary Figure 14 | Relationship between TRE peak shape and regulatory connectivity across tissue and cell types

(a) Box plots showing the number of predicted target genes for distal TREs with different peak shapes across tissues and cells. Each point represents the number of target genes linked to a given TRE in the indicated biosample.
(b) Box plots showing the number of enhancers linked to genes with proximal TREs of different peak shapes across tissues and cells. Each point represents the number of linked enhancers for a given gene in the indicated biosample.
ns, non-significant; *, 0.01 < padj ≤ 0.05; **, 0.001 < padj ≤ 0.01; ***, 0.0001 < padj ≤ 0.001; ****, padj ≤ 0.0001.

Suppl. Fig. 15

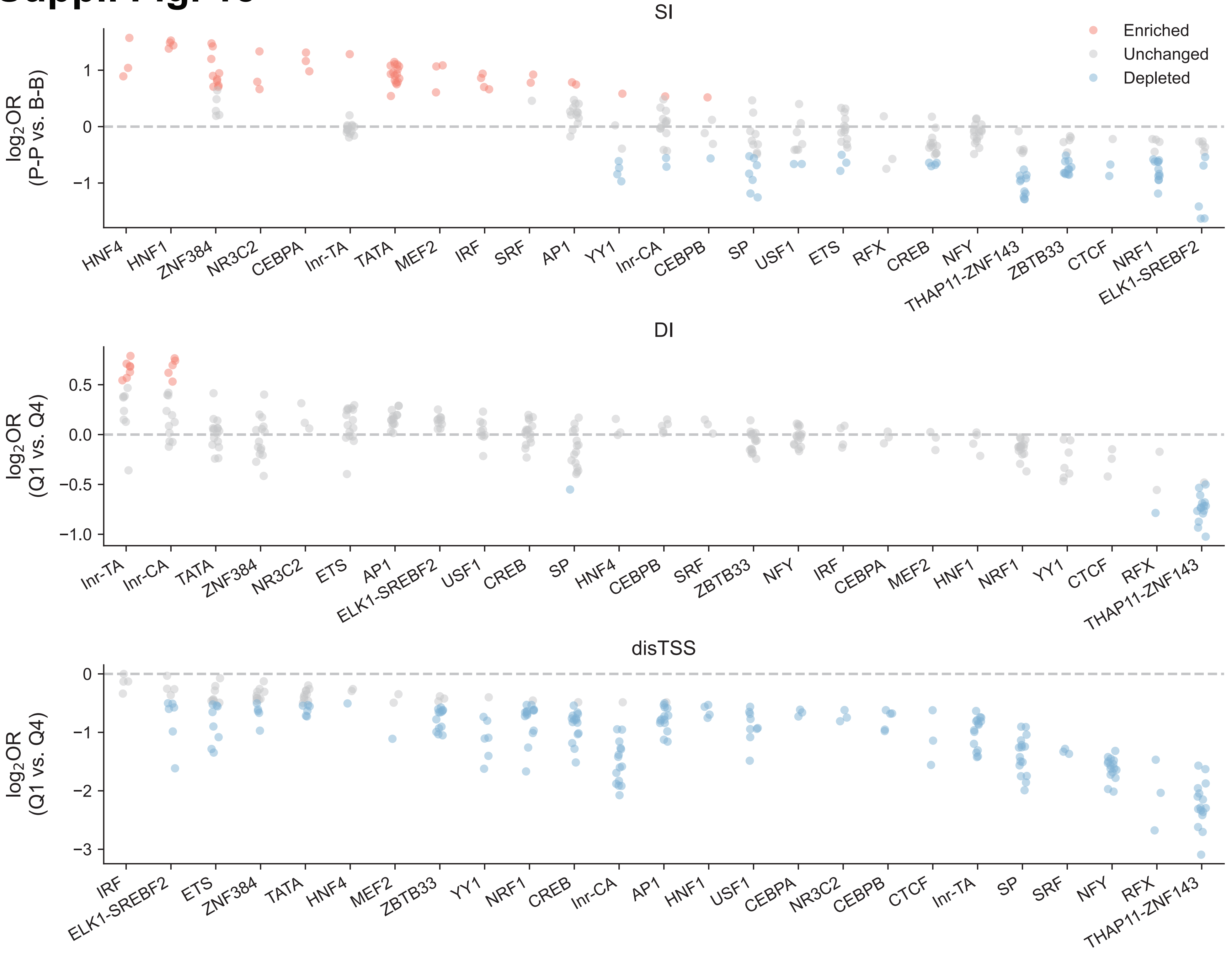

SI
Enriched
Unchanged
Depleted
log$_2$OR (P-P vs. B-B)
1
0
−1
HNF4
HNF1
ZNF384
NR3C2
CEBPA
Inr-TA
TATA
MEF2
IRF
SRF
AP1
YY1
Inr-CA
CEBPB
SP
USF1
ETS
RFX
CREB
NFY
THAP11-ZNF143
ZBTB33
CTCF
NRF1
ELK1-SREBF2
DI
log$_2$OR (Q1 vs. Q4)
0.5
0.0
−0.5
−1.0
Inr-TA
Inr-CA
TATA
ZNF384
NR3C2
ETS
AP1
ELK1-SREBF2
USF1
CREB
SP
HNF4
CEBPB
SRF
ZBTB33
NFY
IRF
CEBPA
MEF2
HNF1
NRF1
YY1
CTCF
RFX
THAP11-ZNF143
disTSS
log$_2$OR (Q1 vs. Q4)
0
−1
−2
−3
IRF
ELK1-SREBF2
ETS
ZNF384
TATA
HNF4
MEF2
ZBTB33
YY1
NRF1
CREB
Inr-CA
AP1
HNF1
USF1
CEBPA
NR3C2
CEBPB
CTCF
Inr-TA
SP
SRF
NFY
RFX
THAP11-ZNF143

bioRxiv preprint doi: https://doi.org/10.1101/2025.09.24.676871; this version posted June 9, 2026. The copyright holder for this preprint (which was not certified by peer review) is the author/funder, who has granted bioRxiv a license to display the preprint in perpetuity. It is made available under a CC-BY 4.0 International license.

Supplementary Figure 15 | Motif enrichment across TREs with distinct initiation features

Complete panel corresponding to Figure 5E. Strip plot showing $\log_2$ odds ratios of motifs across TREs stratified by transcription initiation features (P-P vs B-B for SI; Q1 vs Q4 for DI and disTSS). Each point represents one tissue type. Colors indicate enrichment (red), depletion (blue), or no change (grey). Only motifs found in at least three tissue types are shown.

Suppl. Fig. 16

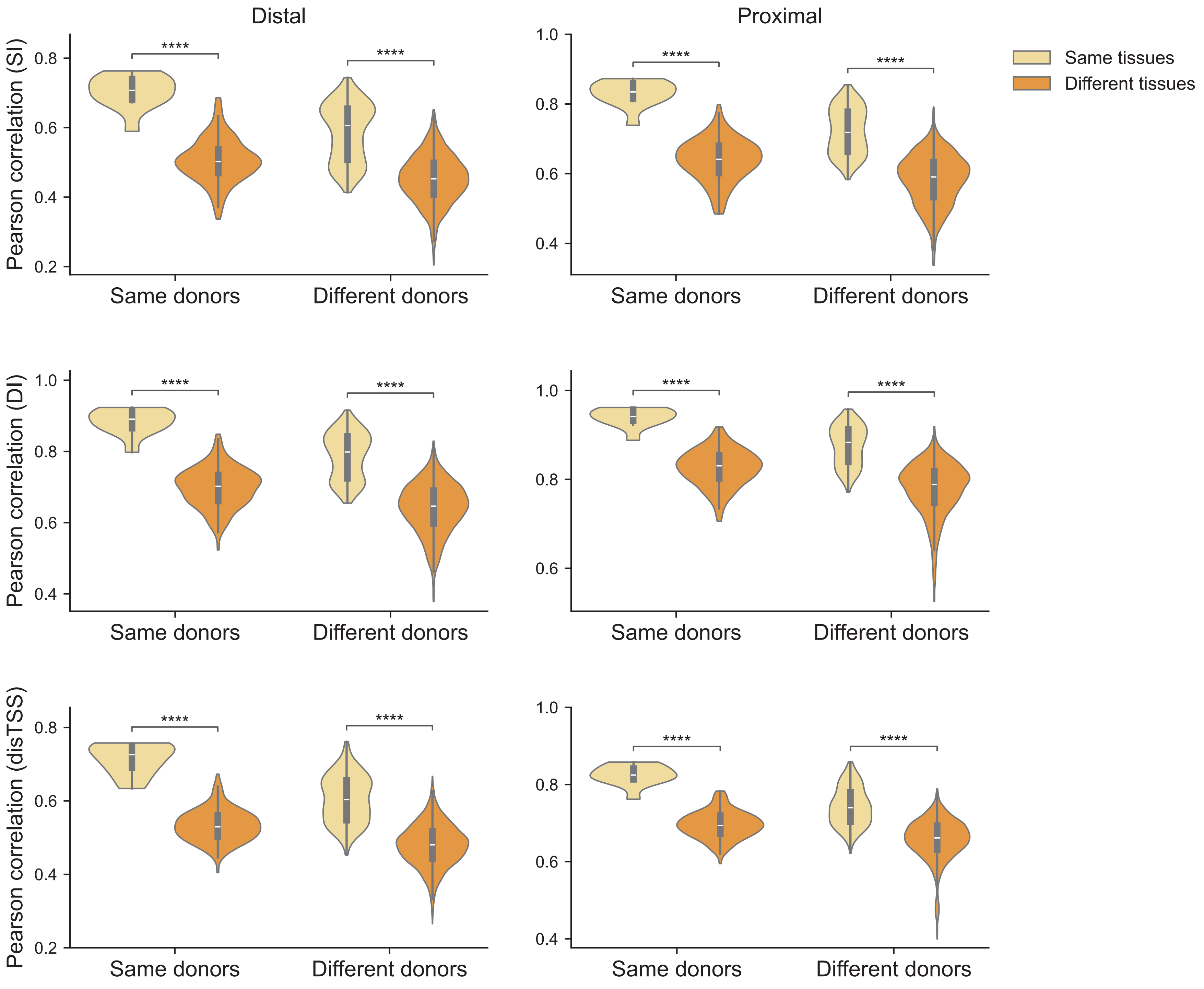
Distal
Proximal
Same tissues
Different tissues
Pearson correlation (SI)
Pearson correlation (DI)
Pearson correlation (disTSS)
Same donors
Different donors
****
0.2
0.4
0.6
0.8
1.0

bioRxiv preprint doi: https://doi.org/10.1101/2025.09.24.676871; this version posted June 9, 2026. The copyright holder for this preprint (which was not certified by peer review) is the author/funder, who has granted bioRxiv a license to display the preprint in perpetuity. It is made available under a CC-BY 4.0 International license.

Supplementary Figure 16 | Intra- and inter-tissue variation in transcription initiation profiles

Violin plots showing Pearson correlations of SI, DI, and disTSS between shared distal and proximal TREs across samples of the same or different tissue types and donors. Each point represents the correlation for a given pair of samples. ****, padj ≤ 0.0001.

bioRxiv preprint doi: https://doi.org/10.1101/2025.09.24.676871; this version posted June 9, 2026. The copyright holder for this preprint (which was not certified by peer review) is the author/funder, who has granted bioRxiv a license to display the preprint in perpetuity. It is made available under a CC-BY 4.0 International license.

Supplementary Figure 17

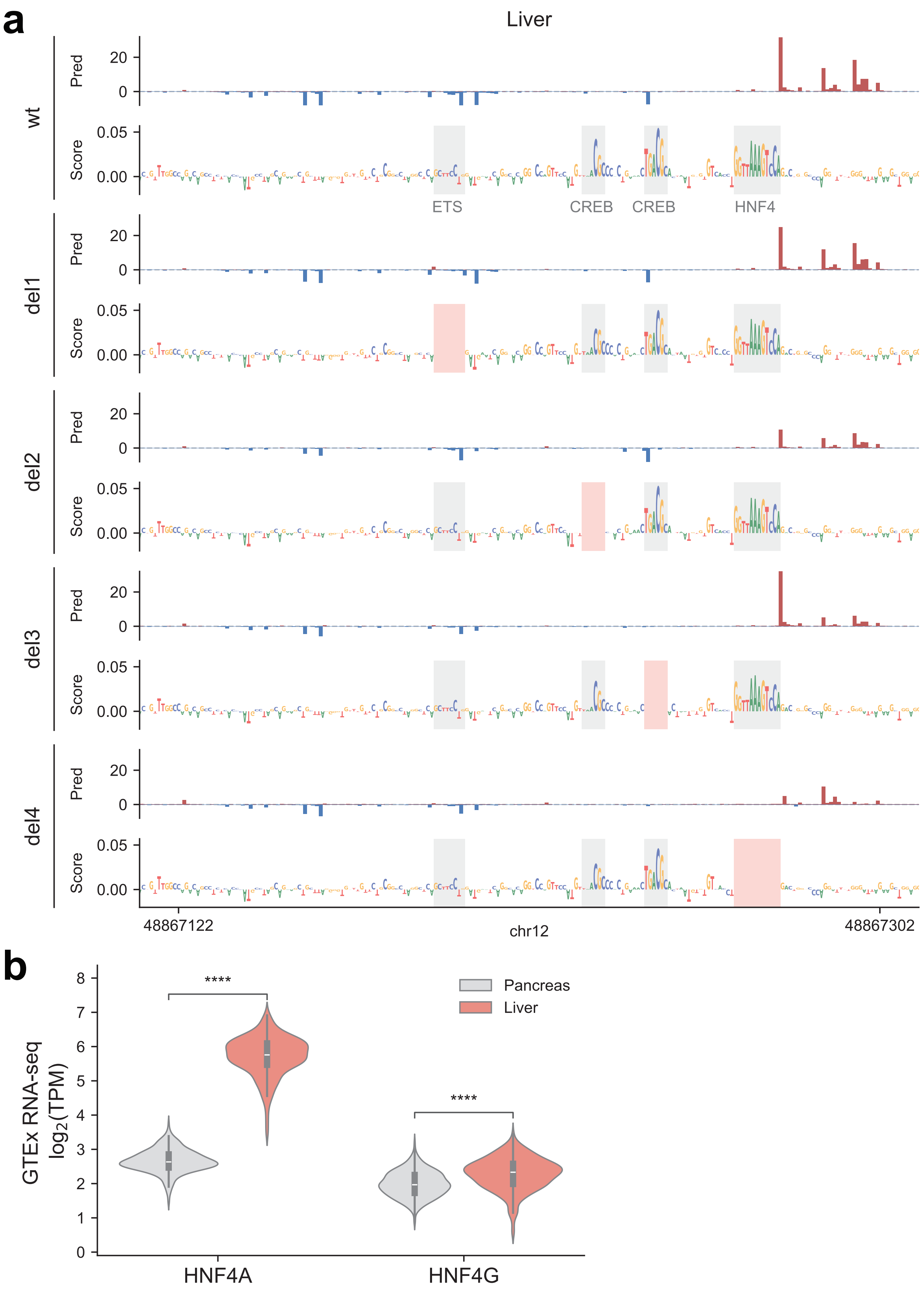

bioRxiv preprint doi: https://doi.org/10.1101/2025.09.24.676871; this version posted June 9, 2026. The copyright holder for this preprint (which was not certified by peer review) is the author/funder, who has granted bioRxiv a license to display the preprint in perpetuity. It is made available under a CC-BY 4.0 International license.

Supplementary Figure 17 | Inter-tissue effects of HNF4 motifs on transcription initiation profiles

(a) Predicted transcription profiles and contribution scores (count task) for the locus shown in Figure 5G, generated using a liver-trained ProCapNet model. The top track shows the wild type, with subsequent tracks showing the corresponding outputs following in silico deletion of the motif instance labeled in red.

(b) Expression levels ($\log_2$TPM) of HNF4A and HNF4G in pancreas and liver tissues from GTEx RNA-seq data.

Suppl. Figure 18

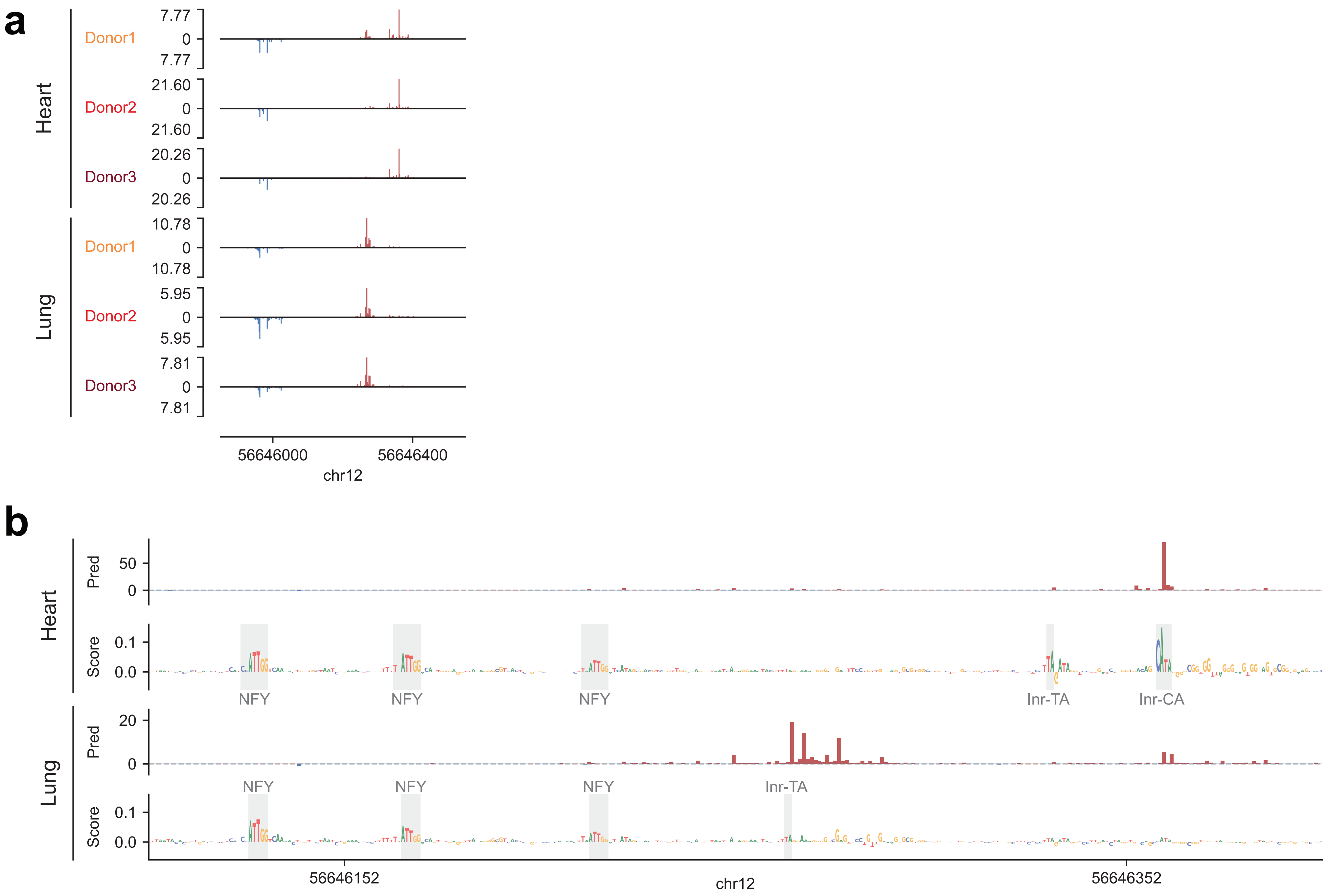

bioRxiv preprint doi: https://doi.org/10.1101/2025.09.24.676871; this version posted June 9, 2026. The copyright holder for this preprint (which was not certified by peer review) is the author/funder, who has granted bioRxiv a license to display the preprint in perpetuity. It is made available under a CC-BY 4.0 International license.

Supplementary Figure 18 | Tissue-specific preference for Initiator sequences shapes transcription initiation profiles

(a) Representative browser tracks of 5' PRO-cap signals from two tissue types (heart and lung) of three matched donors across one distal genomic locus.

(b) Predicted transcription profiles and contribution scores (profile task) for the locus shown in (a), generated using models trained on heart (top) and lung (bottom) PRO-cap data. Motifs contributing to transcription in each tissue type are labeled accordingly.

Suppl. Fig. 19

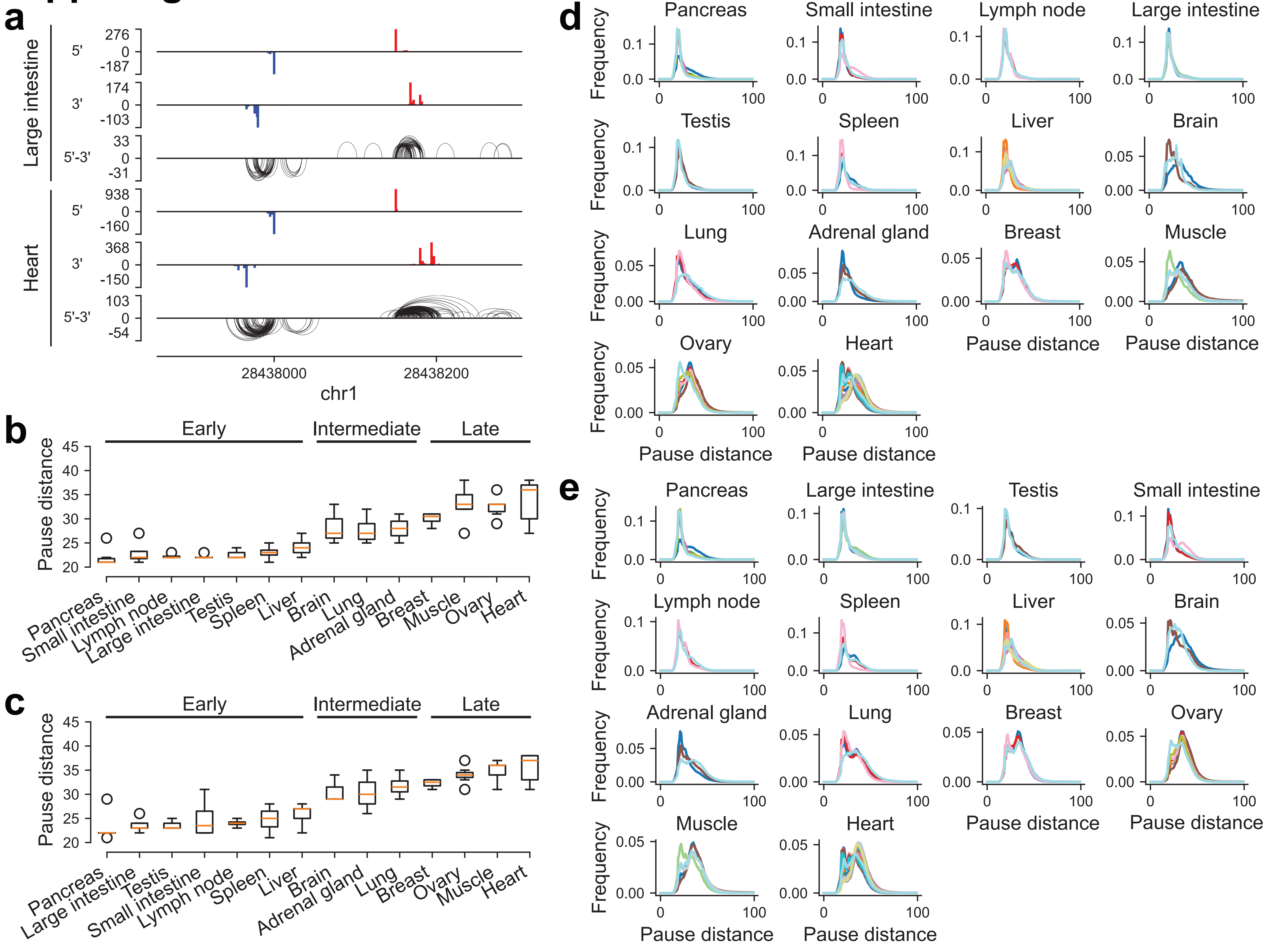
a
Large intestine
Heart
5'
3'
5'-3'
276
0
-187
174
-103
33
-31
938
-160
368
-150
103
-54
28438000
28438200
chr1
b
Early
Intermediate
Late
Pause distance
45
40
35
30
25
20
Pancreas
Small intestine
Lymph node
Large intestine
Testis
Spleen
Liver
Brain
Lung
Adrenal gland
Breast
Muscle
Ovary
Heart
c
Early
Intermediate
Late
Pause distance
Pancreas
Large intestine
Testis
Small intestine
Lymph node
Spleen
Liver
Brain
Adrenal gland
Lung
Breast
Ovary
Muscle
Heart
d
Frequency
Pancreas
Small intestine
Lymph node
Large intestine
Testis
Spleen
Liver
Brain
Lung
Adrenal gland
Breast
Muscle
Ovary
Heart
Pause distance
0.1
0.0
0.05
0.00
0
100
e
Frequency
Pancreas
Large intestine
Testis
Small intestine
Lymph node
Spleen
Liver
Brain
Adrenal gland
Lung
Breast
Ovary
Muscle
Heart
Pause distance

bioRxiv preprint doi: https://doi.org/10.1101/2025.09.24.676871; this version posted June 9, 2026. The copyright holder for this preprint (which was not certified by peer review) is the author/funder, who has granted bioRxiv a license to display the preprint in perpetuity. It is made available under a CC-BY 4.0 International license.

Supplementary Figure 19 | Variation of RNAPII pause distances of transcriptional regulatory elements across tissues

(a) Representative 5’ and 3’ PRO-cap signal tracks and pause distances for a divergent element in the large intestine (top) and heart (bottom). Loops denote 5′-3′ pause distances of individual nascent RNA molecules.

(b) Boxplot summarizing median pause distances (bp) across tissues, grouped into early, intermediate, and late pausing categories, using reads mapped to distal elements.

(c) Same as (b), but for proximal elements.

(d) Distribution of pause distances (bp) across tissues, with each line representing one sample of a given tissue type, using reads mapped to distal elements.

(e) Same as (d), but for proximal elements.

bioRxiv preprint doi: https://doi.org/10.1101/2025.09.24.676871; this version posted June 9, 2026. The copyright holder for this preprint (which was not certified by peer review) is the author/funder, who has granted bioRxiv a license to display the preprint in perpetuity. It is made available under a CC-BY 4.0 International license.

Suppl. Fig. 20

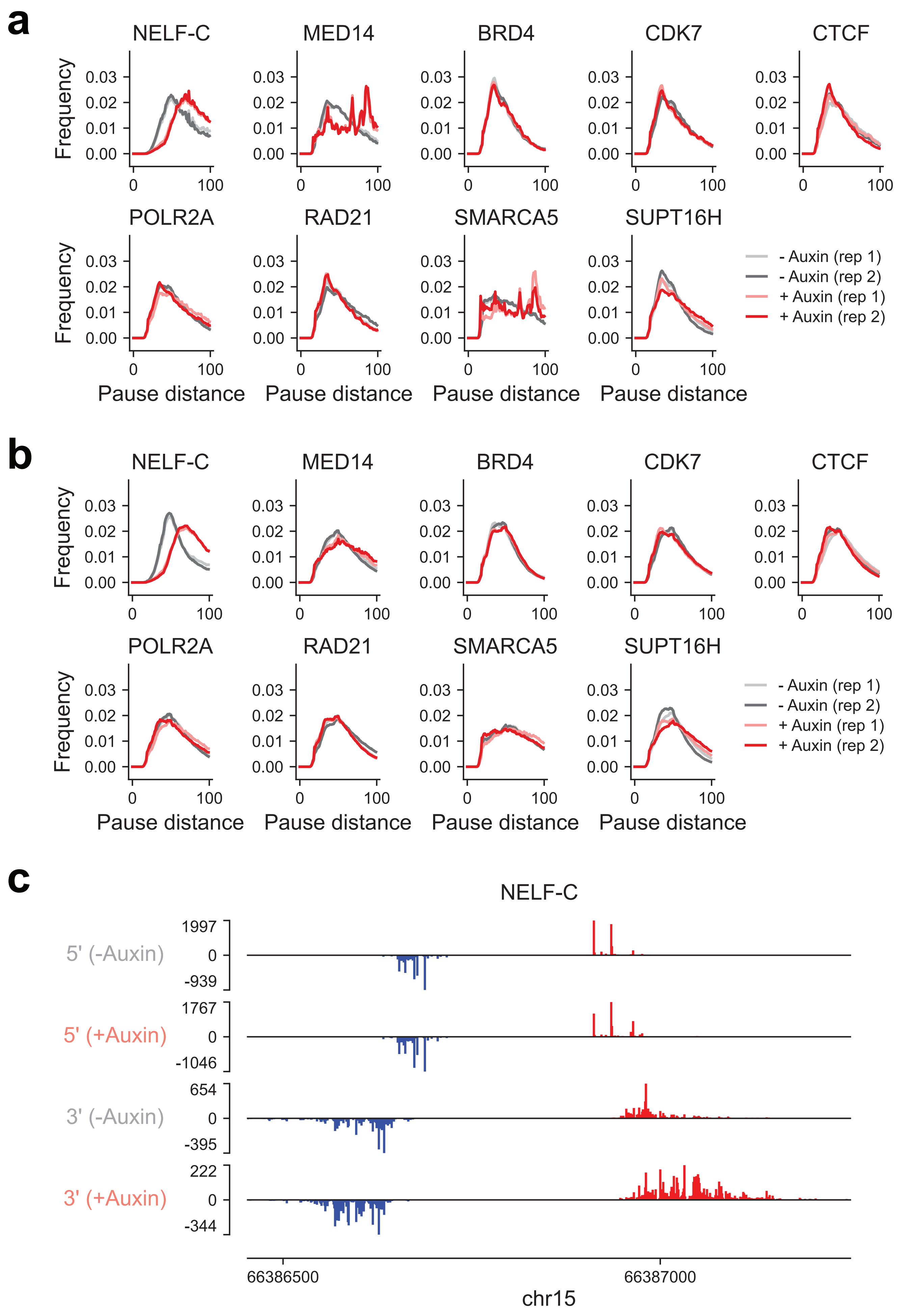

bioRxiv preprint doi: https://doi.org/10.1101/2025.09.24.676871; this version posted June 9, 2026. The copyright holder for this preprint (which was not certified by peer review) is the author/funder, who has granted bioRxiv a license to display the preprint in perpetuity. It is made available under a CC-BY 4.0 International license.

Supplementary Figure 20 | Genome-wide distribution of pause distances in transcriptional regulatory elements following depletion of different factors

(a) Genome-wide distribution of pause distances for distal elements before and after acute depletion of the indicated factors using auxin-inducible degron systems. All degron-tagged experiments were performed in HCT116 cells, except for NELF-C, which was generated in DLD-1 cells. The NELF-C degron-tagged dataset was obtained from a previously published study (GSE144786) and reprocessed using our pipeline. Two biological replicates were included per condition (−auxin and +auxin).

(b) Same as (a), but for proximal elements.

(c) Representative 5’ and 3’ PRO-cap signal tracks (merged across replicates) of an element before and after NELF-C degradation in DLD-1 cells.

bioRxiv preprint doi: https://doi.org/10.1101/2025.09.24.676871; this version posted June 9, 2026. The copyright holder for this preprint (which was not certified by peer review) is the author/funder, who has granted bioRxiv a license to display the preprint in perpetuity. It is made available under a CC-BY 4.0 International license.

Suppl. Fig. 21

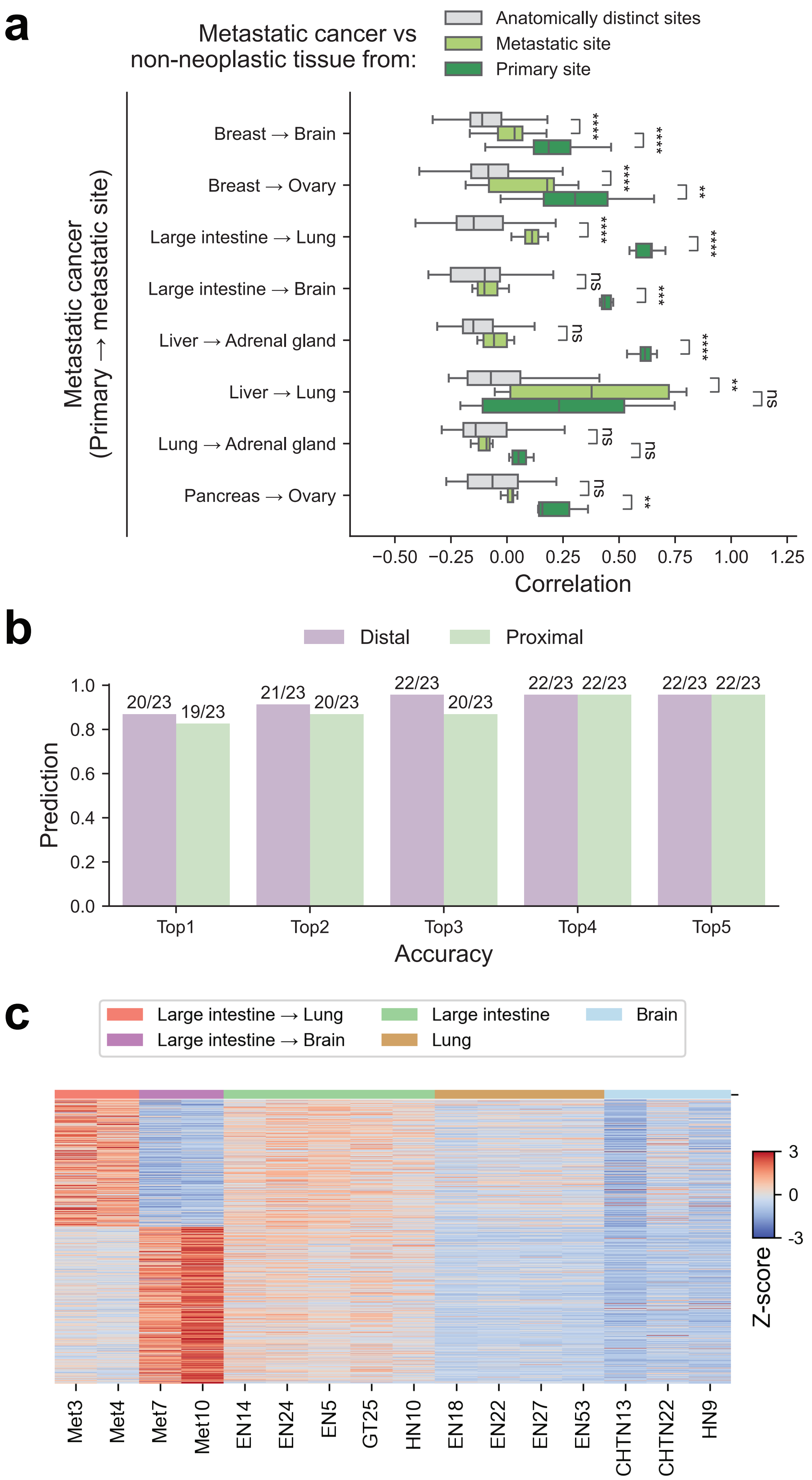

bioRxiv preprint doi: https://doi.org/10.1101/2025.09.24.676871; this version posted June 9, 2026. The copyright holder for this preprint (which was not certified by peer review) is the author/funder, who has granted bioRxiv a license to display the preprint in perpetuity. It is made available under a CC-BY 4.0 International license.

Supplementary Figure 21 | Transcriptional regulatory element signatures predict the tissue of origin in patient-derived tumors

(a) Pairwise comparisons of divergent proximal TRE expression profiles between metastatic tumors (n=23) and non-neoplastic tissues from the primary (dark green), metastatic (light green), and unrelated sites (gray). ns, non-significant; **, $0.001 < padj \leq 0.01$; ***, $0.0001 < padj \leq 0.001$; ****, $padj \leq 0.0001$.

(b) Accuracy of tissue-of-origin prediction for metastatic tumors using models trained on divergent distal and proximal PRO-cap profiles. Results are shown for top-1 through top-5 ranked predictions.

(c) Heatmap of differentially expressed divergent distal TREs (n=1,128) between lung and brain metastases. Z-scores of PRO-cap signals were calculated across metastatic samples and related non-neoplastic tissues.

bioRxiv preprint doi: https://doi.org/10.1101/2025.09.24.676871; this version posted June 9, 2026. The copyright holder for this preprint (which was not certified by peer review) is the author/funder, who has granted bioRxiv a license to display the preprint in perpetuity. It is made available under a CC-BY 4.0 International license.

Supplementary Table legends

Supplementary Table 1 | Metadata of PRO-cap libraries
Summary of biosample, experimental, and sequencing details for all libraries generated using PRO-cap. PBMC: peripheral blood mononuclear cell; T1D: type 1 diabetes; iPSC: induced pluripotent stem cell; ESC: embryonic stem cell; CML: chronic myelogenous leukemia; B-ALL: B-cell acute lymphocytic leukemia; GCB-DLBCL: germinal center B-cell diffuse large B-cell lymphoma; MCL: mantle cell lymphoma.

Supplementary Table 2 | Motif enrichment across different TRE sets
Summary of motif enrichment and associated statistics for three TRE set comparisons: (1) TRE modules 1-10 from a leukemia and lymphoma cell line panel (LL-M1 to LL-M10); (2) T cells from T1D patients versus healthy donors (T1D-Up and T1D-Down); and (3) CRC metastases to lung versus brain (CRC Met-Brain and CRC Met-Lung). De novo motif enrichment is reported for the leukemia and lymphoma panel, while known motif enrichment is reported for the T1D and CRC metastasis comparisons.

Supplementary Table 3 | Datasets and analyses across different annotations
Details of datasets and analyses across annotations. The table is organized into four sheets: (1) data sources for tissue-matched comparisons across assays in Fig. 2a; (2) the total number of peaks for each annotation, along with the number and fraction of overlapping peaks with PRO-cap elements, reported for both PRO-cap–anchored and other annotation-anchored comparisons (irrespective of genomic location), for each matched tissue type, based on the datasets described in Sheet 1; (3) ProCapNet model training datasets; and (4) E2G prediction of promoter–enhancer (P-E) link datasets.

Supplementary Table 4 | Primer sequences
Details of cloning primers and TRE sequences used in the section “Tissue-specific effects of disease- and trait-associated variants.”
