# Supplementary material for: The regulatory landscape of nascent transcription in human health and disease
Source: bioRxiv. 2025 Sep 26:2025.09.24.676871. Preprint. [Version 1] doi: 10.1101/2025.09.24.676871 (PMC12485982; doi:10.1101/2025.09.24.676871)
Supplement: Supplementary file 1 [file media-1.pdf]

Supplementary Information

This file contains Supplementary Figures 1-10, Supplementary Tables 1-4, and their associated legends.

Supplementary Figure 1

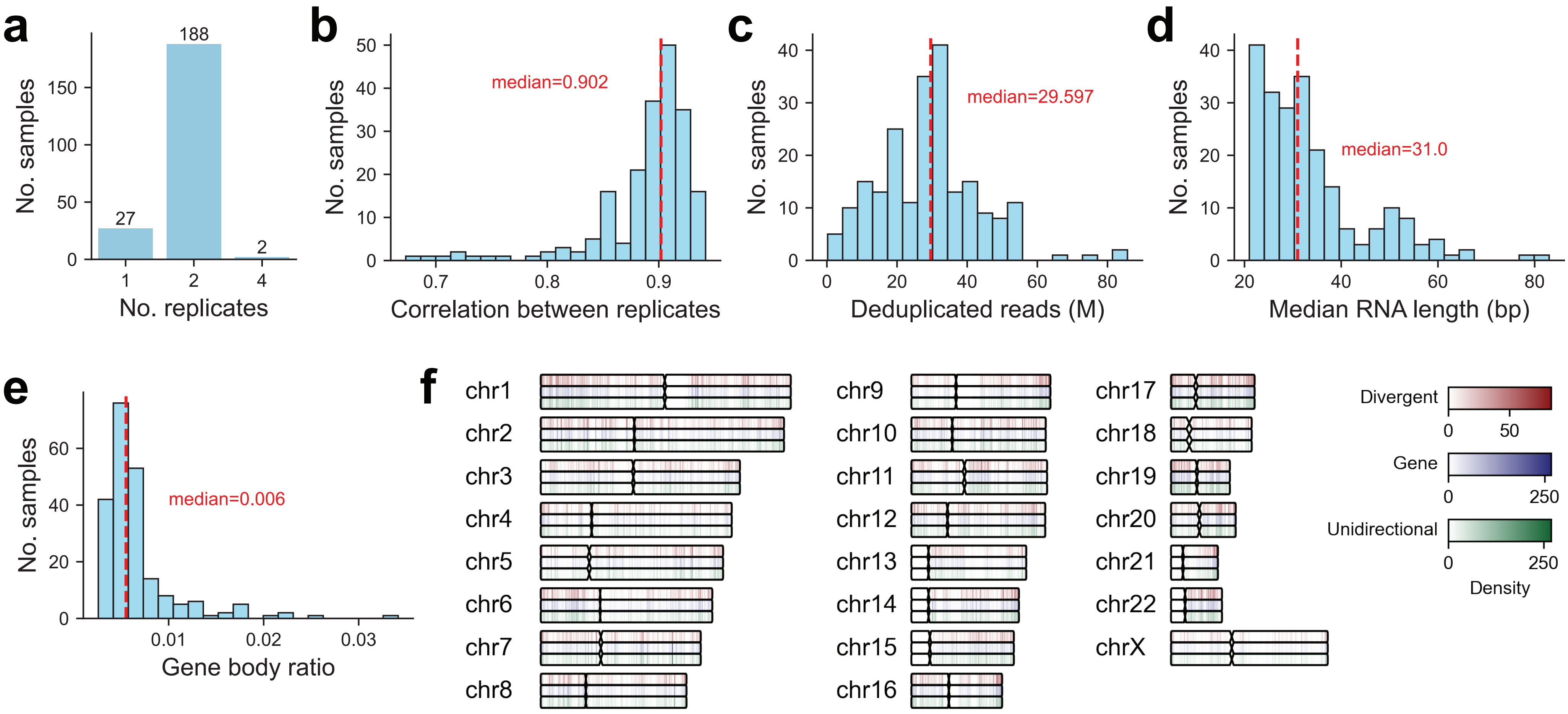

Supplementary Fig. 1 | Overview of PRO-cap data quality and genomic distribution

(a) Barplot showing the number of replicates per sample.

(b) Histogram of Pearson correlations between replicates for samples with at least two replicates.

(c) Histogram of gene body ratios across samples.

(d) Histogram of deduplicated uniquely mapped reads (in millions) across samples.

(e) Histogram of median RNA length (bp) across samples.

(f) Genomic distribution of divergent (red) and unidirectional (blue) TREs across the genome, with genes shown in green. Densities represent $\log_{10}$-transformed counts in 100 Mb bins.

Supplementary Figure 2

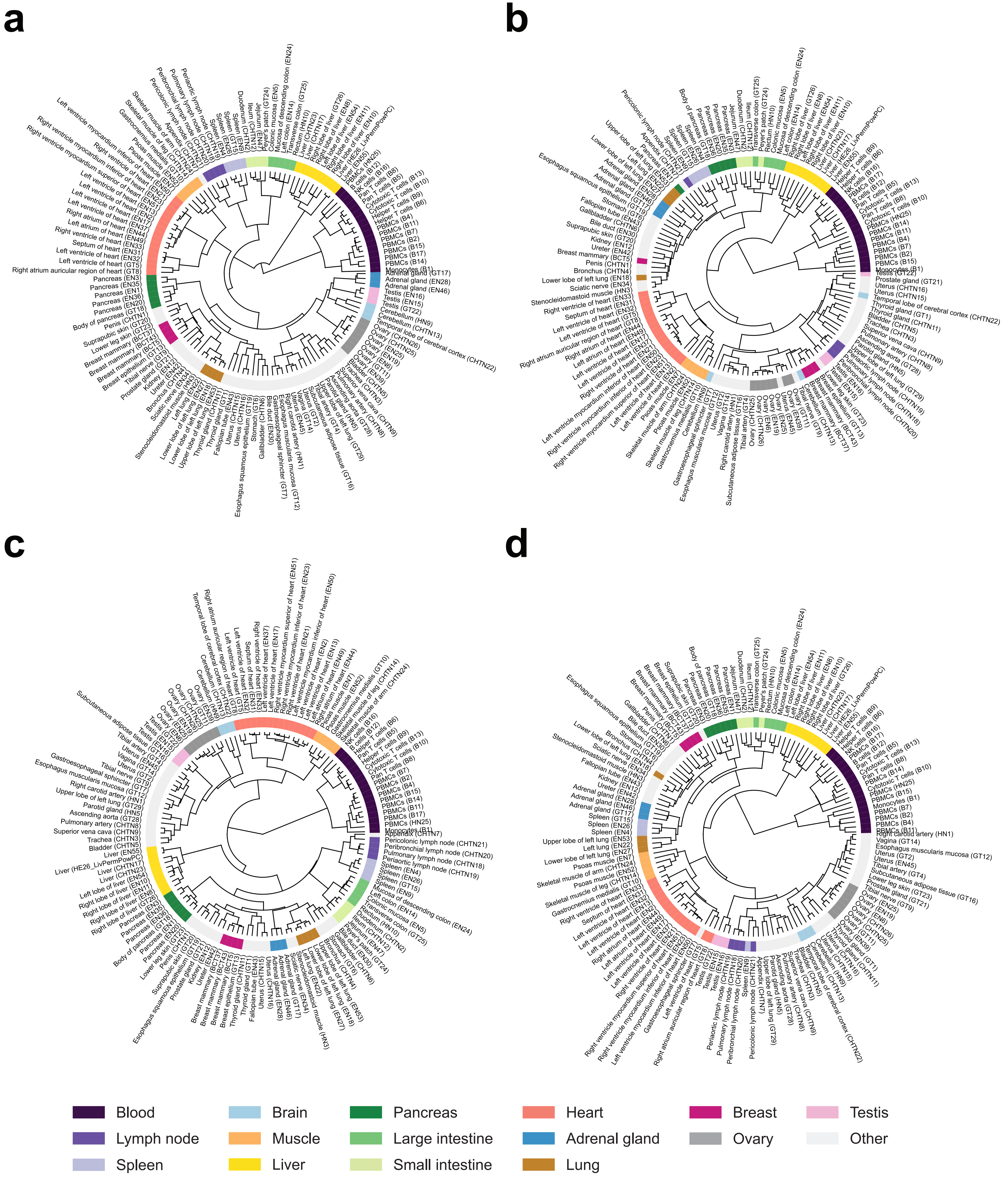

Supplementary Fig. 2 | Hierarchical clustering of samples by different TRE categories

(a) Labeled dendrogram corresponding to Fig. 2a, showing hierarchical clustering of tissue samples based on normalized PRO-cap expression at divergent distal elements. Sample names are labeled, and subtrees representing a single tissue type (≥3 samples) are highlighted.

(b-d) Same as (a), but for unidirectional distal (b), divergent proximal (c), and unidirectional proximal (d) elements.

Supplementary Figure 3

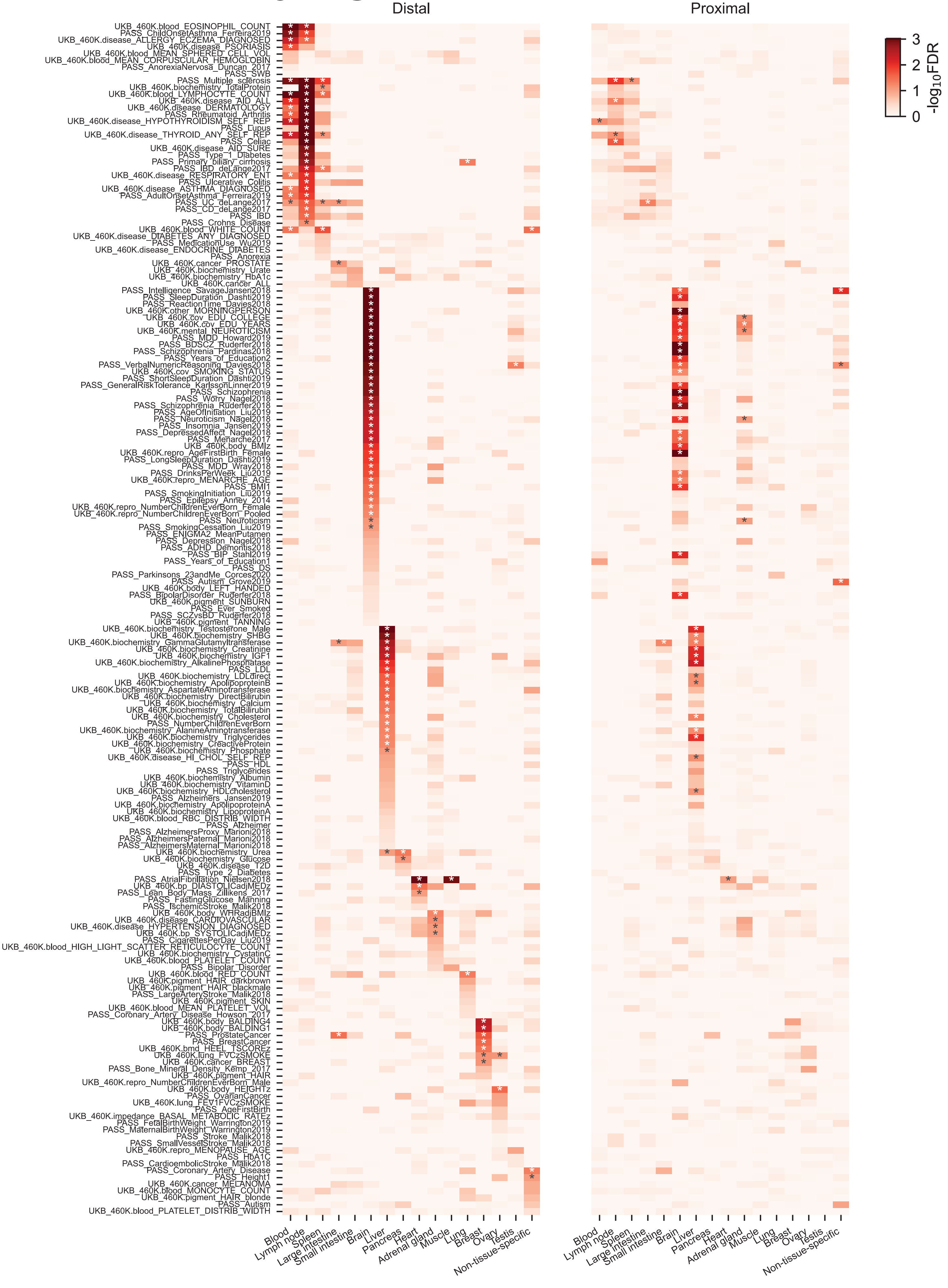

Supplementary Fig. 3 | Tissue-specific effects of disease- and trait-associated variants

Full panel of the significance for heritability enrichment in human diseases and complex traits from 176 studies across divergent distal and proximal TRE annotations. Asterisks (*) denote FDR < 0.1 after Benjamini-Hochberg correction.

Supplementary Figure 4

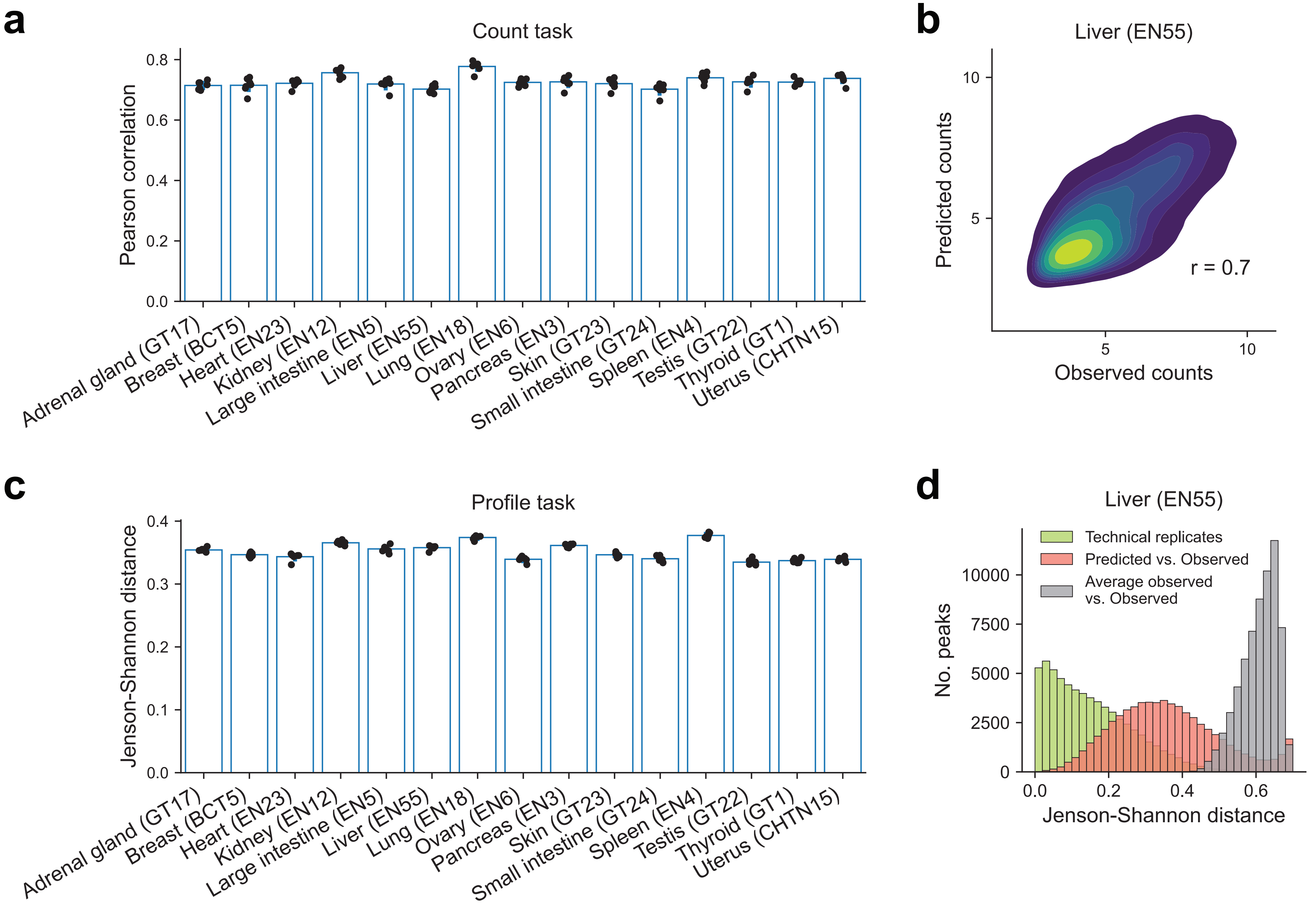

Supplementary Fig. 4 | ProCapNet model performance across tissues

(a) Barplots showing the Pearson correlations between log-transformed observed and predicted total counts on held-out test chromosomes across 7-fold cross-validation for each model. Each datapoint represents one-fold.

(b) Density plot of log-transformed observed and predicted counts at PRO-cap peaks from held-out test chromosomes across 7-fold cross-validation, based on the model trained on the liver sample EN55.

(c) Same as (a), but showing the mean Jenson-Shannon distance between observed and predicted profiles for each model.

(d) Distribution of Jensen-Shannon distances between observed and predicted base-resolution profiles at PRO-cap peaks from held-out test chromosomes across 7-fold cross-validation (red), based on the model trained on the liver sample EN55. For comparison, distances between observed profiles from two technical replicates of EN55 are shown in green (upper bound), and distances between observed profiles and profiles averaged over all peaks are shown in grey (baseline).

Supplementary Figure 5

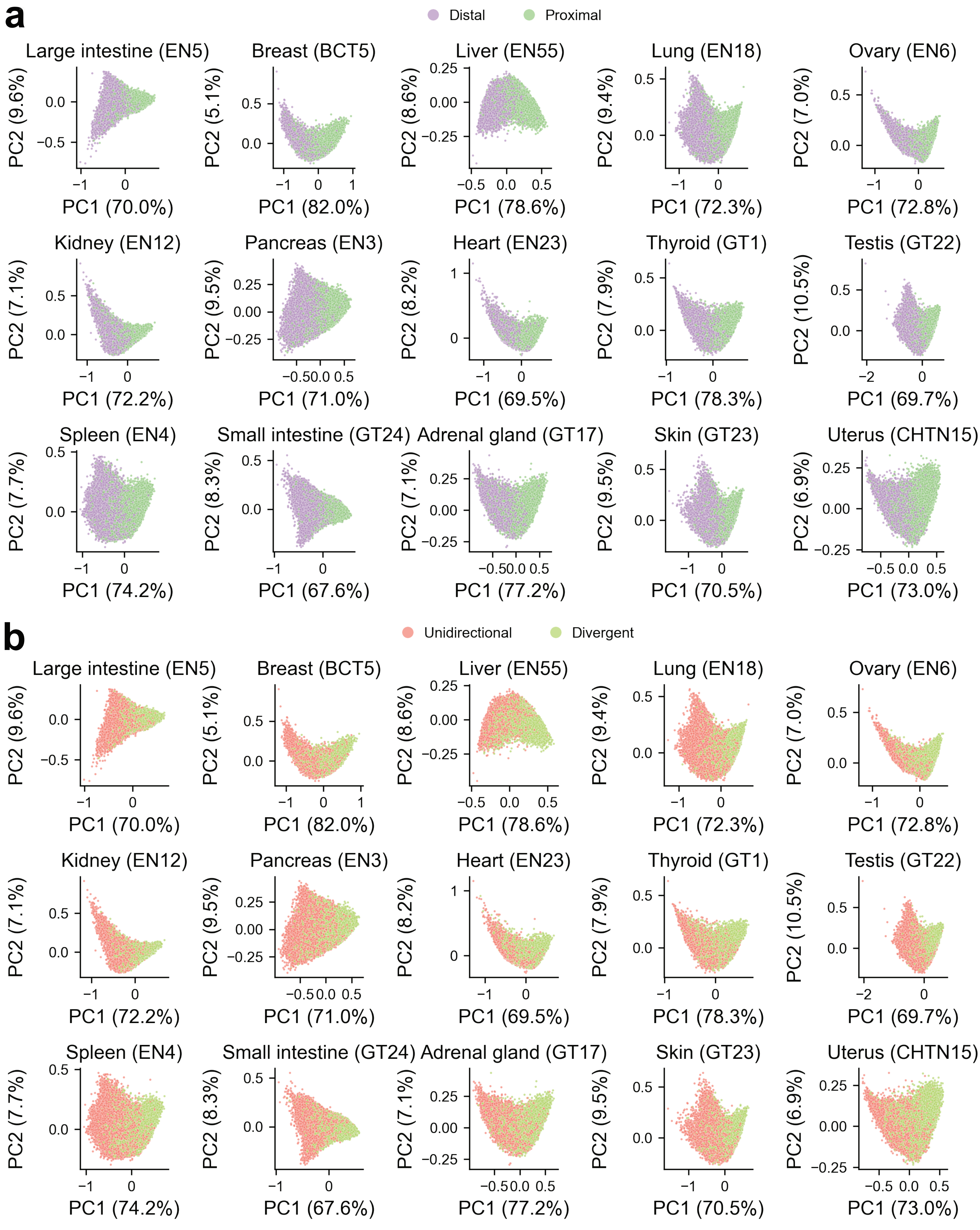

Supplementary Fig. 5 | Principal component analysis of ProCapNet embeddings

(a) PCA of ProCapNet embeddings for all PRO-cap peaks in each model, colored by distal versus proximal categories.

(b) Same as (a), but colored by unidirectional versus divergent elements.

Supplementary Figure 6

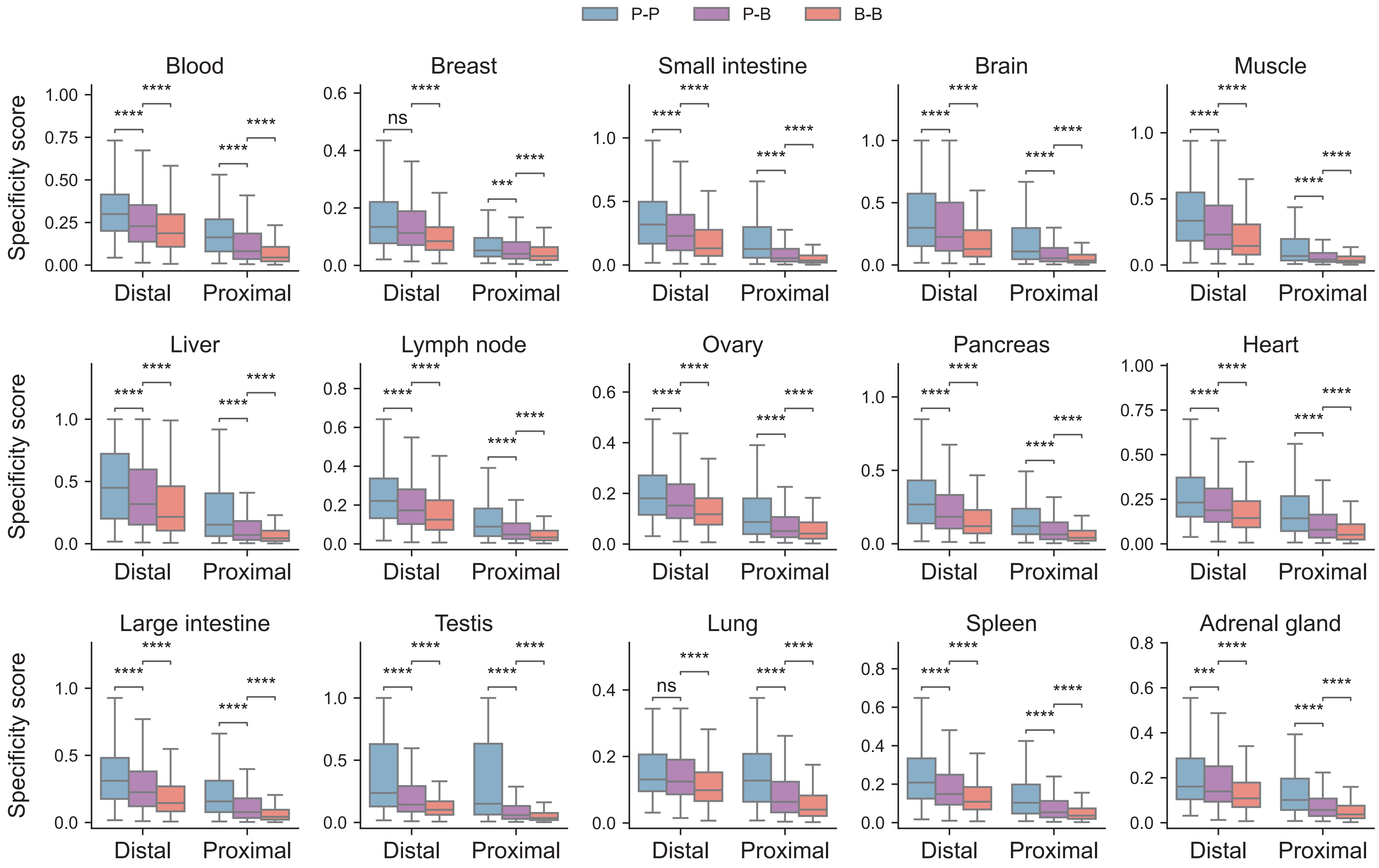

Supplementary Fig. 6 | Tissue specificity scores of TREs with different peak shapes across tissues

Boxplots showing tissue specificity scores for TREs with P-P, P-B, and B-B peak shapes, stratified by divergent distal and proximal elements across tissues. Each point represents the specificity score of an individual TRE in the indicated tissue.

Supplementary Figure 7

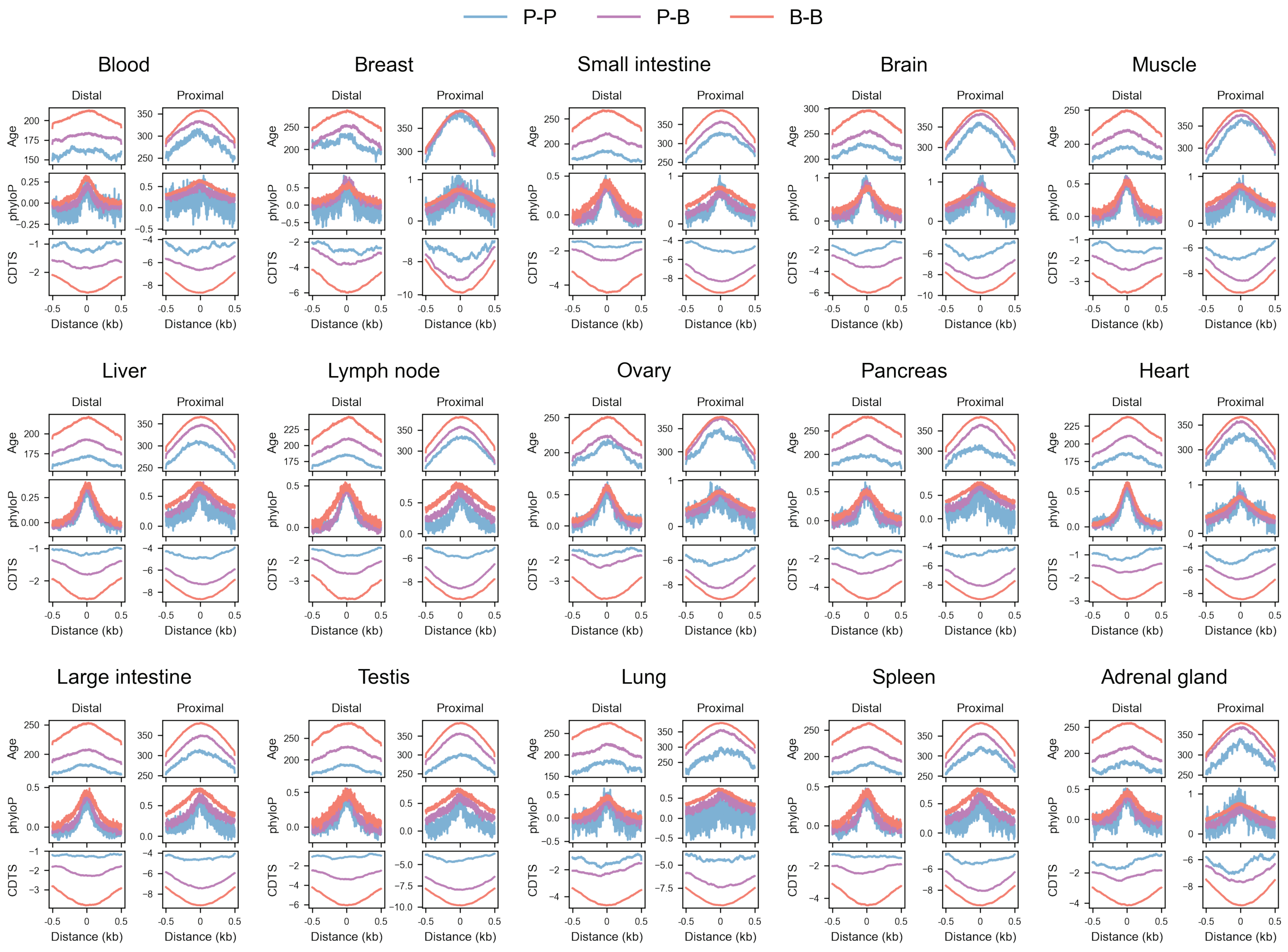

Supplementary Fig. 7 | Evolutionary conservation and constraint patterns of TREs with distinct peak shapes across tissues Metaplots of sequence age (million years ago), phyloP, and CDTS across divergent distal and proximal elements, grouped by peak shape, in different tissues. Distances are shown as ±0.5 kb from the peak center.

Supplementary Figure 8

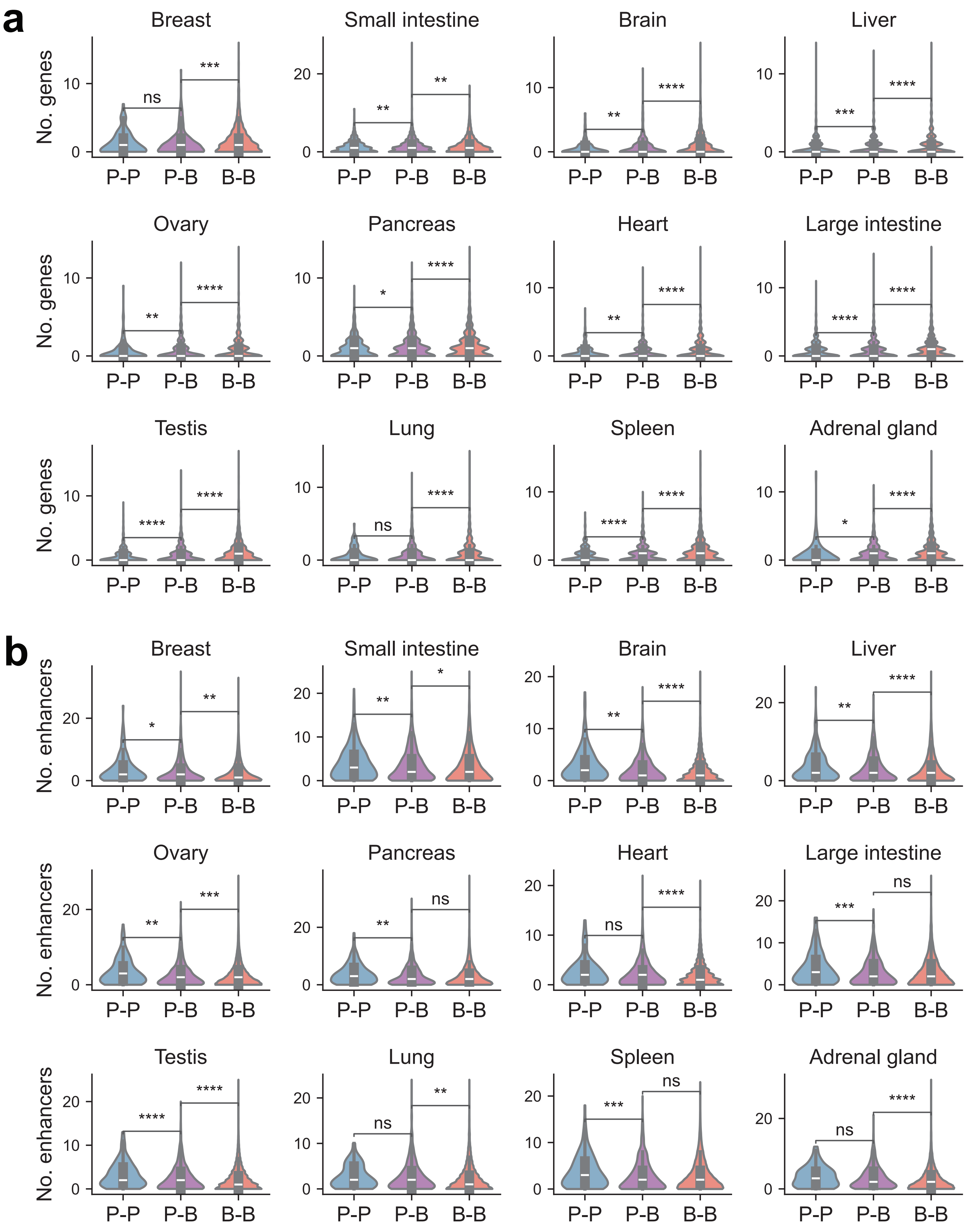

Supplementary Fig. 8 | Relationship between TRE peak shape and regulatory connectivity across tissues

(a) Violin plots showing the number of predicted target genes for distal TREs with different peak shapes across tissues. Each point represents the number of target genes linked to a given TRE in the indicated tissue.

(b) Violin plots showing the number of enhancers linked to genes with proximal TREs of different peak shapes across tissues. Each point represents the number of linked enhancers for a given gene in the indicated tissue.

Supplementary Figure 9

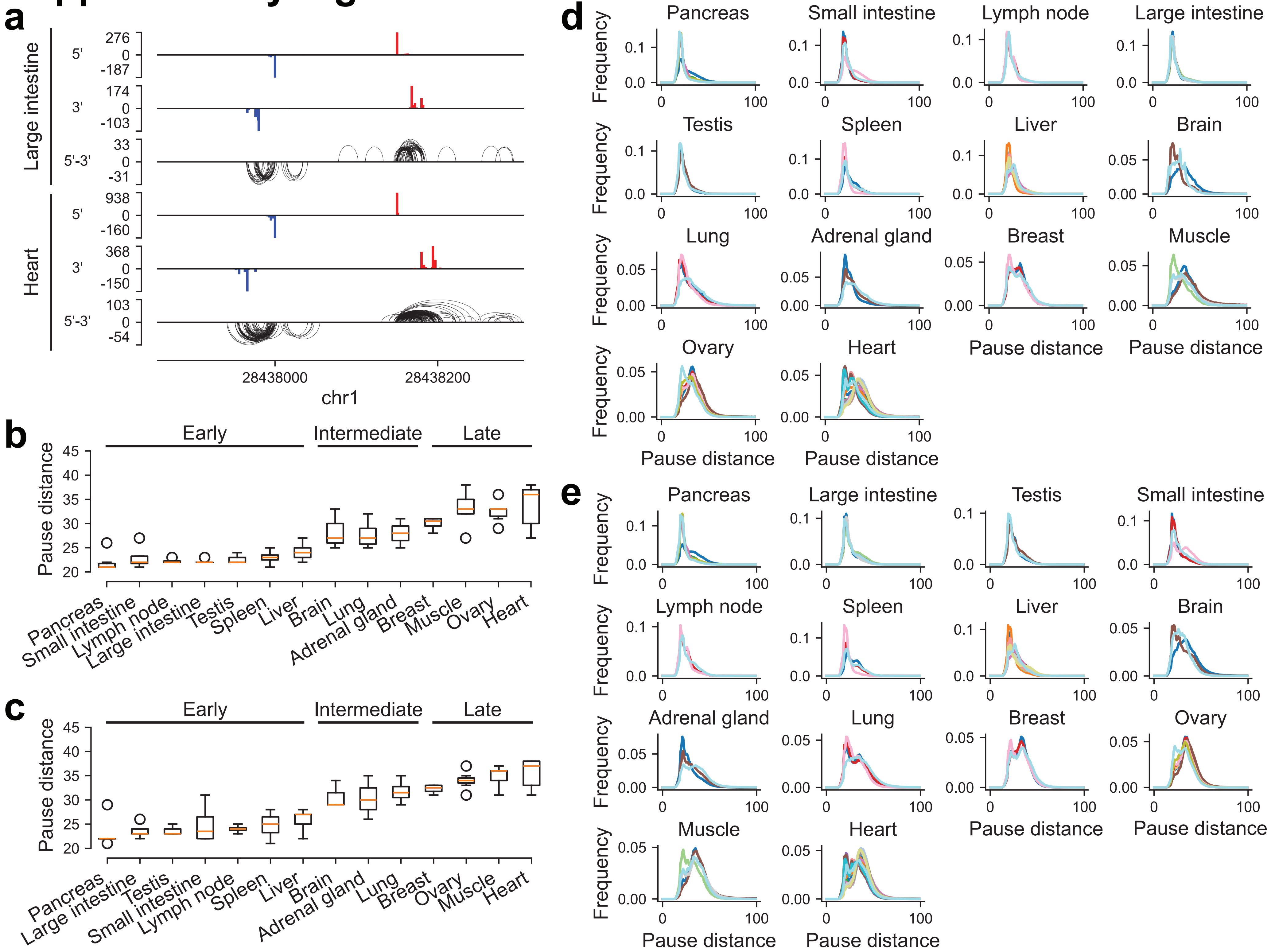

Supplementary Fig. 9 | Variation of RNAPII pause distances of transcriptional regulatory elements across tissues

(a) Representative 5’ and 3’ PRO-cap signal tracks and pause distances for a divergent element in the large intestine (top) and heart (bottom). Loops denote 5′-3′ pause distances of individual nascent RNA molecules.

(b) Boxplot summarizing median pause distances (bp) across tissues, grouped into early, intermediate, and late pausing categories, using reads mapped to distal elements.

(c) Same as (b), but for proximal elements.

(d) Distribution of pause distances (bp) across tissues, with each line representing one sample of a given tissue type, using reads mapped to distal elements.

(e) Same as (d), but for proximal elements.

Supplementary Figure 10

a

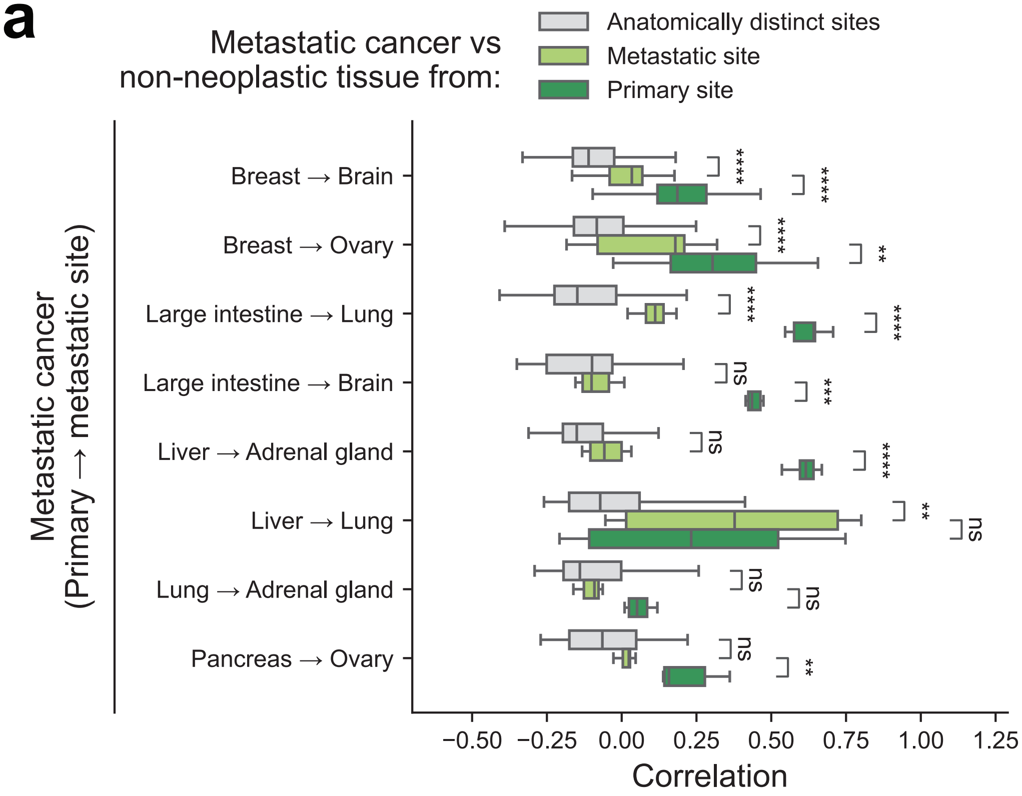

b

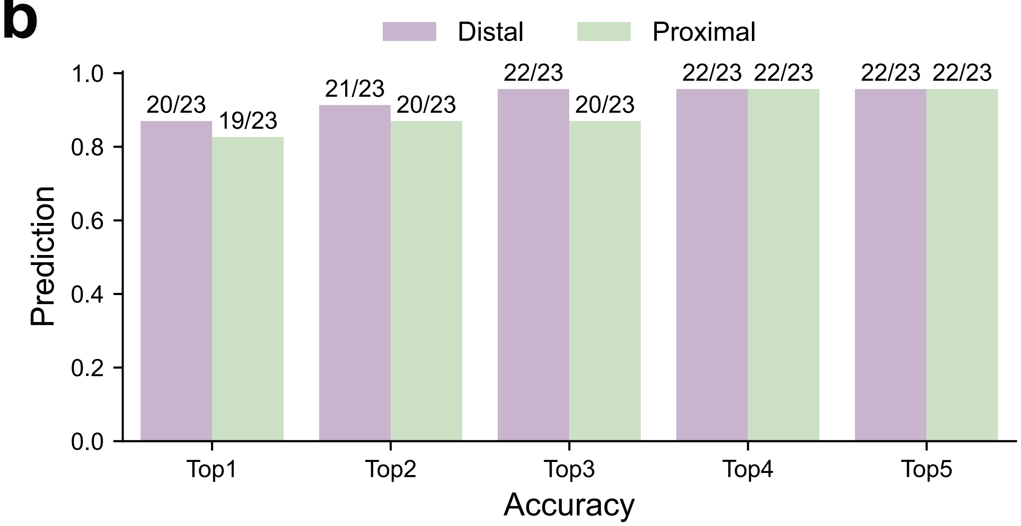

Supplementary Fig. 10 | Transcriptional regulatory element signatures predict the tissue of origin in patient-derived tumors

(a) Pairwise comparisons of divergent proximal TRE expression profiles between metastatic tumors and non-neoplastic tissues from the primary, metastatic, and unrelated sites.

(b) Accuracy of tissue-of-origin prediction for metastatic tumors using models trained on divergent distal and proximal PRO-cap profiles. Results are shown for top-1 through top-5 ranked predictions.

Supplementary Tables

Supplementary Table 1 | Metadata of PRO-cap libraries
Summary of biosample, experimental, and sequencing details for all libraries generated using PRO-cap. PBMC: peripheral blood mononuclear cell; T1D: type 1 diabetes; iPSC: induced pluripotent stem cell; ESC: embryonic stem cell; CML: chronic myelogenous leukemia; B-ALL: B-cell acute lymphocytic leukemia; GCB-DLBCL: germinal center B-cell diffuse large B-cell lymphoma; MCL: mantle cell lymphoma.

Supplementary Table 2 | Datasets from other assays
Details of the data sources used for tissue-matched comparisons across assays, ProCapNet model training, and E2G prediction of P-E links.

Supplementary Table 3 | Motif enrichment across different TRE sets
Summary of motif enrichment and associated statistics for three TRE set comparisons: (1) T cells from T1D patients versus healthy donors (T1D-Up and T1D-Down); (2) TRE modules 1-10 from a leukemia and lymphoma cell line panel (LL-M1 to LL-M10); and (3) CRC metastases to lung versus brain (CRC Met-Lung and CRC Met-Brain). Known motif enrichment is reported for the T1D and CRC metastasis comparisons, while de novo motif enrichment is reported for the leukemia and lymphoma panel.

Supplementary Table 4 | Primer sequences
Details of cloning primers and TRE sequences used in the section “Tissue-specific effects of disease- and trait-associated variants.”
